# Hypervalent iodine compounds for anti-Markovnikov-type iodo-oxyimidation of vinylarenes

**DOI:** 10.3762/bjoc.14.188

**Published:** 2018-08-16

**Authors:** Igor B Krylov, Stanislav A Paveliev, Mikhail A Syroeshkin, Alexander A Korlyukov, Pavel V Dorovatovskii, Yan V Zubavichus, Gennady I Nikishin, Alexander O Terent’ev

**Affiliations:** 1 N. D. Zelinsky Institute of Organic Chemistry of the Russian Academy of Sciences, 47 Leninsky prosp., 119991 Moscow, Russian Federation; 2 All-Russian Research Institute for Phytopathology, 143050 B. Vyazyomy, Moscow Region, Russian Federation; 3 Nesmeyanov Institute of Organoelement Compounds, Russian Academy of Sciences, Vavilov str., 28, 119991 Moscow, Russian Federation; 4 Pirogov Russian National Research Medical University, Ostrovitianov str., 1, 117997 Moscow, Russian Federation; 5 National Research Center “Kurchatov Institute”, Akademika Kurchatova pl., 1, 123182 Moscow, Russian Federation

**Keywords:** free radicals, hypervalent iodine, imide-*N*-oxyl radicals, iodination, *N*-hydroxyimides, oxidative functionalization

## Abstract

The iodo-oxyimidation of styrenes with the *N*-hydroxyimide/I_2_/hypervalent iodine oxidant system was proposed. Among the examined hypervalent iodine oxidants (PIDA, PIFA, IBX, DMP) PhI(OAc)_2_ proved to be the most effective; yields of iodo-oxyimides are 34–91%. A plausible reaction pathway includes the addition of an imide-*N*-oxyl radical to the double C=C bond and trapping of the resultant benzylic radical by iodine. It was shown that the iodine atom in the prepared iodo-oxyimides can be substituted by various nucleophiles.

## Introduction

The presented work opens a new chapter in the chemistry of *N*-hydroxyimides in combination with hypervalent iodine compounds with formation of imide-*N*-oxyl radicals. These radicals were used as reagents for the addition to a terminal position of the double bond of styrenes with subsequent iodination of the resulting benzylic radical.

It is important to note, that nitroxyl radicals are widely used in organic and biological chemistry, and in material design [1–3]. These radicals are applied in the development of monomolecular magnets [4–5], spintronics [2,6], magneto-LC effect studies [7–8], organic voltaic cells [9], electrodes for electrochemical synthesis [2], and as mediators of living polymerization [10–11]. In organic synthesis more stable types of *N*-oxyl radicals can be used as carbon-centered radical scavengers [12], oxidation catalysts, mainly for conversion of alcohols to carbonyl compounds [11,13–17]. Less stable imide-*N*-oxyl radicals are used as effective mediators for CH-functionalization with formation of C–C, C–O, C–S, and C–N bonds [11,16,18–42].

Phthalimide-*N*-oxyl (PINO) is one of the most known imide-*N*-oxyl radicals that is generated from an inexpensive *N*-hydroxyphthalimide (NHPI). This radical was used in various aerobic oxidations of bulk chemicals [18–19,43–44].

In the present work imide-*N*-oxyl radicals were used for the addition to the C=C bonds of styrenes with subsequent functionalization of the resulting benzylic radicals.

Recently, the precursors of *N*-oxyl radicals, such as *N*-hydroxyphthalimide (NHPI), *N*-hydroxysuccinimide (NHSI), *N*-hydroxybenzotriazole (HOBt) and hydroxamic acids, have been used in the reactions of radical oxygenation of styrenes [45]. Growth of interest is observed concerning the reactions of styrenes with imide-*N*-oxyl radicals, in which the latter add to the terminal position of the double C=C bond with the formation of stabilized benzyl radicals, which undergo the subsequent functionalization. In the presence of oxygen or *tert*-butyl hydroperoxide, oxidation proceeds to form the C–O [46–51] or the C=O [52–55] moiety. More complex reagents and reaction systems allows to form C–C [56–57] and C–N [58–59] bonds.

Among the above-mentioned methods, there are no examples of C–Hal bond formation despite the wide usage of organohalides in chemical syntheses. In the row of organohalides, iodides are the most reactive and versatile reagents for the following transformations [60].

**Scheme 1:**
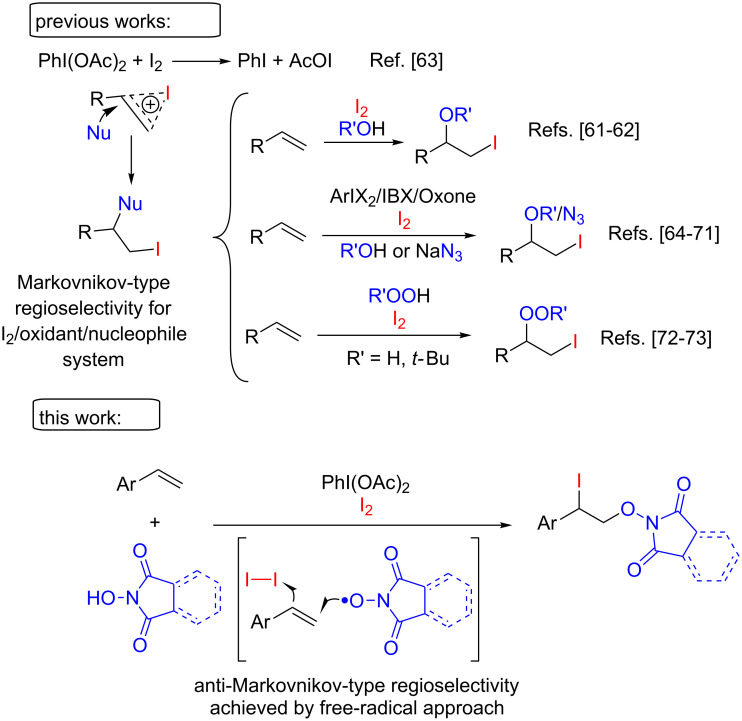
Difunctionalization of double C=C bond with the formation of C–O and C–I bonds.

One of the purposes of our work was to introduce iodine in the process of difunctionalization of styrenes with imide-*N*-oxyl radicals. Iodine atom in the product can act as a versatile leaving group for further transformations. The involvement of the iodine in the radical reactions of styrenes is complicated by the fact that unsaturated compounds readily undergo electrophilic iodination with the addition of an external nucleophile [61–62]. The oxidants used for the preparation of imide-*N*-oxyl radicals, in particular the hypervalent iodine compounds and peroxides [63–73], also generate electrophilic iodinating intermediates (Scheme 1).

For several decades, a number of papers on the electrophilic iodination of C=C bonds by iodine-containing oxidative systems with the addition of various nucleophiles have been published, all of these processes have common mechanism and the same regioselectivity. The free-radical approach developed in the present work affords the opposite (anti-Markovnikov) regioselectivity of the addition to the double bond.

## Results and Discussion

**Scheme 2:**
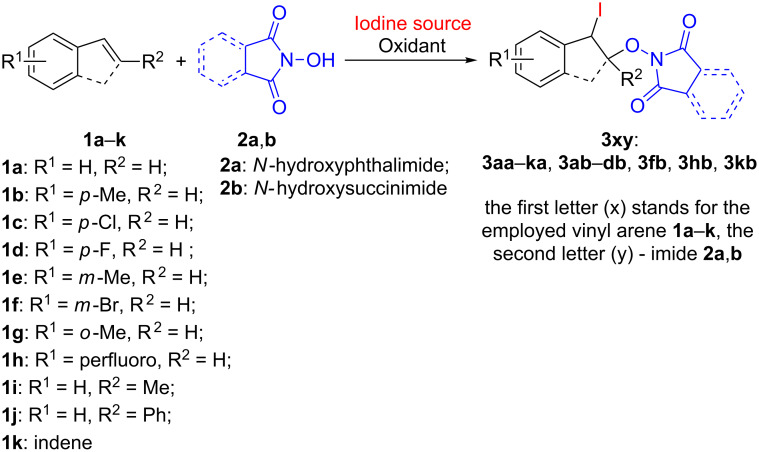
Iodo-oxyimidation of styrenes **1a**–**k** with preparation of products **3aa**–**ka**, **3ab**–**db**, **3fb**, **3hb**, and **3kb**.

In the present work, the reaction of styrenes **1a**–**k** and *N*-hydroxyimides **2a**,**b** with the formation of iodo-oxyimidated products **3aa**–**ka**, **3ab**–**db**, **3fb**, **3hb** and **3kb** was carried out (Scheme 2).

The key feature of the developed process is the non-standard regioselectivity of the formation of C–I and C–O bonds, which implies the radical pathway of the reaction.

The iodo-oxyimidation of styrenes was studied in the model reaction of styrene (**1a**) with *N*-hydroxyphthalimide (**2a**). During the optimization, the oxidant and the iodine source, as well as the nature of the solvent and the reaction time were varied (Table 1).

**Table 1:** Optimization of the synthesis of iodo-oxyimidation product **3a** from styrene **1a** and *N*-hydroxyimide **2a **^a^.

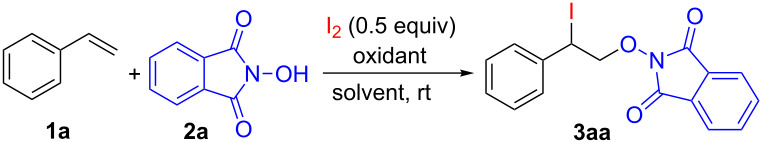				

entry	oxidant (molar ratio: mol/mol of **1a**)	solvent	time	yield of **3aa**^b^ (%)

1	PhI(OAc)_2_ (0.6)	DCM	5 min	63
2	PhI(OAc)_2_ (0.6)	DCM	10 min	90
3	PhI(OAc)_2_ (0.6)	DCM	24 h	84
4	PhI(OAc)_2_ (1.5)	DCM	10 min	73
5	PhI(OAc)_2_ (0.6)	MeCN	10 min	73
6	PhI(OAc)_2_ (0.6)	AcOH	10 min	65
7	PhI(OAc)_2_ (0.6)	PhMe	10 min	84
8^c^	PhI(OAc)_2_ (2)	DCM	10 min	7
9^d^	PhI(OAc)_2_ (2)	DCM	10 min	52
10	PhI(OCOCF_3_)_2_ (0.6)	DCM	10 min	31
11	IBX (1.0)	DCM	24 h	54
12	IBX (0.3)	DCM	24 h	32
13	DMP (0.6)	DCM	30 min	52
14	DMP (0.3)	DCM	30 min	52
15	Oxone (2)	DCM/H_2_O (2:1)	4 h	44
16	2-iodobenzoic acid (0.1), Oxone (2)	DCM/H_2_O (2:1)	4 h	44
17	TBHP (70% aq) (2)	DCM	12 h	ND
18	TBHP (70% aq) (2)	MeCN	12 h	ND
19	TBHP (70% aq) (2)	AcOH	12 h	ND
20	TBAI (0.1), TBHP (70% aq) (2)	MeCN	12 h	ND
21	(NH_4_)_2_S_2_O_8_ (1.5)	DCM/H_2_O (2:1)	12 h	5
22	DDQ (2)	MeCN	30 min	5

^a^Reaction conditions: **1a** (1 mmol), **2a** (1 mmol), I_2_ (0.5 mmol), oxidant (0.3–2 mmol), solvent (6.0 mL), 20–25 °C, 5 min–24 h, under air. For entries where a mixture of solvents was used, the v/v ratio is given in parentheses. ^b^Isolated yield. ND = not detected. ^c^NaI·2H_2_O (1 mmol) was employed instead of I_2_. ^d^TBAI (1 mmol) was employed instead of I_2_.

In general, the iodo-oxyimidation reaction is characterized by the following: The product **3aa** is formed regardless what kind of hypervalent iodine compound is used (Table 1, entries 1-14) and Oxone (Table 1, entries 15 and 16) as the oxidant. The best yield of **3aa** (90%) was obtained using PhI(OAc)_2_ (Table 1, entry 2). Other iodine-based oxidants, such as PhI(OCOCF_3_)_2_ (Table 1, entry 10, yield 31%), IBX (Table 1, entries 11 and 12, yield 32–54%), DMP (Table 1, entries 13–14, yield 52%), showed less efficacy in this process. Peroxide oxidants, such as TBHP, TBAI/TBHP system [74], (NH_4_)_2_S_2_O_8_, and DDQ were ineffective in the studied process (Table 1, entries 17–22). A satisfactory yield of **3aa** (44%) was achieved using Oxone as the oxidant (Table 1, entry 15). The addition of a catalytic amount of 2-iodobenzoic acid, which forms hypervalent iodine compounds in the presence of Oxone [75], did not lead to an increased yield of **3aa** (Table 1, entry 16).

Dichloromethane proved to be the best solvent for the reaction, as carrying out the reaction in other solvents led to a decrease in the yield of **3aa** (Table 1, entries 5–7). Increasing the amount of PhI(OAc)_2_ from 0.6 mmol to 1.5 mmol (Table 1, entry 4) leads to a decrease in the yield of **3aa** presumably due to the enhancing the role of side oxidation processes. The optimal reaction time was 10 min, a reaction of 5 min resulted in a significant decrease in the yield of the desired product (Table 1, entry 1). Prolongation of the reaction time to 24 h led to a slight decrease in the yield of **3aa** (Table 1, entry 3) due to its instability under the reaction conditions.

The possibility of using iodide salts (NaI and TBAI) was shown in Entries 8 and 9, however, the yield of **3aa** in that cases is lower than in the case of molecular iodine.

**Figure 1:**
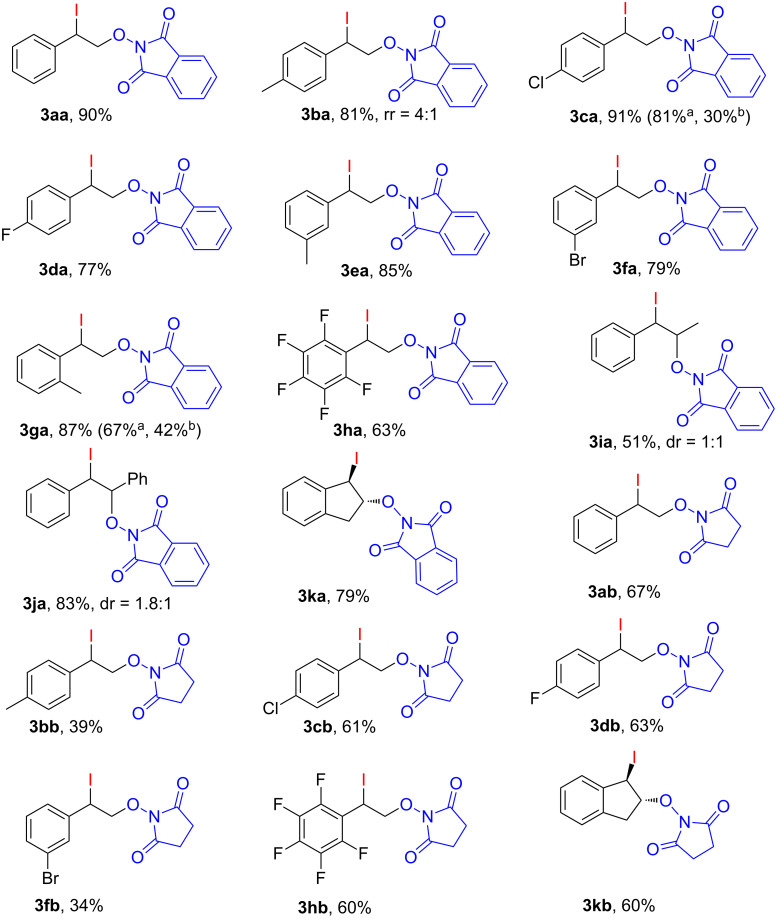
Scope of the iodo-oxyimidation of vinylarenes with I_2_/PhI(OAc)_2_ system. Reaction conditions: vinylarene **1a**–**k** (0.5 mmol), *N*-hydroxyimide **2a**,**b** (0.5 mmol), I_2_ (0.25 mmol), PhI(OAc)_2_ (0.3 mmol), DCM (3.0 mL), 20–25 °C, 10 min, under air. rr = regioisomers ratio. ^a^IBX (0.5 mmol) was used instead of PhI(OAc)_2_, reaction time: 24 h. ^b^DMP (0.15 mmol) was used instead of PhI(OAc)_2_, reaction time 30 min.

In the optimized reaction conditions (Table 1, entry 2) iodo-oxyimidation of various vinylarenes were performed in order to study the scope of the developed method (Figure 1).

The iodo-oxyimidation successfully proceeded using styrenes having both electron-withdrawing (Cl, F, Br) substituents in the aromatic ring (products **3ca**, **3da**, **3fa**, **3ha**, **3cb**–**hb**, yield 34–91%), and an electron-donating methyl group (products **3ba**, **3ea**, **3ga**, **3bb**, yield 39–85%). Good yields (60–79%) were achieved with a cyclic analogue of styrene – indene (**1k**, compounds **3ka**, **3kb**). β-Substituted styrenes (β-methyl styrene (**1i**) and (*E*)-stilbene (**1j**) also underwent the studied transformation giving iodo-oxyimides **3ia** (yield 51%) and **3ja** (yield 83%). The reaction of NHPI (**2a**) with *p*-methoxystyrene under standard conditions led to a complex mixture of products, possibly due to an increased tendency of the substrate to electrophilic addition of iodine. The use of allylbenzene in the reaction did not result in the formation of the desired iodo-oxyimide, presumably due to a side process of oxidation of the allylic methylene fragment. The use of *N*-hydroxyphthalimide (**2a**) gives iodo-oxymidation products with yields generally higher (25% on average) than that of *N*-hydroxysuccinimide (**2b**).

**Figure 2:**
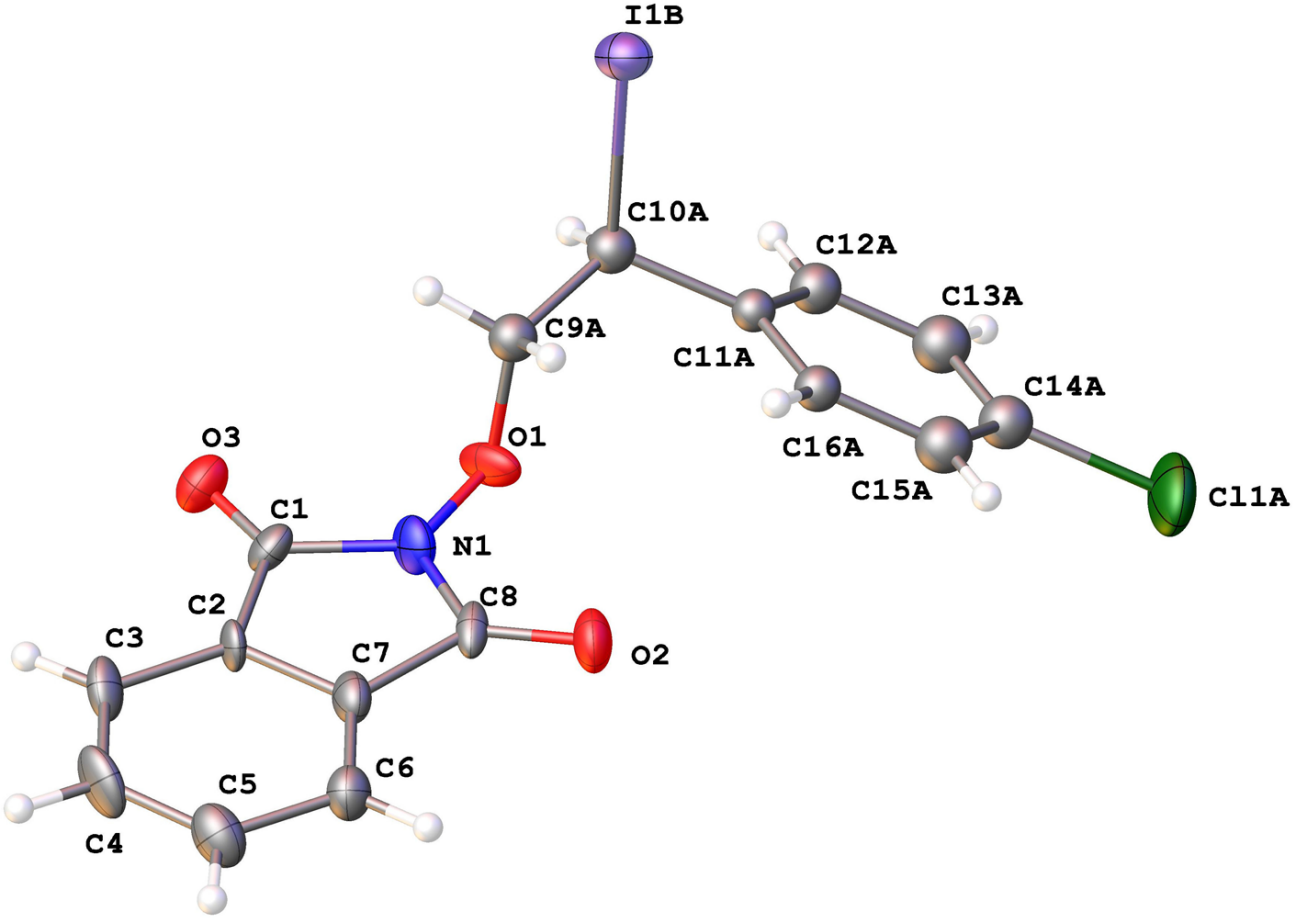
Molecular structure of **3ca**. Atoms are presented as anisotropic displacement parameters (ADP) ellipsoids (50% probability). For clarity, only one set of positions of the disordered ethylene bridge and Ph groups is shown.

Structures of the iodo-oxyimides **3aa**–**ka**, **3ab**–**db**, **3fb**, **3hb** and **3kb** were confirmed by 1D and 2D NMR spectroscopy, IR spectroscopy, high-resolution mass spectrometry and elemental analysis. An additional confirmation of the structure of **3ca** was made using X-ray crystallographic analysis (Figure 2). Details of the data collection and refinement are provided in Supporting Information File 1 and can be obtained free of charge via the web at https://www.ccdc.cam.ac.uk/structures/ (CCDC-1845323).

### Proposed mechanism of the iodo-oxyimidation

**Scheme 3:**
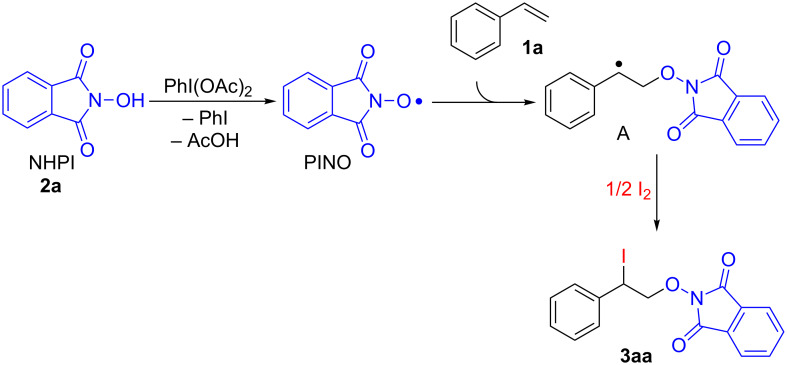
The proposed mechanism of iodo-oxyimidation of styrene (**1a**) using the NHPI/I_2_/PhI(OAc)_2_ system with the formation of product **3aa**.

Based on the literature data describing the formation of the phthalimide-*N*-oxyl radical (PINO) from NHPI under the action of PhI(OAc)_2_ [34,55,59,76–77], and based on information about the reaction of the PINO radical with styrenes [45] and interaction of benzyl radicals with iodine [78–79], a mechanism for the reaction of iodo-oxyimidation of styrenes under the action of the NHPI/I_2_/PhI(OAc)_2_ system was proposed (Scheme 3).

On the first step, NHPI (**2a**) is oxidized by PhI(OAc)_2_ to form PINO radical. The addition of PINO to the double C=C bond of styrene (**1a**) leads to the formation of intermediate **A**. At the final step the iodine traps benzyl radical **A** to form the final product **3aa**.

### Electrochemical studies

**Figure 3:**
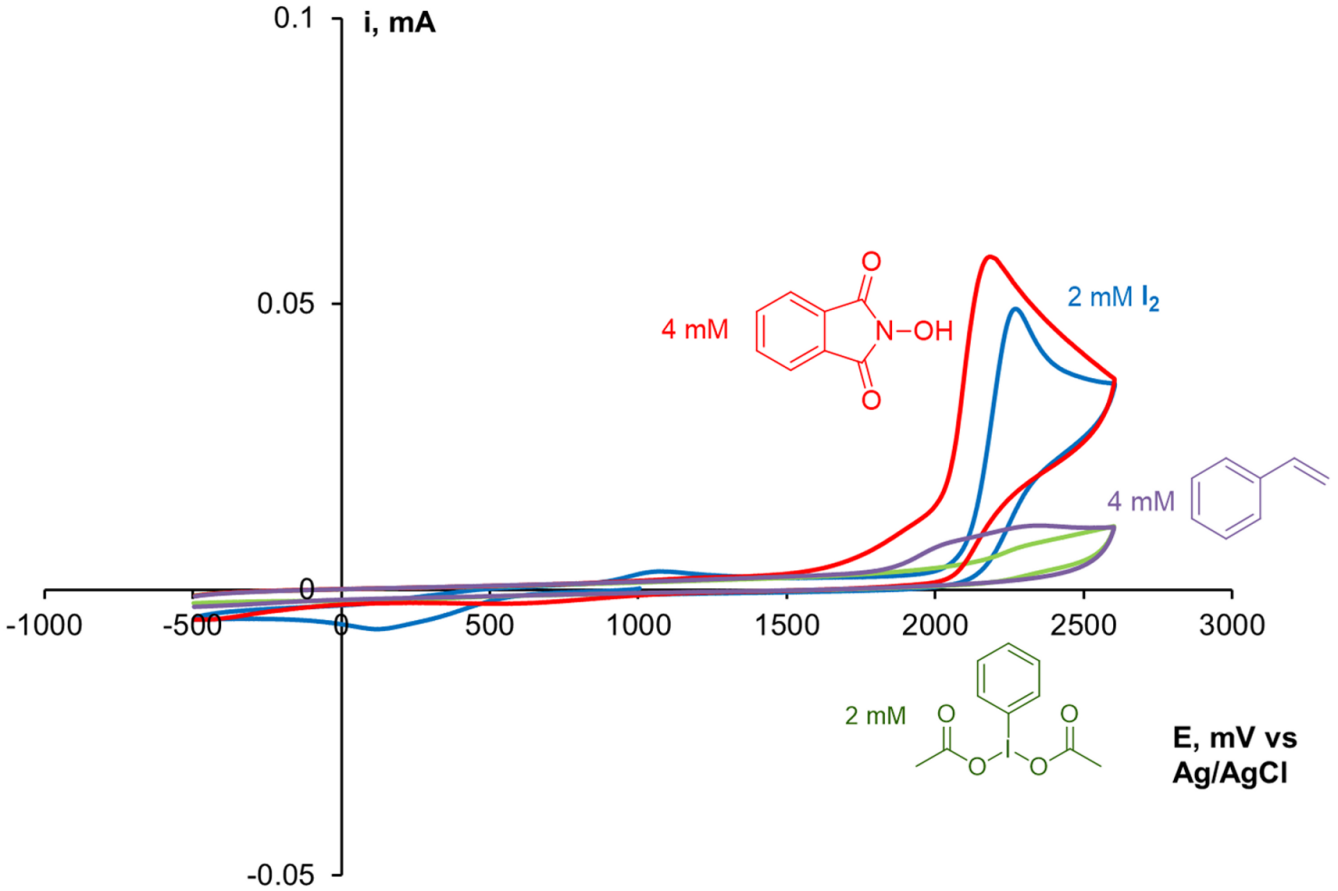
CV curves of styrene (**1a**, purple), NHPI (**2a**, red), I_2_ (blue) and PhI(OAc)_2_ (green) in 0.1 M *n*-Bu_4_NBF_4_/MeCN at a scan rate of 100 mV/s on a working glassy-carbon electrode.

Cyclic voltammetry (CV) experiments on a working glassy-carbon electrode were carried out for deeper understanding of the plausible reaction mechanism. As CH_2_Cl_2_ is not suitable as a solvent due to the poor solubility of NHPI, thus MeCN was used. Tetrabutylammonium tetrafluoroborate, which cannot be oxidized in such experimental conditions [80], was chosen as a supporting electrolyte. Cyclic voltammograms of styrene (**1a**), NHPI (**2a**), I_2_ and PhI(OAc)_2_ in MeCN solution were registered (Figure 3).

The NHPI oxidation peak is observed at +2.18 V, whereas iodine is oxidized at slightly higher potential (+2.27 V), and styrene oxidation peak is not so pronounced. Therefore, we can conclude that under experimental conditions NHPI is oxidized preferentially over iodine providing PINO radicals that leads to the observed regioselectivity. The contribution of the oxidation of styrene to the overall process is unlikely.

### Practical application of the iodo-oxyimidation

**Scheme 4:**
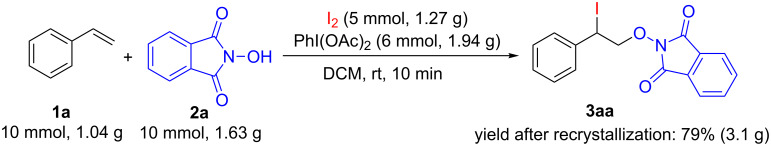
Gram-scale synthesis of compound **3aa**.

The applicability of the developed method for the gram-scale preparation was demonstrated by the synthesis of **3aa** (3.1 g, 79%) without column chromatography (Scheme 4).

**Scheme 5:**
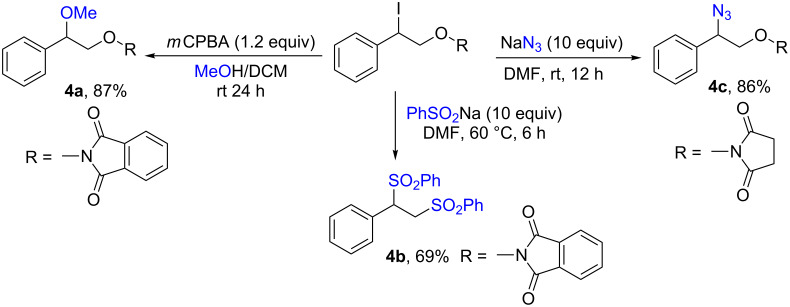
Synthetic utility of the iodo-oxyimides **3aa** and **3ab**.

The synthetic utility of the obtained products **3aa** and **3ab** was demonstrated by the substitution of the iodine atom with O- (methanol), S- (benzenesulfinate) and N- (azide) nucleophiles (Scheme 5).

It is noteworthy that the reaction of compound **3aa** with sodium benzenesulfinate results in the nucleophilic substitution of both the iodine atom and the oxyphthalimide moiety to form a vicinal disulfone **4b**.

## Conclusion

Iodo-oxyimidation of vinylarenes using *N*-hydroxyphthalimide and *N*-hydroxysuccinimide was developed. PhI(OAc)_2_ was the best oxidant for the synthesis of the target products (yields up to 91%). In contrast to previous studies in which oxidants promote the electrophilic addition of iodine to the C=C bond, radical addition predominates in the discovered process. Radical pathway starts from the attack of imide-*N*-oxyl radicals on the double C=C bond, which allows for anti-Markovnikov type regioselectivity of C–O and C–I bond formation. Electrochemical mechanistic studies based on cyclic voltammetry (CV) data confirm proposed reaction mechanism. Possible ways of using the obtained iodo-oxyimidated products via substitution of iodine atom were demonstrated.

## Experimental

### General procedure for the synthesis of compounds **3aa**–**ka**, **3ab**–**db**, **3fb**, **3hb** and **3kb** (Figure 1)

Iodine (64 mg, 0.25 mmol) was added to a stirred mixture of vinylarene **1a**–**k** (52–97 mg, 0.50 mmol) and *N*-hydroxyimide **2a**,**b** (58–82 mg, 0.50 mmol) in CH_2_Cl_2_ (3 mL) at 20–25 °С. Then, PhI(OAc)_2_ (97 mg, 0.30 mmol) was added. In the additional experiments compounds **3ca** and **3ga** were prepared using IBX (140 mg, 0.50 mmol) or DMP (64 mg, 0.15 mmol) instead of PhI(OAc)_2_. After stirring for 10 min under air atmosphere at 20–25 °С, CH_2_Cl_2_ (30 mL) was added. The resulting mixture was washed with an aqueous solution of Na_2_S_2_O_3_·5H_2_O (200 mg in 20 mL of water), saturated aqueous NaHCO_3_ solution (20 mL), and with water (20 mL), dried over anhydrous MgSO_4_ and filtered. CH_2_Cl_2_ was evaporated at 20–25 °С under water-jet vacuum (20–30 mm Hg). Products **3aa**–**ka**, **3ab**–**db**, **3fb**, **3hb** and **3kb** were isolated by column chromatography on silica gel using with EtOAc/DCM eluent (with the volume part of EtOAc gradually increased from 0% to 2.5%).

Iodo-oxyimides **3aa**–**ka**, **3ab**–**db**, **3fb**, **3hb** and **3kb** should be stored in a freezer and handled with minimal heat due to their instability at elevated temperatures.

## Supporting Information

File 1 Experimental procedures, characterization data, copies of ^1^H, ^13^C and ^19^F NMR spectra, copies of HRMS and FT-IR spectra and the ORTEP diagram and X-ray data for compound **3ca**.

File 2 X-ray structure analysis data for **3ca** (CCDC-1845323).
