# Supplementary material for: Hypervalent iodine compounds for anti-Markovnikov-type iodo-oxyimidation of vinylarenes
Source: Beilstein J Org Chem. 2018 Aug 16;14:2146–55. doi: 10.3762/bjoc.14.188 (PMC6122379; doi:10.3762/bjoc.14.188)
Supplement: File 1 — Experimental procedures, characterization data, copies of 1H, 13C and 19F NMR spectra, copies of HRMS and FT-IR spectra and the ORTEP diagram and X-ray data for compound 3ca. [file Beilstein_J_Org_Chem-14-2146-s001.pdf]

Supporting Information

for

Hypervalent iodine compounds for anti-Markovnikov-type iodo-oxyimidation of vinylarenes

Igor B. Krylov[1,2], Stanislav A. Paveliev[1], Mikhail A. Syroeshkin[1], Alexander A. Korlyukov[3,4], Pavel V. Dorovatovskii[5], Yan V. Zubavichus[5], Gennady I. Nikishin[1] and Alexander O. Terent'ev[1,2]*

Address: [1]N. D. Zelinsky Institute of Organic Chemistry of the Russian Academy of Sciences, 47 Leninsky prosp., 119991 Moscow, Russian Federation, [2]All-Russian Research Institute for Phytopathology, 143050 B. Vyazyomy, Moscow Region, Russian Federation, [3]Nesmeyanov Institute of Organoelement Compounds, Russian Academy of Sciences, Vavilov str., 28, 119991 Moscow, Russian Federation, [4]Pirogov Russian National Research Medical University, Ostrovitianov str., 1, 117997 Moscow, Russian Federation and [5] National Research Center "Kurchatov Institute", Akademika Kurchatova pl., 1, 123182 Moscow, Russian Federation

Email: Alexander Olegovich Terent'ev* - terentev@ioc.ac.ru

*Corresponding author

Experimental procedures, characterization data, copies of spectra and the ORTEP diagram and X-ray data for compound 3ca

Table of contents

Experimental details	…………	S3
NMR spectra of compounds **3aa–ka, 3ab–db, 3fb, 3hb, 3kb, 4a-c**	…………	S13
HRMS spectra of compounds **3ga, 3ia, 3cb, 4a–c**	…………	S65
FT-IR spectra for compound **3aa–ka, 3ab–db, 3fb, 3hb, 3kb, 4a–c**	…………	S71
Structure Determination by X-ray crystallography	…………	S92

General methods

^{1}H, ^{13}C and ^{19}F NMR spectra were recorded on a Bruker AVANCE II 300 spectrometer (300.13 MHz, 75.47 MHz and 282.40 MHz, respectively) in $CDCl_3$ and DMSO-d_6. ^{1}H-^{15}N HMBC NMR spectra were recorded on a Bruker AVANCE III 400 spectrometer (400.13 MHz and 40.56 Hz, respectively). Chemical shifts were reported in parts per million (ppm), and the residual solvent peak was used as an internal reference: ^{1}H ($CDCl_3$ δ = 7.26 ppm, DMSO-d_6 δ = 2.50 ppm), ^{13}C ($CDCl_3$ δ = 77.16 ppm, DMSO-d_6 δ = 39.52 ppm). Multiplicity was indicated as follows: s (singlet), d (doublet), t (triplet), q (quartet), dd (doublet of doublets), dt (doublet of triplets), td (triplet of doublets), m (multiplet).

High resolution mass spectra (HR-MS) were measured on a Bruker maXis instrument using electrospray ionization (ESI). The measurements were performed in the positive ion mode (interface capillary voltage – 4500 V) at a mass range from *m*/*z* 50 to *m*/*z* 3000 Da and external calibration with Electrospray Calibrant Solution (Fluka). A syringe injection was used for all acetonitrile solutions (flow rate 3 μL/min). Nitrogen was applied as a drying gas and the interface temperature was set at 180 °C. FT-IR spectra were recorded on a Bruker ALPHA FT-IR spectrometer.

Materials

Column chromatography was performed using silica gel (0.060–0.200 mm, 60 Å, Acros). Dichloromethane (DCM) and hexane were distilled over K_2CO_3. Acetonitrile (MeCN) and ethyl acetate (EtOAc) were distilled over P_2O_5. Toluene (PhMe) was distilled over sodium metal. Methanol (MeOH) was distilled over magnesium turnings. Glacial acetic acid (AcOH) and *N,N*-dimethylformamide (DMF) were used as received from commercial sources.

Vinylarenes **1a–k**, *N*-hydroxyphthalimide (**2a**, NHPI), *N*-hydroxysuccinimide (**2b**, NHSI), (diacetoxyiodo)benzene ($PhI(OAc)_2$), 2-iodobenzoic acid, Oxone, *tert*-butylhydroperoxide (70% aqueous solution) (TBHP), sodium iodide dihydrate ($NaI{\cdot}2H_2O$), tetrabutylammonium iodide (TBAI), ammonium peroxydisulfate ($(NH_4)_2S_2O_8$), 2,3-dichloro-5,6-dicyano-1,4-benzoquinone (DDQ), *m*-chloroperoxybenzoic acid (70–75%, balance 3-chlorobenzoic acid and water) (*m*-CPBA), sodium benzenesulfinate ($PhSO_2Na$) and sodium azide (NaN_3) were commercial reagents (Acros, Sigma, Alfa Aesar). [Bis(trifluoroacetoxy)iodo]benzene ($PhI(OCOCF_3)_2$), 2-iodoxybenzoic acid (IBX) and Dess–Martin periodinane (DMP) were synthesized according to the literature [1-3].

Experimental for Table 1

Iodine (127 mg, 0.5 mmol) was added to a stirred mixture of styrene **1a** (104 mg, 1 mmol) and *N*-hydroxyphthalimide **2a** (163 mg, 1 mmol) in solvent (6 mL) at 20–25 °C. Then, oxidant ($PhI(OAc)_2$, $PhI(OCOCF_3)_2$, IBX, DMP, Oxone, TBHP, $(NH_4)_2S_2O_8$ or DDQ, 84-948 mg, 0.6-3 mmol) was added. At entry 8, $NaI{\cdot}2H_2O$ (186 mg, 1 mmol) was employed instead of I_2. In entry 9, TBAI (369 mg, 1 mmol) was employed instead of I_2. In entry 16 in addition to Oxone 2-iodobenzoic acid (12 mg, 0.05 mmol) was added. In entry 20 in addition to TBHP TBAI (16 mg, 0.05 mmol) was added. After stirring the reaction mixture under air atmosphere at 20–25 °C for 5 min – 24 h, DCM (30 mL) was added. The mixture was washed with aqueous solution of $Na_2S_2O_3{\bullet}5H_2O$ (200 mg in 20 mL of water), saturated aqueous $NaHCO_3$ solution (20 mL), then with water (20 mL), dried over anhydrous $MgSO_4$ and filtered. DCM was rotary evaporated at 20–25 °C under water-jet vacuum (20–30 mmHg). Product **3aa** was isolated by column chromatography on silica gel using EtOAc – DCM eluent (2.5% volume part of EtOAc).

2-(2-Iodo-2-phenylethoxy)isoindoline-1,3-dione (3aa). White solid: 90% yield (176 mg); mp = 135-136 °C. ^{1}H NMR (300.13 MHz, $CDCl_3$): δ = 7.85-7.68 (m, 4H), 7.54 (d, *J* = 7.4 Hz, 2H), 7.36-7.17 (m, 3H), 5.53 (dd, J_1 = 9.8 Hz, J_2 = 5.7 Hz, 1H), 5.00-4.88 (m, 1H), 4.71 (dd, J_1 = 10.7 Hz, J_2 = 5.7 Hz, 1H). ^{13}C NMR (75.47 MHz, $CDCl_3$): δ = 163.3, 139.8, 134.7, 128.9, 128.7, 128.6, 127.9, 123.7, 81.4, 25.2. ^{15}N NMR (40.56 MHz, $CDCl_3$): δ = -163.0. IR (KBr) ν(cm^{-1}): = 1731, 1720, 1465, 1192, 1138, 1127, 1114, 1081, 1020, 988, 875, 716, 696, 519. Anal. Calcd for $C_{16}H_{12}INO_3$: C, 48.88; H, 3.08; N, 3.56. Found: C, 48.73; H, 2.95; N, 3.56.

General procedure for synthesis of compounds 3aa–ka, 3ab–db, 3fb, 3hb and 3kb (experimental for Figure 1)

Iodine (64 mg, 0.25 mmol) was added to a stirred mixture of vinylarene **1a–k** (52-97 mg, 0.50 mmol) and *N*-hydroxyimide **2a**,**b** (58-82 mg, 0.50 mmol) in DCM (3 mL) at 20–25 °C. Then, $PhI(OAc)_2$ (97 mg, 0.30 mmol) was added. In the additional experiments compounds **3ca** and **3ga** were prepared using IBX (140 mg, 0.50 mmol) or DMP (64 mg, 0.15 mmol) instead of $PhI(OAc)_2$. After stirring for 10 min under air atmosphere at 20-25 °C DCM (30 mL) was added and the mixture was washed with aqueous solution of $Na_2S_2O_3{\cdot}5H_2O$ (200 mg in 20 mL of water), saturated aqueous $NaHCO_3$ solution (20 mL), then with water (20 mL), dried over anhydrous $MgSO_4$ and filtered. DCM was rotary evaporated at 20–25 °C under water-jet vacuum (20–30 mmHg). Products **3aa–ka,**

3ab–db, 3fb, 3hb and **3kb** were isolated by column chromatography on silica gel using with EtOAc – DCM eluent (with the volume part of EtOAc gradually increased from 0% to 2.5%).

Iodo-oxyimides **3aa–ka, 3ab–db, 3fb, 3hb** and **3kb** were stored in the freezer and handled with minimal heat due to their potential instability at elevated temperature, which was indicated by the darkening both pure powders and solutions when standing at room temperature for even a few hours.

2-(2-Iodo-2-(p-tolyl)ethoxy)isoindoline-1,3-dione (mixture of regioisomers 4:1) (3ba). White solid: 81% yield (176 mg); mp = 135-136 °C. ^{1}H NMR (300.13 MHz, $CDCl_3$): δ = 7.84-7.66 (m, 4H), 7.47-7.35 (m, 2H), 7.17 (d, J = 7.8 Hz, 0.4H), 7.09 (d, J = 7.9 Hz, 1.6H), 5.56-5.45 (m, 1H), 4.97-4.87 (m, 0.8H), 4.67 (dd, J_1 = 10.7 Hz, J_2 = 5.6 Hz, 0.8H), 3.73 (dd, J_1 = 10.4 Hz, J_2 = 6.2 Hz, 0.2H), 3.57 (dd, J_1 = 10.4 Hz, J_2 = 8.0 Hz, 0.2H), 2.33 (s, 0.6H), 2.25 (s, 2.4H). ^{13}C NMR (75.47 MHz, $CDCl_3$): δ = 163.3, 138.7, 136.8, 134.7, 129.7, 128.7, 127.6, 123.7, 81.3, 25.6, 21.3. ^{15}N NMR (40.56 MHz, $CDCl_3$): δ = -163.1. IR (KBr) ν(cm^{-1}): 1786, 1726, 1375, 1359, 1187, 1129, 1113, 1081, 978, 877, 699. Anal. Calcd for $C_{17}H_{14}INO_3$: C, 50.14; H, 3.47; N, 3.44. Found: C, 50.15; H, 3.48; N, 3.43.

2-(2-(4-Chlorophenyl)-2-iodoethoxy)isoindoline-1,3-dione (3ca). White solid: 91% yield (194 mg); mp = 137-138 °C. ^{1}H NMR (300.13 MHz, $CDCl_3$): δ = 7.87-7.70 (m, 4H), 7.50 (d, J = 8.5 Hz, 2H), 7.30 (d, J = 8.4 Hz, 2H), 5.50 (dd, J_1 = 10.2 Hz, J_2 = 5.3 Hz, 1H), 4.97-4.86 (m, 1H), 4.67 (dd, J_1 = 10.7 Hz, J_2 = 5.3 Hz, 1H). ^{13}C NMR (75.47 MHz, $CDCl_3$): δ = 163.3, 138.5, 134.8, 134.4, 129.3, 129.2, 128.8, 123.7, 81.2, 23.7. ^{15}N NMR (40.56 MHz, $CDCl_3$): δ = -163.7. IR (KBr) ν(cm^{-1}): 1787, 1727, 1492, 1469, 1392, 1374, 1360, 1188, 1133, 1081, 1014, 977, 933, 877, 836, 787, 726, 698. Anal. Calcd for $C_{16}H_{11}ClINO_3$: C, 44.94; H, 2.59; N, 3.28. Found: C, 44.91; H, 2.55; N, 3.22.

2-(2-(4-Fluorophenyl)-2-iodoethoxy)isoindoline-1,3-dione (3da). White solid: 77% yield (158 mg); mp = 153-154 °C. ^{1}H NMR (300.13 MHz, $CDCl_3$): δ = 7.87-7.68 (m, 4H), 7.53 (dd, J_1 = 8.6 Hz, J_2 = 5.3 Hz, 2H), 7.00 (t, J = 8.6 Hz, 2H), 5.50 (dd, J_1 = 10.1 Hz, J_2 = 5.4 Hz, 1H), 4.88 (m, 1H), 4.66 (dd, J_1 = 10.7 Hz, J_2 = 5.4 Hz, 1H). ^{13}C NMR (75.47 MHz, $CDCl_3$): δ = 163.3, 162.5 (d, J = 248.6 Hz), 135.8 (d, J = 3.4 Hz), 134.8, 129.8 (d, J = 8.4 Hz), 128.8, 123.7, 115.9 (d, J = 21.8 Hz), 81.5, 24.1. ^{19}F NMR (282.40 MHz, $CDCl_3$): δ = -112.5 (1F). IR (KBr) ν(cm^{-1}): 1732, 1720, 1509, 1464, 1236, 1138,

1125, 986, 875, 837, 696. Anal. Calcd for $C_{16}H_{11}FINO_3$: C, 46.74; H, 2.70; N, 3.41. Found: C, 46.51; H, 2.71; N, 3.29.

2-(2-Iodo-2-(m-tolyl)ethoxy)isoindoline-1,3-dione (3ea). White solid: 85% yield (173 mg); mp = 123-124 °C. ^{1}H NMR (300.13 MHz, $CDCl_3$): δ = 7.82-7.67 (m, 4H), 7.34-7.27 (m, 2H), 7.20-7.13 (m, 1H), 6.98 (d, J = 7.4 Hz, 1H), 5.48 (dd, J_1 = 9.7 Hz, J_2 = 5.7 Hz, 1H), 4.97-4.88 (m, 1H), 4.67 (dd, J_1 = 10.7 Hz, J_2 = 5.7 Hz, 1H), 2.30 (s, 3H). ^{13}C NMR (75.47 MHz, $CDCl_3$): δ = 163.2, 139.7, 138.6, 134.7, 129.5, 128.9, 128.8, 128.4, 125.0, 123.6, 81.3, 25.5, 21.45. IR (KBr) ν(cm^{-1}): 1722, 1467, 1361, 1188, 1124, 983, 874, 789, 719, 699, 518. Anal. Calcd for $C_{17}H_{14}INO_3$: C, 50.14; H, 3.47; N, 3.44. Found: C, 50.21; H, 3.31; N 3.31.

2-(2-(3-Bromophenyl)-2-iodoethoxy)isoindoline-1,3-dione (3fa). White solid: 79% yield (186 mg); mp = 134-135 °C. ^{1}H NMR (300.13 MHz, $CDCl_3$): δ = 7.84-7.70 (m, 4H), 7.69-7.64 (m, 1H), 7.50-7.44 (m, 1H), 7.36-7.29 (m, 1H), 7.22-7.14 (m, 1H), 5.42 (dd, J_1 = 5.4 Hz, J_2 = 10.1 Hz, 1H), 4.93-4.84 (m, 1H), 4.65 (dd, J_1 = 10.8 Hz, J_2 = 5.4 Hz, 1H). ^{13}C NMR (75.47 MHz, $CDCl_3$): δ = 163.2, 142.1, 134.8, 131.7, 131.0, 130.5, 128.7, 126.7, 123.8, 122.7, 81.2, 23.2. IR (KBr) ν(cm^{-1}): 1720, 1464, 1369, 1189, 1134, 1123, 1080, 1069, 873, 787, 697, 519. Anal. Calcd for $C_{16}H_{11}BrINO_3$: C, 40.71; H, 2.35; N, 2.97. Found: C, 40.92; H, 2.32; N 2.95.

2-(2-Iodo-2-(o-tolyl)ethoxy)isoindoline-1,3-dione (3ga). White solid: 87% yield (177 mg); mp = 112-113 °C. ^{1}H NMR (300.13 MHz, $CDCl_3$): δ = 7.82-7.69 (m, 4H), 7.59 (d, J = 7.5 Hz, 1H), 7.21-7.09 (m, 3H), 5.69 (dd, J_1 = 9.7 Hz, J_2 = 5.9 Hz, 1H), 5.11-5.02 (m, 1H), 4.75 (dd, J_1 = 10.6 Hz, J_2 = 5.9 Hz, 1H), 2.40 (s, 3H). ^{13}C NMR (75.47 MHz, $CDCl_3$): δ = 163.2, 137.5, 136.3, 134.7, 131.3, 128.8 128.6, 126.8, 126.1, 123.7, 80.4, 22.4, 19.3. IR (KBr) ν(cm^{-1}): 1791, 1720, 1542, 1465, 1190, 1128, 1019, 984, 875, 722, 697, 518. HRMS (ESI) Calcd for $C_{17}H_{14}INO_3$ $[M + NH_4]^+$ = 425.0357, Found 425.0352.

2-(2-Iodo-2-(perfluorophenyl)ethoxy)isoindoline-1,3-dione (3ha). White solid: 63% yield (152 mg); mp = 140-141 °C. ^{1}H NMR (300.13 MHz, $CDCl_3$): δ = 7.89-7.71 (m, 4H), 5.65 (dd, J_1 = 11.0 Hz, J_2 = 5.8 Hz, 2H), 5.02-4.90 (t, J = 11.0 Hz, 1H), 4.70 (dd, J_1 = 11.0 Hz, J_2 = 5.8 Hz, 1H). ^{13}C NMR (75.47 MHz, $CDCl_3$): δ = 163.3, 135.0, 128.8, 123.9, 114.1 (td, J_1 = 14.8 Hz, J_2 = 4.6 Hz), 79.8 (t, J = 3.9 Hz), 6.4. ^{19}F NMR (282.40 MHz, $CDCl_3$): δ = -140.1 (bs, 2F), -153.1 (t, J = 20.9 Hz, 1F) -161.2 (td, J_1 = 21.4 Hz, J_2

= 6.9 Hz, 2F). IR (KBr) ν(cm^{-1}): 1735, 1723, 1525, 1505, 1468, 1192, 1145, 1126, 1040, 1019, 994, 970, 909, 877, 697. Anal. Calcd for $C_{16}H_7F_5INO_3$: C, 39.78; H, 1.46; N, 2.90. Found: C, 39.64; H, 1.32; N, 2.83.

2-((1-Iodo-1-phenylpropan-2-yl)oxy)isoindoline-1,3-dione (mixture of diastereomers 1:1) (3ia). White solid: 51% yield (103 mg); mp = 118-119 °C. ^{1}H NMR (300.13 MHz, $CDCl_3$): δ = 7.92-7.66 (m, 4H), 7.60-7.48 (m, 2H), 7.37-7.11 (m, 3H), 5.42 (d, *J* = 7.5 Hz, 0.5H), 5.32 (d, *J* = 6.8 Hz, 0.5H), 4.84-4.68 (m, 1H), 1.69 (d, *J* = 6.1 Hz, 1.5H), 1.32 (d, *J* = 6.3 Hz, 1.5H). ^{13}C NMR (75.47 MHz, $CDCl_3$): δ = 164.1, 163.8, 140.3, 139.9, 134.8, 134.6, 129.13, 129.07, 128.9, 128.74, 128.67, 128.6, 128.50, 128.3, 123.8, 123.6, 87.4, 85.5, 34.3, 32.2, 19.4, 16.8. IR (KBr) ν(cm^{-1}): 1721, 1376, 1189, 1125, 974, 875, 698. HRMS (ESI) Calcd for $C_{17}H_{14}INO_3$ $[M + NH_4]^+$ = 425.0357, Found 425.0348.

2-(2-Iodo-1,2-diphenylethoxy)isoindoline-1,3-dione (mixture of diastereomers 1.8:1) (3ja). White solid: 83% yield (195 mg); mp = 164-165 °C. ^{1}H NMR (300.13 MHz, $CDCl_3$): δ = 7.75-7.53 (m, 6H), 7.41-7.22 (m, 6H), 7.20-7.07 (m, 2H), 6.23 (d, *J* = 10.2 Hz, 0.65H), 5.89 (d, *J* = 9.5 Hz, 0.35 H), 5.64 (d, *J* = 10.2 Hz, 0.65H), 5.49 (d, *J* = 9.5 Hz, 0.35H). ^{13}C NMR (75.47 MHz, $CDCl_3$): δ = 163.4, 163.1, 140.2, 139.9, 136.05, 134.5, 134.35, 134.0, 130.0, 129.4, 129.1, 128.8, 128.71, 128.66, 128.60, 128.55, 128.5, 128.33, 128.29, 128.2, 128.18, 123.5, 123.3, 91.1, 89.8, 31.8, 30.75. IR (KBr) ν(cm^{-1}): 2361, 2343, 1731, 1372, 1188, 1132, 973, 875, 696. Anal. Calcd for $C_{22}H_{16}INO_3$: C, 56.31; H, 3.44; N, 2.98. Found: C, 55.97; H, 3.40; N, 2.95.

2-((1-Iodo-2,3-dihydro-1H-inden-2-yl)oxy)isoindoline-1,3-dione (3ka). White solid: 79% yield (160 mg); mp = 119-120 °C (dec). ^{1}H NMR (300.13 MHz, $CDCl_3$): δ = 7.91-7.76 (m, 4H), 7.52-7.45 (m, 1H), 7.31-7.25 (m, 3H), 5.95 (m, 1H), 5.36 (dt, J_1 = 5.6 Hz, J_2 = 1.4 Hz, 1H), 3.55 (dd, J_1 = 17.6 Hz, J_2 = 5.6 Hz, 1H), 3.33 (d, *J* = 17.6 Hz, 1H). ^{13}C NMR (75.47 MHz, $CDCl_3$): δ = 164.1, 143.0, 139.6, 134.9, 129.2, 128.9, 128.1, 125.9, 125.2, 123.9, 96.2, 36.0, 29.5. ^{15}N NMR (40.56 MHz, $CDCl_3$): δ = -163.9. IR (KBr) ν(cm^{-1}): 1791, 1736, 1463, 1369, 1357, 1186, 1122, 1003, 979, 878, 705. Anal. Calcd for $C_{17}H_{12}INO_3$: C, 50.39; H, 2.99; N, 3.46. Found: C, 50.40; H, 2.97; N, 3.43.

1-(2-Iodo-2-phenylethoxy)pyrrolidine-2,5-dione (3ab). White solid: 67% yield (116 mg); mp = 118-119 °C. ^{1}H NMR (300.13 MHz, $CDCl_3$): δ = 7.50-7.44 (m, 2H), 7.36-7.22 (m, 3H), 5.43 (dd, J_1 = 10.3 Hz, J_2 = 5.2 Hz, 1H), 4.91 (dd, J_1 = 10.6 Hz, J_2 = 10.3 Hz, 1H),

4.56 (dd, J_1 = 10.6 Hz, J_2 = 5.2 Hz, 1H), 2.53-2.34 (m, 4H). ^{13}C NMR (75.47 MHz, $CDCl_3$): δ = 170.8, 140.0, 129.0, 128.7, 127.7, 79.5, 25.4, 25.0. IR (KBr) ν(cm^{-1}): 1722, 1707, 1212, 1077, 943, 811, 698, 651. Anal. Calcd for $C_{12}H_{12}INO_3$: C, 41.76; H, 3.50; N, 4.06. Found: C, 41.73; H, 3.48; N, 4.06.

1-(2-Iodo-2-(p-tolyl)ethoxy)pyrrolidine-2,5-dione (3bb). White solid: 39% yield (70 mg); mp = 126-127 °C (dec). ^{1}H NMR (300.13 MHz, $CDCl_3$): δ = 7.37 (d, J = 8.0 Hz, 2H), 7.13 (d, J = 8.0 Hz, 2H), 5.43 (dd, J_1 = 10.3 Hz, J_2 = 5.3 Hz, 1H), 4.89 (dd, J_1 = 10.6 Hz, J_2 = 10.3 Hz, 1H), 4.54 (dd, J_1 = 10.6 Hz, J_2 = 5.3 Hz, 1H), 2.53-2.41 (m, 4H), 2.31 (s, 3H). ^{13}C NMR (75.47 MHz, $CDCl_3$): δ = 170.8, 138.8, 137.0, 129.7, 127.6, 79.5, 25.4, 21.4. IR (KBr) ν(cm^{-1}): 1724, 1707, 1214, 1085, 997, 811, 657, 562. Anal. Calcd for $C_{13}H_{14}INO_3$: C, 43.47; H, 3.93; N, 3.90. Found: C, 43.55; H, 3.95; N, 3.88.

1-(2-(4-Chlorophenyl)-2-iodoethoxy)pyrrolidine-2,5-dione (3cb). White solid: 61% yield (116 mg); mp = 117-118 °C. ^{1}H NMR (300.13 MHz, $CDCl_3$): δ = 7.46 (d, J = 8.5 Hz, 2H), 7.32 (d, J = 8.5 Hz, 2H), 5.42 (dd, J_1 = 10.3 Hz, J_2 = 5.3 Hz, 1H), 4.84 (dd, J_1 = 10.6 Hz, J_2 = 10.3 Hz, 1H), 4.54 (dd, J_1 = 10.6 Hz, J_2 = 5.3 Hz, 1H), 2.61-2.54 (m, 4H). ^{13}C NMR (75.47 MHz, $CDCl_3$): δ = 170.7, 138.5, 134.4, 129.2, 129.16, 79.7, 25.5, 23.4. IR (KBr) ν(cm^{-1}): 1709, 1492, 1194, 1092, 1080, 996, 835, 649. HRMS (ESI) Calcd for $C_{12}H_{11}ClINO_3$ $[M + Na]^+$ = 401.9364, Found 401.9364.

1-(2-(4-Fluorophenyl)-2-iodoethoxy)pyrrolidine-2,5-dione (3db). White solid: 63% yield (114 mg); mp = 105-106 °C. ^{1}H NMR (300.13 MHz, $CDCl_3$): δ = 7.52-7.45 (m, 2H), 7.07-6.97 (m, 2H), 5.43 (dd, J_1 = 10.2 Hz, J_2 = 5.3 Hz, 1H), 4.82 (dd, J_1 = 10.6 Hz, J_2 = 10.2 Hz, 1H), 4.53 (dd, J_1 = 10.6 Hz, J_2 = 5.3 Hz, 1H), 2.61-2.52 (m, 4H). ^{13}C NMR (75.47 MHz, $CDCl_3$): δ = 170.7, 162.5 (d, J = 249.0 Hz), 135.8 (d, J = 3.4 Hz), 129.7 (d, J = 8.4 Hz), 116.0 (d, J = 21.9 Hz), 79.9, 25.45, 23.8. ^{19}F NMR (282.40 MHz, $CDCl_3$): δ = -112.2 (1F). IR (KBr) ν(cm^{-1}): 1726, 1707, 1601, 1509, 1208, 1082, 840, 812, 652. Anal. Calcd for $C_{12}H_{11}FINO_3$: C, 39.69; H, 3.05; N, 3.86. Found: C, 39.70; H, 3.03; N, 3.86.

1-(2-(3-Bromophenyl)-2-iodoethoxy)pyrrolidine-2,5-dione (3fb). White solid: 34% yield (72 mg); mp = 184-185 °C. ^{1}H NMR (300.13 MHz, DMSO-d_6): δ = 7.76 (t, J = 1.7 Hz, 1H), 7.57-7.51 (m, 1H), 7.49-7.44 (m, 1H), 7.30 (t, J = 7.8 Hz, 1H), 5.54 (dd, J_1 = 9.8 Hz, J_2 = 5.7 Hz, 1H), 4.72 (dd, J_1 = 11.0 Hz, J_2 = 9.8 Hz, 1H), 4.48 (dd, J_1 = 11.0 Hz, J_2 = 5.7

Hz, 1H), 2.59-2.53 (m, 4H). ^{13}C NMR (75.47 MHz, DMSO-d$_6$): δ = 171.7, 143.2, 130.9, 130.7, 130.4, 127.1, 121.4, 78.6, 25.4, 24.5. IR (KBr) ν(cm^{-1}): 1725, 1707, 1211, 1202, 1083, 809, 789, 693, 670, 660. Anal. Calcd for $C_{12}H_{11}BrINO_3$: C, 33.99; H, 2.61; N, 3.30. Found: C, 34.02; H, 2.69; N, 3.26.

1-(2-Iodo-2-(perfluorophenyl)ethoxy)pyrrolidine-2,5-dione (3hb). White solid: 60% yield (130 mg); mp = 110-111 °C. ^{1}H NMR (300.13 MHz, CDCl$_3$): δ = 5.56 (dd, J_1 = 10.7 Hz, J_2 = 5.8 Hz, 1H), 4.81 (t, J = 10.7 Hz, 1H), 4.59 (dd, J_1 = 10.7 Hz, J_2 = 5.8 Hz, 1H), 2.76-2.64 (m, 4H). ^{13}C NMR (75.47 MHz, CDCl$_3$): δ = 170.6, 146.5-146.2 (m), 144.5-144.2 (m), 142.7-142.4 (m), 140.7-140.4 (m), 139.2-138.8 (m), 137.1-136.8 (m) 113.9 (td, J_1 = 14.4 Hz, J_2 = 4.2 Hz), 78.8 (t, J = 3.8 Hz), 25.4, 5.9. ^{19}F NMR (282.40 MHz, CDCl$_3$): δ = -140.2 (bs, 2F), -152.9 (t, J = 21.0 Hz, 1F) -161.2 (td, J_1 = 21.0 Hz, J_2 = 6.4 Hz, 2F). IR (KBr) ν(cm^{-1}): 1783, 1727, 1526, 1503, 1365, 1195, 1138, 1123, 1073, 1062, 1040, 1000, 969, 930, 902, 815, 750, 704, 687, 665. Anal. Calcd for $C_{12}H_7F_5INO_3$: C, 33.13; H, 1.62; N, 3.22. Found: C, 33.24; H, 1.61; N, 3.32.

1-((1-Iodo-2,3-dihydro-1H-inden-2-yl)oxy)pyrrolidine-2,5-dione (3kb). White solid: 60% yield (107 mg); mp = 147-148 °C (dec). ^{1}H NMR (300.13 MHz, CDCl$_3$): δ = 7.46-7.41 (m, 1H), 7.32-7.22 (m, 3H), 5.87 (m, 1H), 5.29 (dt, J_1 = 5.8 Hz, J_2 = 1.5 Hz, 1H), 3.50 (dd, J_1 = 17.5 Hz, J_2 = 5.8 Hz, 1H), 3.34-3.20 (m, 1H), 2.74-2.65 (m, 4H). ^{13}C NMR (75.47 MHz, CDCl$_3$): δ = 171.5, 142.7, 139.3, 129.2, 128.1, 125.9, 125.1, 95.4, 36.0, 29.7, 25.5. IR (KBr) ν(cm^{-1}): 1730, 1194, 1066, 996, 719, 648 Anal. Calcd for $C_{13}H_{12}INO_3$: C, 43.72; H, 3.39; N, 3.92. Found: C, 43.71; H, 3.21; N, 3.93.

Gram-scale synthesis of compound 3aa (experimental for Scheme 4)

Iodine (1.27 g, 5 mmol) was added to a stirred mixture of styrene **1a** (1.04 g, 10 mmol) and *N*-hydroxyphthalimide (**2a**, 1.63 g, 10 mmol) in CH_2Cl_2 (50 mL) at 20–25 °C. Then, $PhI(OAc)_2$ (1.94 g, 6 mmol) was added. After stirring the reaction mixture under air atmosphere at 20–25 °C for 10 minutes the mixture was washed with aqueous solution of $Na_2S_2O_3{\cdot}5H_2O$ (300 mg in 50 mL of water), saturated aqueous $NaHCO_3$ solution (50 mL), then with water (50 mL), dried over anhydrous $MgSO_4$ and filtered. DCM was rotary evaporated at 20–25 °C under water-jet vacuum (20–30 mmHg). Crude solid product was washed with 2 × 25 mL ice-cold hexane and then recrystallized from DCM – hexane mixture (50 mL, 1:1 v/v) at 30–35 °C. Yield of **3aa** – 3.1 g (79%), mp = 136–137

°C. Anal. Calcd for $C_{16}H_{12}INO_3$: C, 48.88; H, 3.08; N, 3.56. Found: C, 48.88; H, 3.05; N, 3.56.

Synthesis of compound 4a

To a stirred solution of **3aa** (197 mg, 0.5 mmol) in CH_2Cl_2/MeOH (1/1 v/v, 5.0 mL) *m*CPBA (148 mg, 0.6 mmol) was added, and the reaction mixture was vigorously stirred at 20–25 °C for 24 h. Then CH_2Cl_2 (20 mL) was added and the reaction mixture was washed with aqueous solution of $Na_2S_2O_3 \cdot 5H_2O$ (200 mg in 20 mL of water), water (20 mL), dried over anhydrous $MgSO_4$, the solvent was rotary evaporated at 45–50 °C under water-jet vacuum (20–30 mmHg). Compound **4a** was analytically pure and no further purification was performed.

2-(2-Methoxy-2-phenylethoxy)isoindoline-1,3-dione (4a). White solid: 87% yield (129 mg); mp = 127-128 °C. ^{1}H NMR (300.13 MHz, $CDCl_3$): δ = 7.90-7.69 (m, 4H), 7.46-7.29 (m, 5H), 4.71 (dd, J_1 = 2.6 Hz, J_2 = 8.8 Hz, 1H), 4.42 (dd, J_1 = 8.8 Hz, J_2 = 11.5 Hz, 1H), 4.12 (dd, J_1 = 2.6 Hz, J_2 = 11.5 Hz, 1H), 3.25 (s, 3H). ^{13}C NMR (75.47 MHz, $CDCl_3$): δ = 163.5, 137.5, 134.5, 129.2, 128.8, 128.6, 127.1, 123.6, 82.3, 81.2, 57.1. IR (KBr) ν(cm^{-1}): 1785, 1731, 1464, 1451, 1376, 1186, 1138, 1083, 991, 764, 701. HRMS (ESI) Calcd for $C_{17}H_{15}NO_4$ $[M + NH_4]^+$ = 315.1339, Found 315.1352.

Synthesis of compound 4b

To a stirred solution of **3aa** (197 mg, 0.50 mmol) in DMF (3.0 mL) sodium benzenesulfinate (820 mg, 5.0 mmol) was added, and the reaction mixture was vigorously stirred at 60 °C for 6 h. Upon completion of the reaction water (20 mL) was added and reaction mixture was extracted with an EtOAc/hexane mixture (1:1 v/v) (2 × 10 mL), the combined extracts were washed with water (3 × 20 mL), dried over anhydrous $MgSO_4$, the solvent was rotary evaporated at 45–50 °C under water-jet vacuum (20-30 mmHg). Compound **4b** was isolated by column chromatography on silica gel using EtOAc – DCM eluent (5% volume part of EtOAc).

(1-Phenylethane-1,2-diyldisulfonyl)dibenzene (4b). White solid: 69% yield (134 mg); mp = 179-180 °C (lit. 179 °C) [4]. ^{1}H NMR (300.13 MHz, $CDCl_3$): δ = 7.61-7.24 (m, 10H), 7.18 (t, J = 7.4 Hz, 1H), 7.04 (t, J = 7.6 Hz, 2H), 6.91 (d, J = 7.6 Hz, 2H), 4.62 (dd, J_1 = 11.8 Hz, J_2 = 2.3 Hz, 1H), 4.18 (dd, J_1 = 14.4 Hz, J_2 = 2.3 Hz, 1H), 3.99 (dd, J_1 = 14.4 Hz, J_2 = 11.8 Hz, 1H). ^{13}C NMR (75.47 MHz, $CDCl_3$): 139.2, 136.0, 134.3, 133.8,

130.0, 129.4, 129.3, 129.25, 129.2, 128.05, 128.6, 128.0, 66.5, 53.9. IR (KBr) ν(cm^{-1}): 1308, 1146, 1080, 750, 727, 696, 685, 538, 528. Anal. Calcd for $C_{20}H_{18}S_2O_4$: C, 62.16; H, 4.69. Found: C, 62.17; H, 4.75. HRMS (ESI) Calcd for $C_{20}H_{18}S_2O_4$ $[M + Na]^+$ = 409.0539, Found 409.0544.

Synthesis of compound 4c

To a stirred solution of **3ab** (173 mg, 0.50 mmol) in DMF (3.0 mL) NaN_3 (325 mg, 5.0 mmol) was added, and the reaction mixture was vigorously stirred at 20–25 °C for 12 h. Then water (20 mL) was added and reaction mixture was extracted with of EtOAc/hexane mixture (1:1 v/v) (2 × 10 mL), combined extracts were washed with water (3 × 20 mL), dried over anhydrous $MgSO_4$, solvent was rotary evaporated at 45–50 °C under water-jet vacuum (20–30 mmHg). Compound **4c** was analytically pure and no further purification was performed.

1-(2-azido-2-phenylethoxy)pyrrolidine-2,5-dione (4c). White solid: 86% yield (142 mg); mp = 106-107 °C. ^{1}H NMR (300.13 MHz, $CDCl_3$): δ = 7.42-7.29 (m, 5H), 4.95 (dd, J_1 = 8.2 Hz, J_2 = 4.5 Hz, 1H), 4.30-4.19 (m, 2H), 2.68 (s, 4H). ^{13}C NMR (75.47 MHz, $CDCl_3$): δ = 170.9, 135.2, 129.2, 127.3, 79.3, 64.2, 25.5. IR (KBr) ν(cm^{-1}): 2118, 2092, 2077, 1722, 1709, 1320, 1253, 1234, 1214, 1085, 767, 709, 649. HRMS (ESI) Calcd for $C_{12}H_{12}N_4O_3$ $[M + Na]^+$ = 283.0802, Found 283.0792.

Procedure for the electrochemical studies

Cyclic voltammetry (CV) was implemented on an IPC-Pro computer-assisted potentiostat manufactured by Econix (scan rate error 1.0%; potential setting 0.25 mV). The experiments were performed in a 10 mL five-neck glass conic electrochemical cell with a water jacket for thermostatting. CV curves were recorded using a three-electrode scheme. The working electrode was a disc glassy-carbon electrode (d = 1.7 mm). A platinum wire served as an auxiliary electrode. A Ag^+/AgCl electrode was used as the reference electrode and was linked to the solution by a bridge with a porous ceramic diaphragm filled with background electrolyte. The tested solutions were thermostatted at 25 +/− 0.5 °C. In a typical case, 5 mL of solution was utilized. The compound concentration was 2 mmol·L^{-1} (I_2 and $PhI(OAc)_2$) or 4 mmol·L^{-1} (NHPI and styrene). The working electrode was polished before recording each CV curve.

References

1. *Org. Synth.* **1988**, *66*. doi: 10.15227/orgsyn.066.0132

2. Frigerio, M.; Santagostino, M.; Sputore, S. *J. Org. Chem.* **1999**, *64*, 4537. doi: 10.1021/jo9824596

3. *Org. Synth.* **2000**, *77*. doi: 10.15227/orgsyn.077.0141

4. Redman, R. P.; Thomas, P. J.; Stirling, C. J. M. *J. Chem. Soc., Perkin Trans. 2* **1978**, *11*, 1135. doi: 10.1039/P29780001135

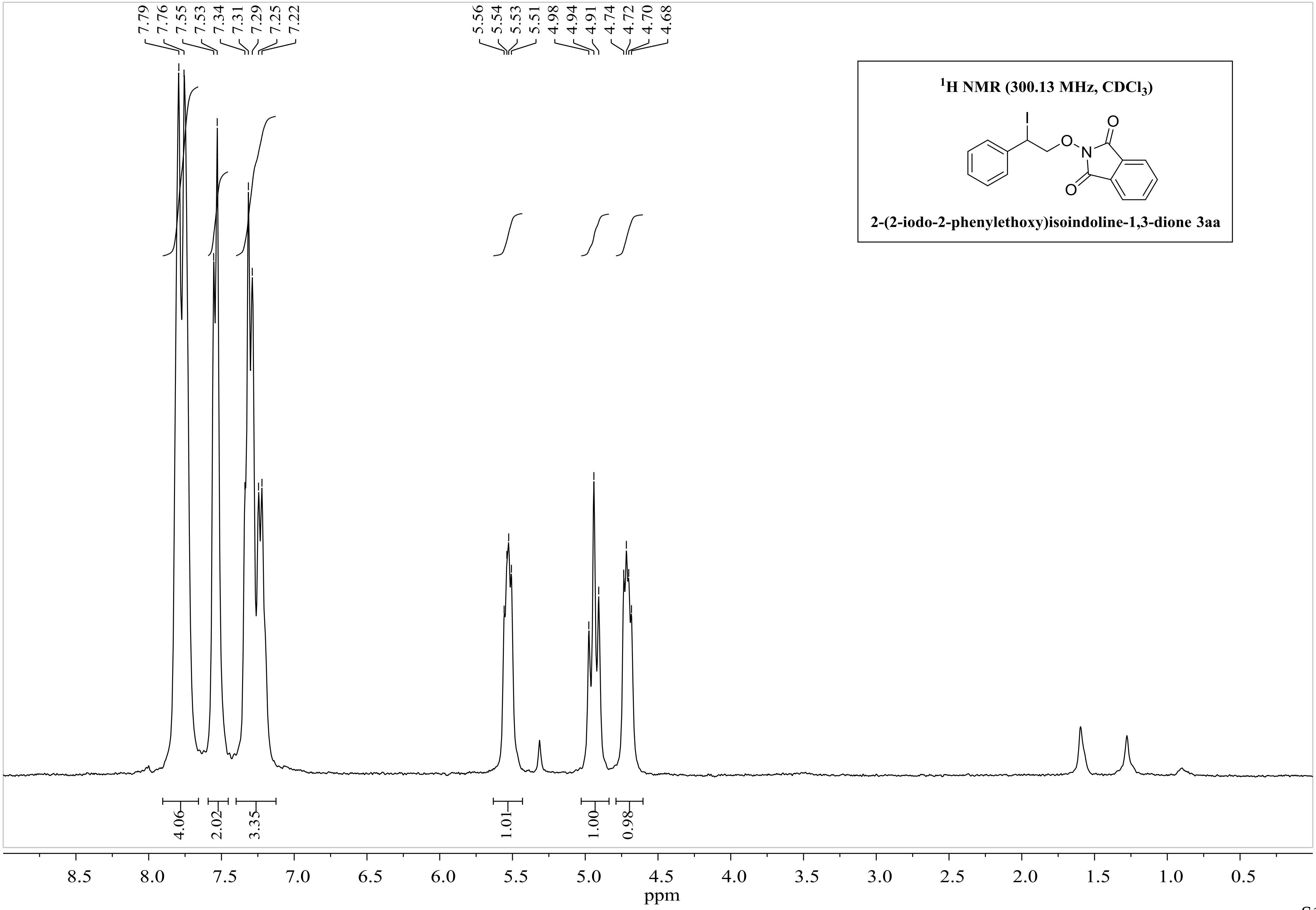

7.79
7.76
7.55
7.53
7.34
7.31
7.29
7.25
7.22
5.56
5.54
5.53
5.51
4.98
4.94
4.91
4.74
4.72
4.70
4.68
^{1}H NMR (300.13 MHz, $CDCl_3$)
I
O
N
O
O
2-(2-iodo-2-phenylethoxy)isoindoline-1,3-dione 3aa
4.06
2.02
3.35
1.01
1.00
0.98
8.5
8.0
7.5
7.0
6.5
6.0
5.5
5.0
4.5
4.0
3.5
3.0
2.5
2.0
1.5
1.0
0.5
ppm

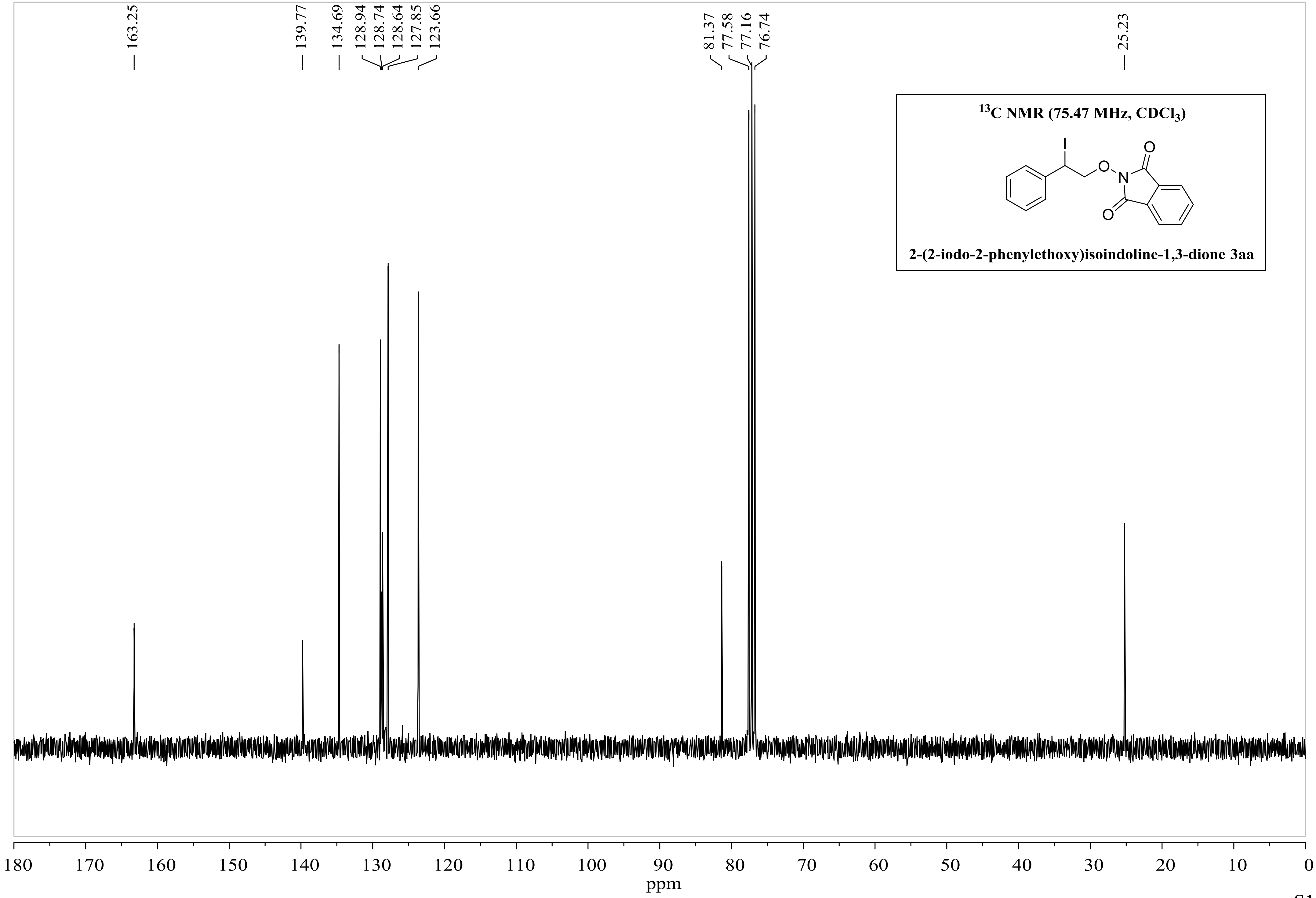
163.25
139.77
134.69
128.94
128.74
128.64
127.85
123.66
81.37
77.58
77.16
76.74
25.23
^{13}C NMR (75.47 MHz, $CDCl_3$)
2-(2-iodo-2-phenylethoxy)isoindoline-1,3-dione 3aa
180 170 160 150 140 130 120 110 100 90 80 70 60 50 40 30 20 10 0
ppm

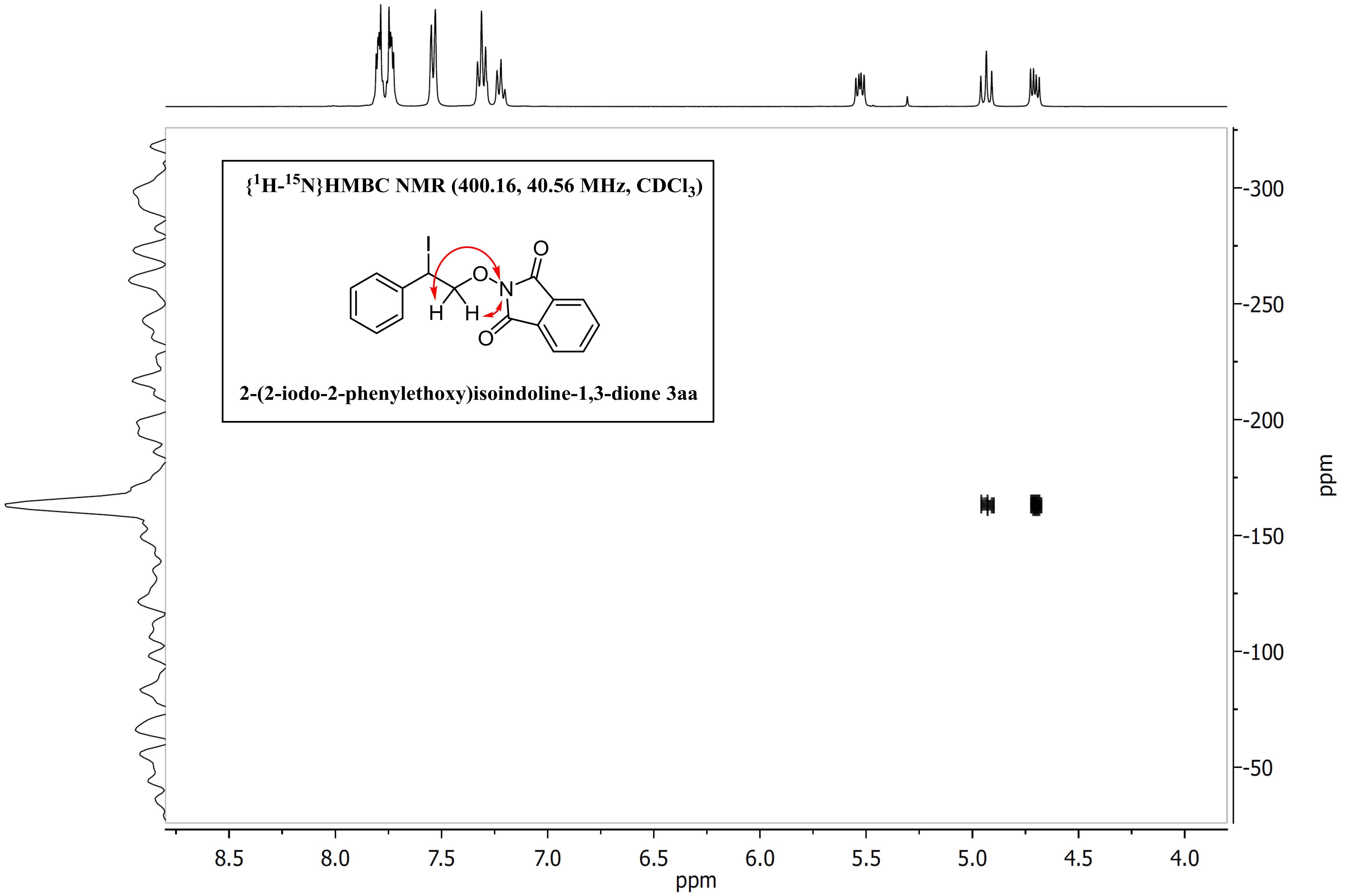
{1H-15N}HMBC NMR (400.16, 40.56 MHz, CDCl3)
I
O
N
O
O
H
H
2-(2-iodo-2-phenylethoxy)isoindoline-1,3-dione 3aa
-300
-250
-200
-150
-100
-50
ppm
8.5
8.0
7.5
7.0
6.5
6.0
5.5
5.0
4.5
4.0
ppm

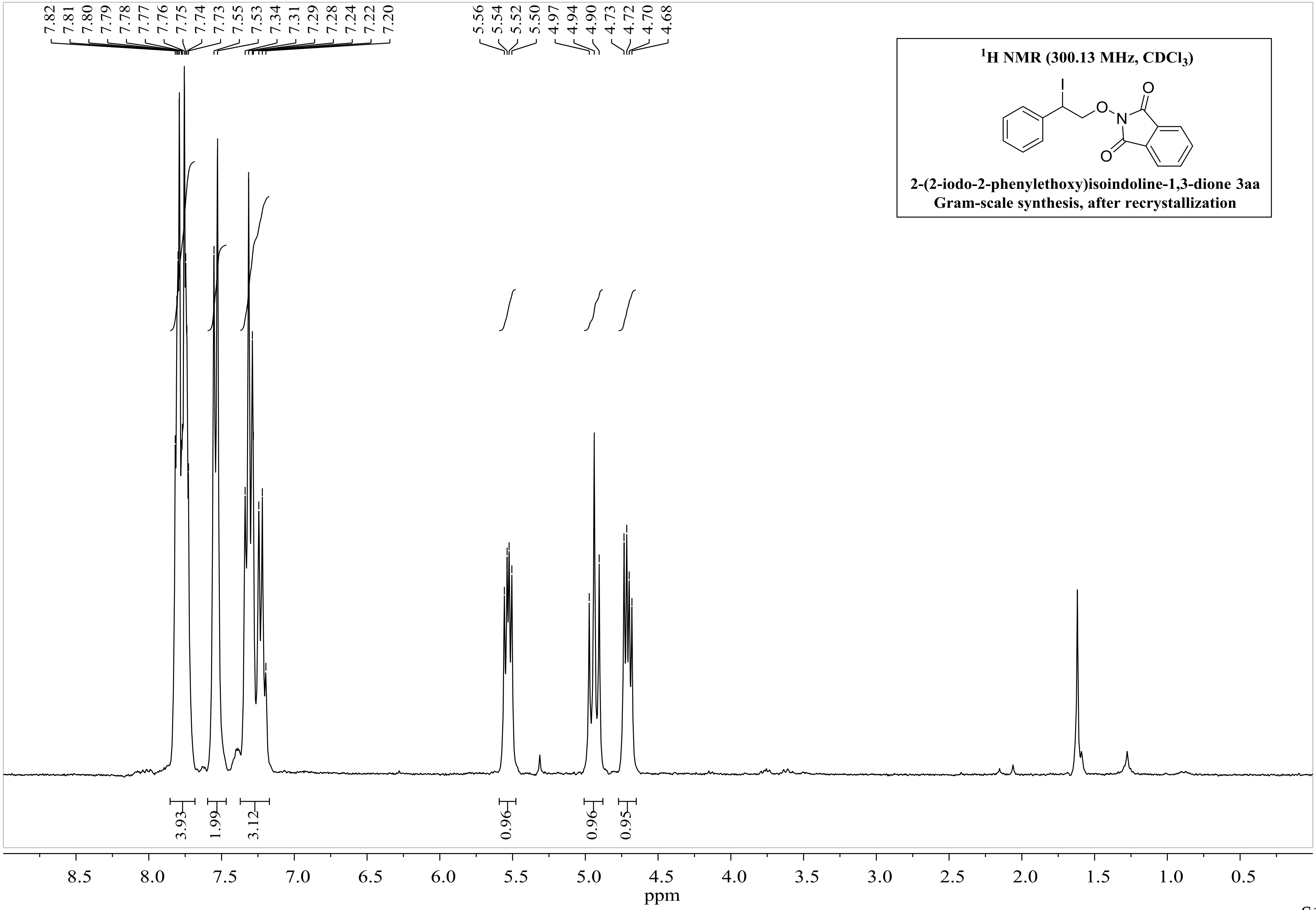
7.82
7.81
7.80
7.79
7.78
7.77
7.76
7.75
7.74
7.73
7.55
7.53
7.34
7.31
7.29
7.28
7.24
7.22
7.20
5.56
5.54
5.52
5.50
4.97
4.94
4.90
4.73
4.72
4.70
4.68
^{1}H NMR (300.13 MHz, $CDCl_3$)
I
O
O
N
O
2-(2-iodo-2-phenylethoxy)isoindoline-1,3-dione 3aa
Gram-scale synthesis, after recrystallization
3.93
1.99
3.12
0.96
0.96
0.95
8.5
8.0
7.5
7.0
6.5
6.0
5.5
5.0
4.5
4.0
3.5
3.0
2.5
2.0
1.5
1.0
0.5
ppm

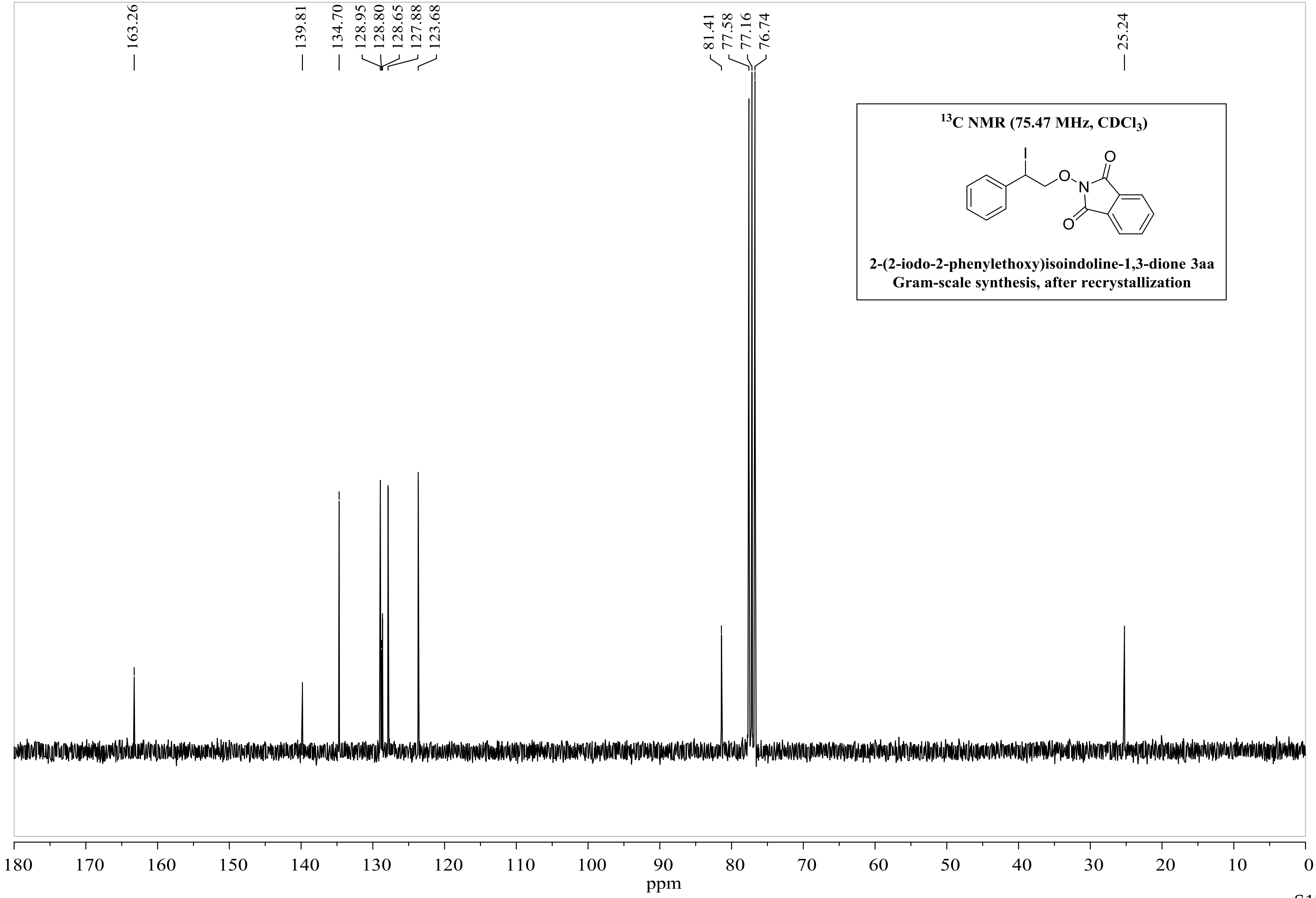
163.26
139.81
134.70
128.95
128.80
128.65
127.88
123.68
81.41
77.58
77.16
76.74
25.24
^{13}C NMR (75.47 MHz, $CDCl_3$)
I
O
O
N
O
2-(2-iodo-2-phenylethoxy)isoindoline-1,3-dione 3aa
Gram-scale synthesis, after recrystallization
180
170
160
150
140
130
120
110
100
90
80
70
60
50
40
30
20
10
0
ppm

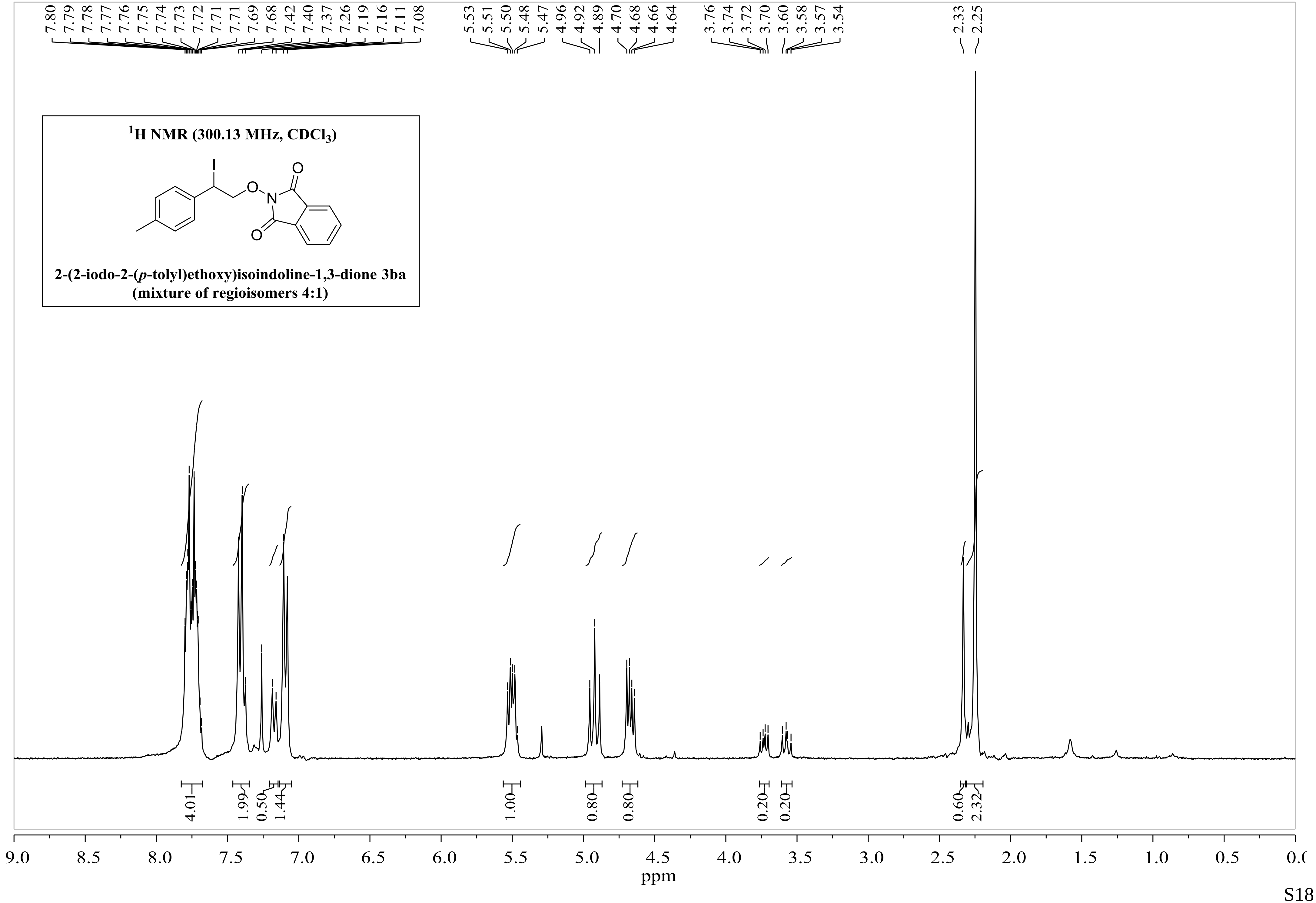
7.80 7.79 7.78 7.77 7.76 7.75 7.74 7.73 7.72 7.71 7.71 7.69 7.68 7.42 7.40 7.37 7.26 7.19 7.16 7.11 7.08
5.53 5.51 5.50 5.48 5.47 4.96 4.92 4.89 4.70 4.68 4.66 4.64
3.76 3.74 3.72 3.70 3.60 3.58 3.57 3.54
2.33 2.25
1H NMR (300.13 MHz, CDCl3)
I
O
N
O
O
2-(2-iodo-2-(p-tolyl)ethoxy)isoindoline-1,3-dione 3ba
(mixture of regioisomers 4:1)
4.01 1.99 0.50 1.44 1.00 0.80 0.80 0.20 0.20 0.60 2.32
9.0 8.5 8.0 7.5 7.0 6.5 6.0 5.5 5.0 4.5 4.0 3.5 3.0 2.5 2.0 1.5 1.0 0.5 0.0
ppm

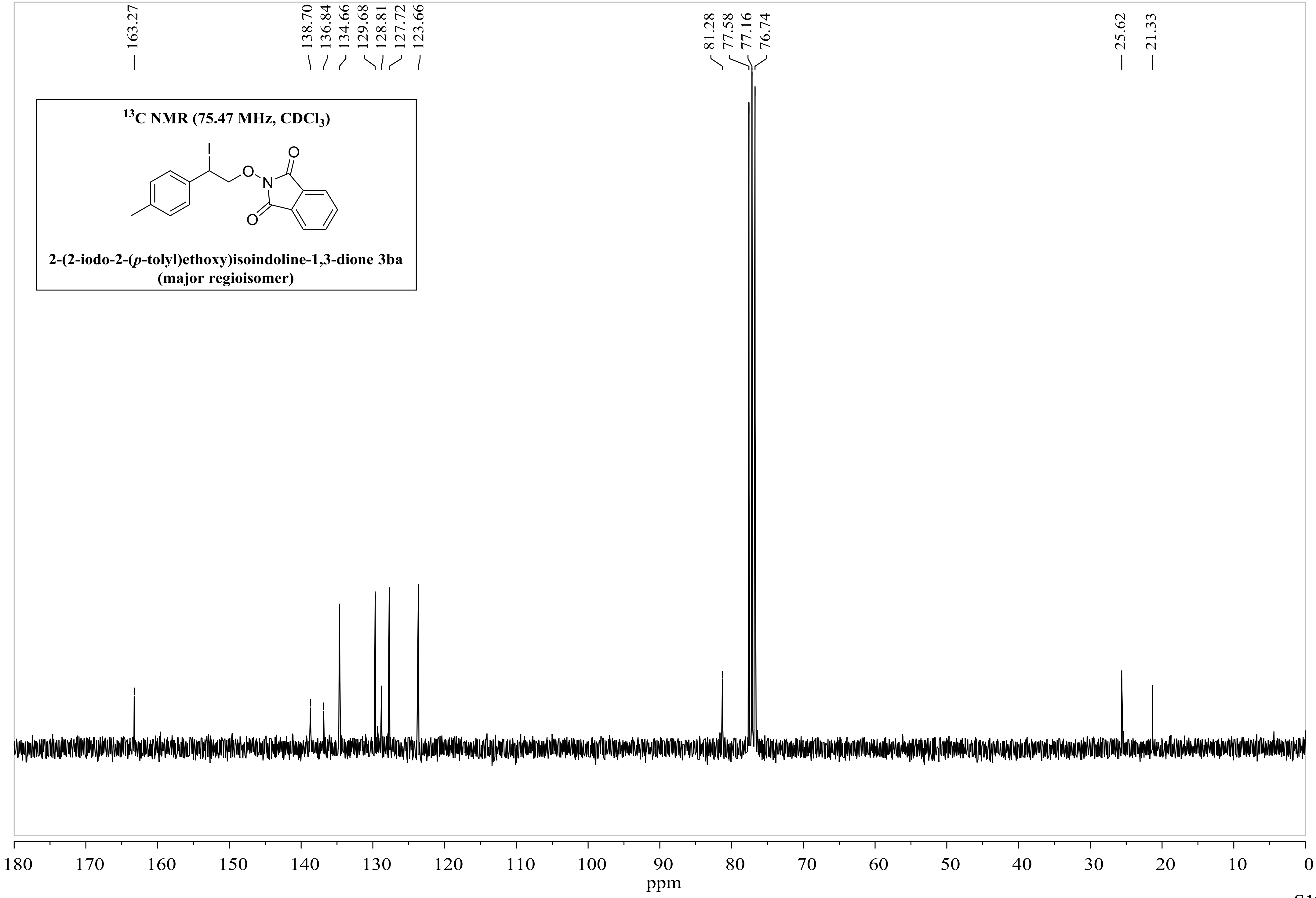
163.27
138.70
136.84
134.66
129.68
128.81
127.72
123.66
81.28
77.58
77.16
76.74
25.62
21.33
^{13}C NMR (75.47 MHz, $CDCl_3$)
I
O
O
N
O
2-(2-iodo-2-(*p*-tolyl)ethoxy)isoindoline-1,3-dione 3ba
(major regioisomer)
180
170
160
150
140
130
120
110
100
90
80
70
60
50
40
30
20
10
0
ppm

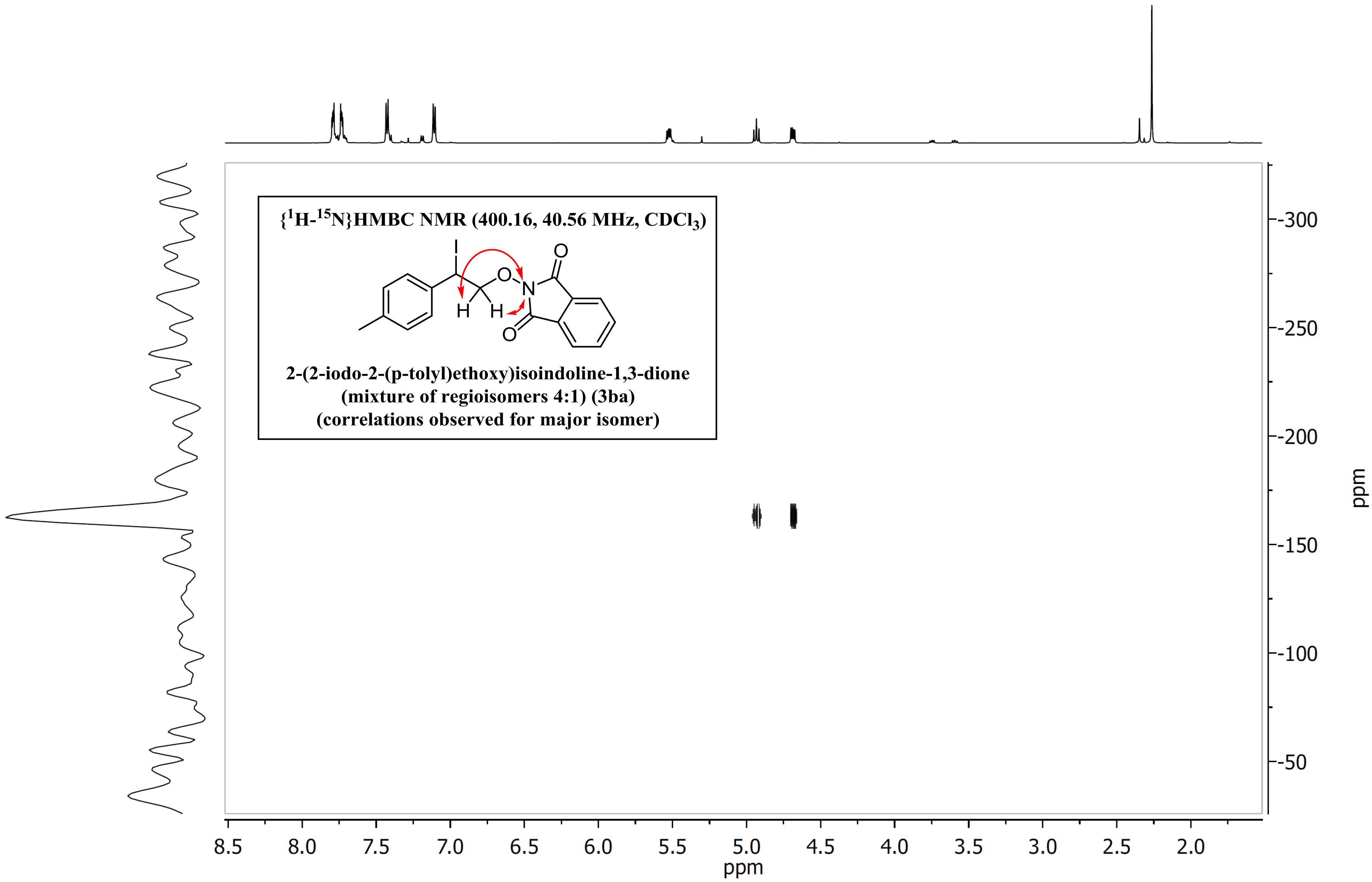
{1H-15N}HMBC NMR (400.16, 40.56 MHz, CDCl3)
2-(2-iodo-2-(p-tolyl)ethoxy)isoindoline-1,3-dione
(mixture of regioisomers 4:1) (3ba)
(correlations observed for major isomer)
ppm
ppm

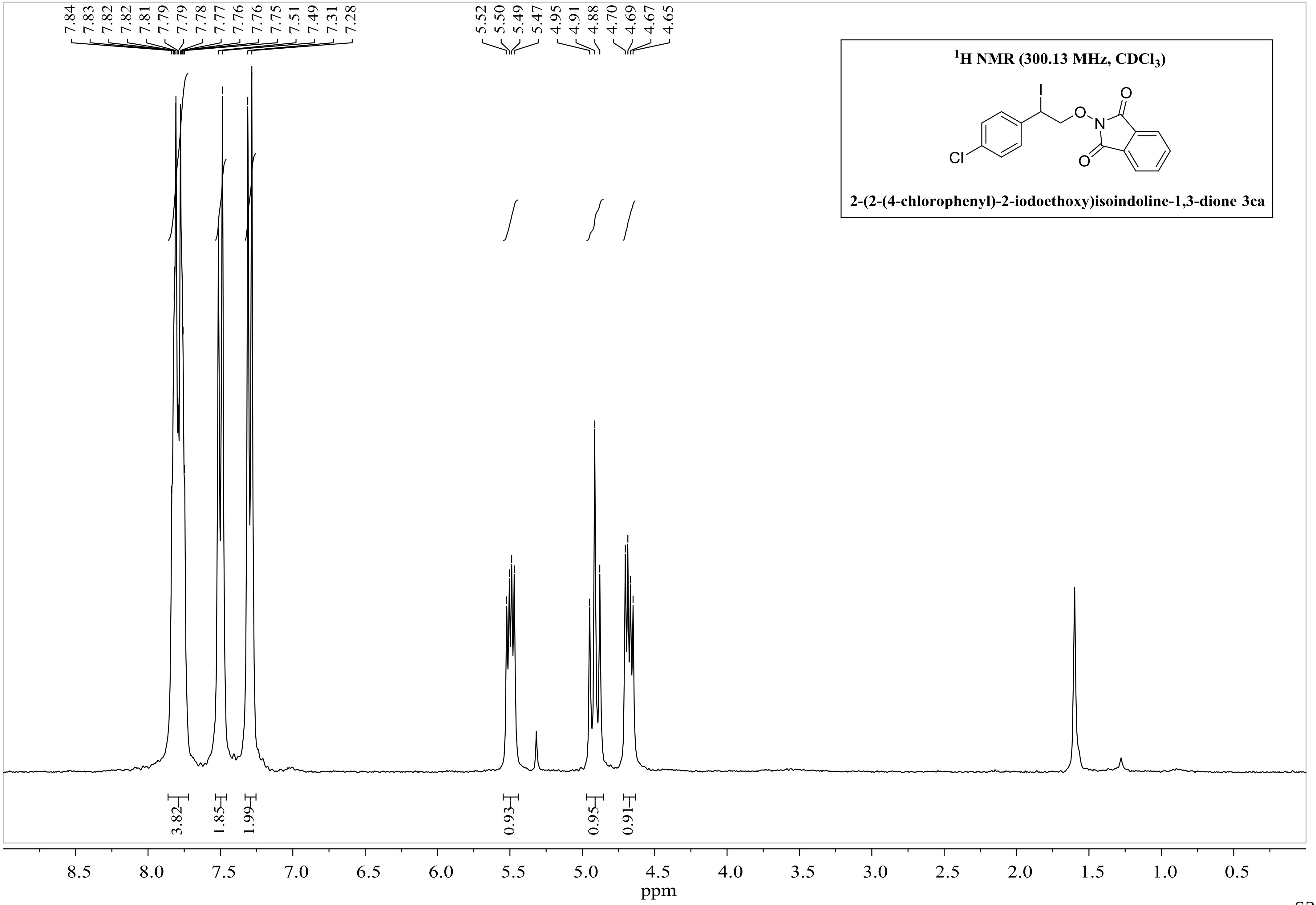

^{1}H NMR (300.13 MHz, CDCl$_3$)
2-(2-(4-chlorophenyl)-2-iodoethoxy)isoindoline-1,3-dione 3ca
7.84 7.83 7.82 7.82 7.81 7.79 7.79 7.78 7.77 7.76 7.76 7.75 7.51 7.49 7.31 7.28
5.52 5.50 5.49 5.47 4.95 4.91 4.88 4.70 4.69 4.67 4.65
3.82 1.85 1.99 0.93 0.95 0.91
8.5 8.0 7.5 7.0 6.5 6.0 5.5 5.0 4.5 4.0 3.5 3.0 2.5 2.0 1.5 1.0 0.5
ppm

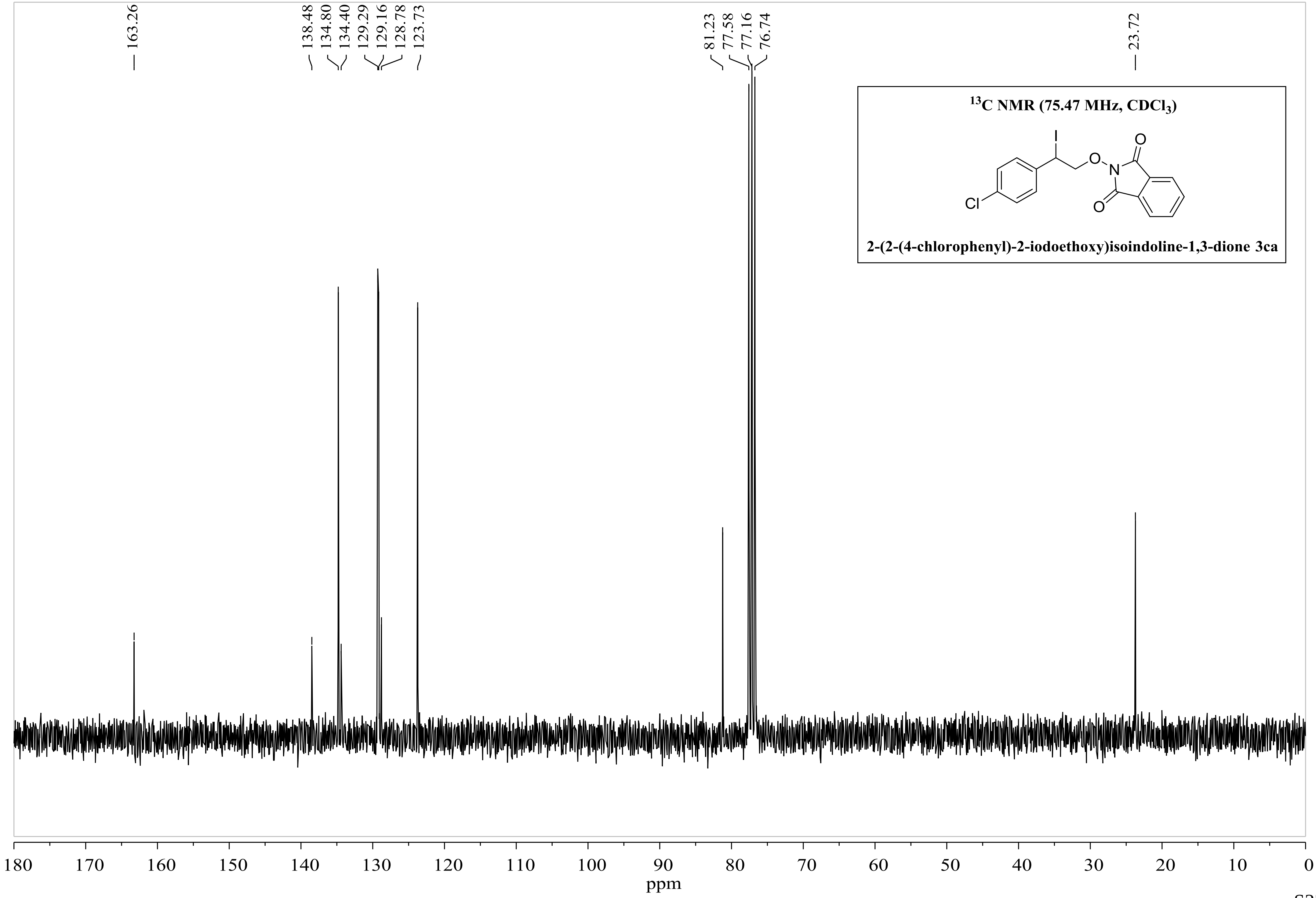
163.26
138.48
134.80
134.40
129.29
129.16
128.78
123.73
81.23
77.58
77.16
76.74
23.72
13C NMR (75.47 MHz, CDCl3)
I
O
O
N
O
Cl
O
2-(2-(4-chlorophenyl)-2-iodoethoxy)isoindoline-1,3-dione 3ca
180
170
160
150
140
130
120
110
100
90
80
70
60
50
40
30
20
10
0
ppm

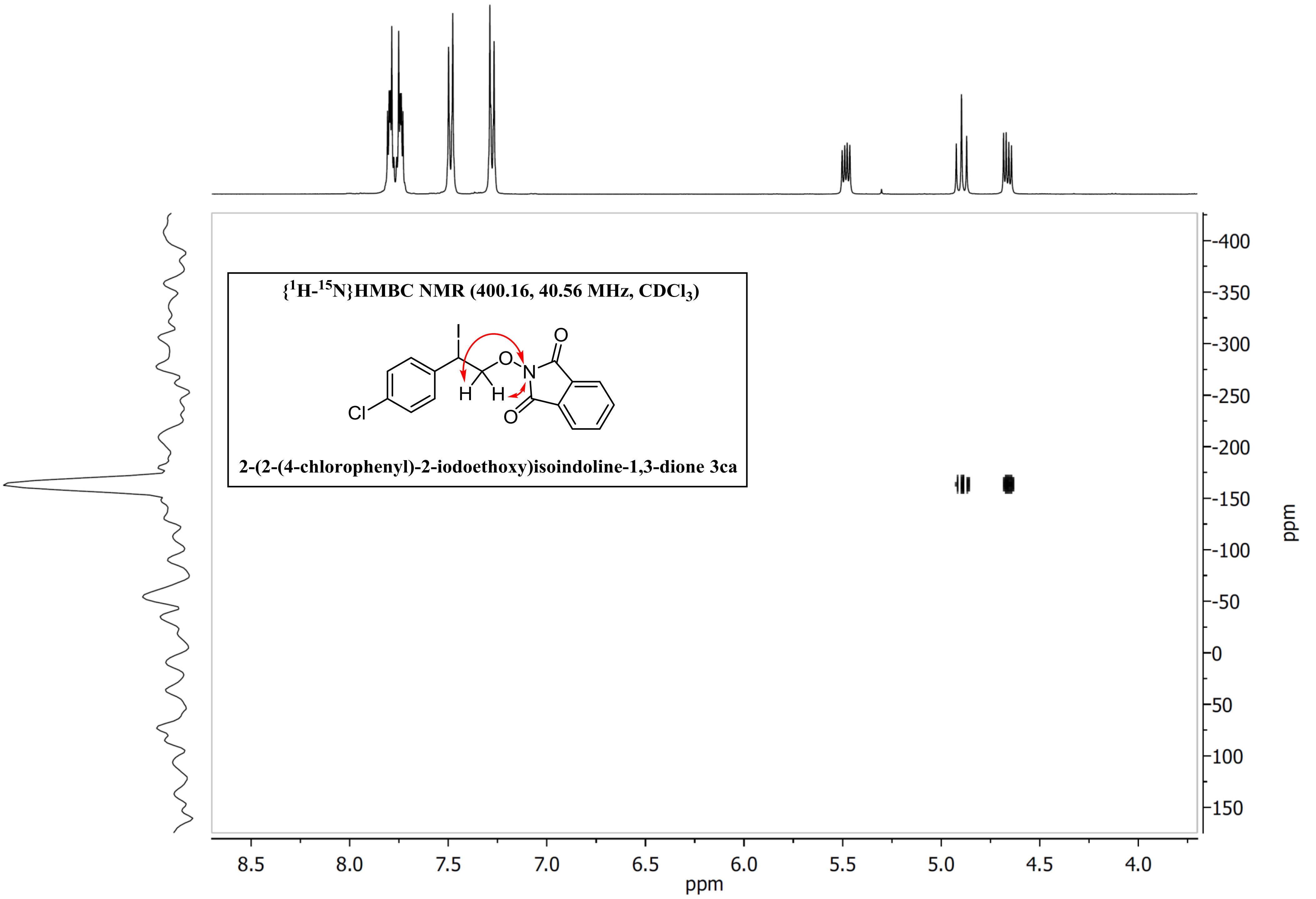
{1H-15N}HMBC NMR (400.16, 40.56 MHz, CDCl3)
I
O
O
N
H
H
Cl
O
2-(2-(4-chlorophenyl)-2-iodoethoxy)isoindoline-1,3-dione 3ca
-400
-350
-300
-250
-200
-150
-100
-50
0
50
100
150
ppm
8.5
8.0
7.5
7.0
6.5
6.0
5.5
5.0
4.5
4.0
ppm

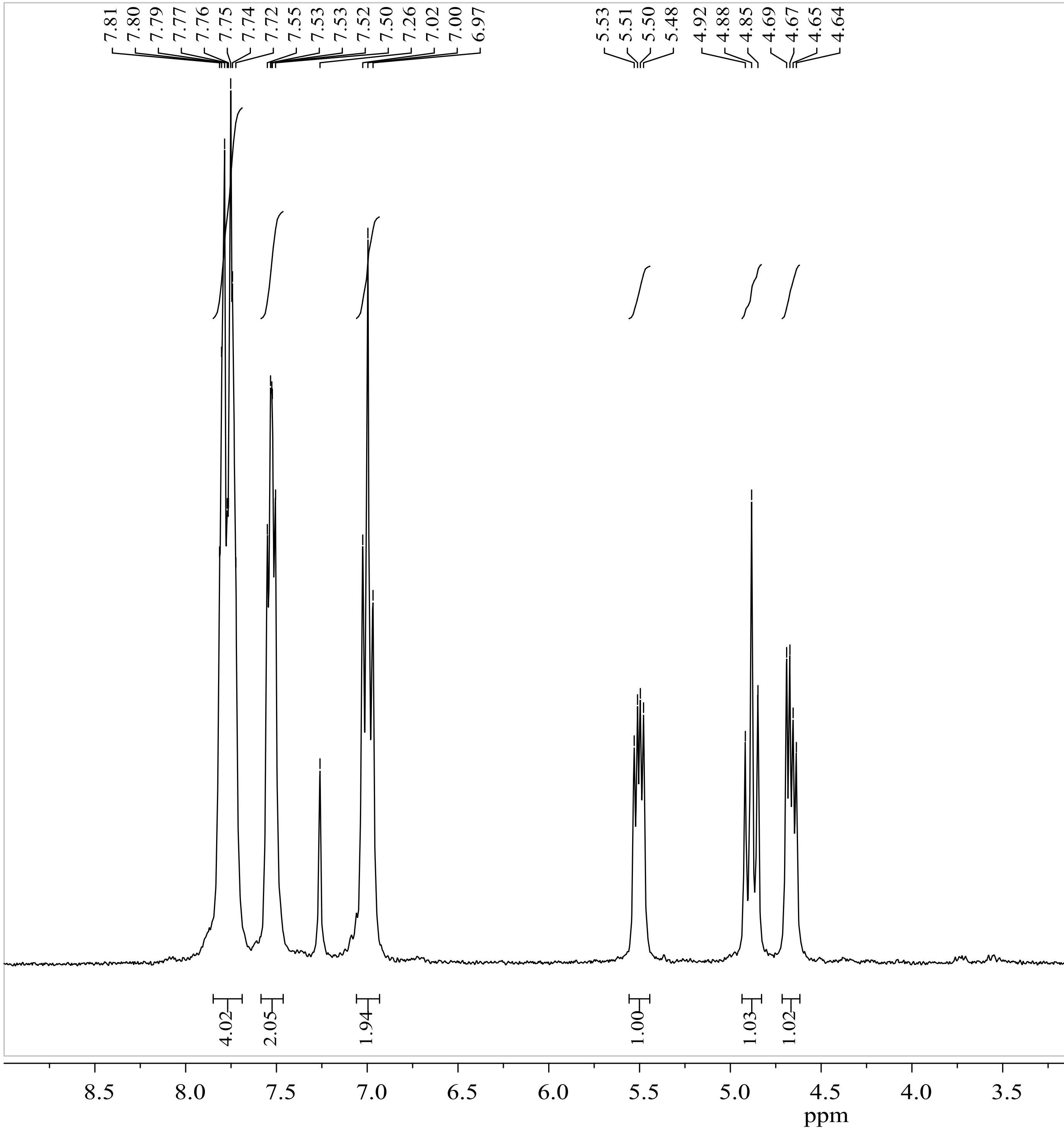

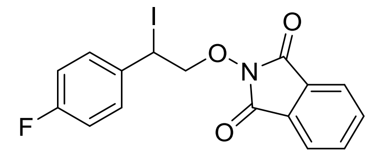

2-(2-(4-fluorophenyl)-2-iodoethoxy)isoindoline-1,3-dione 3da

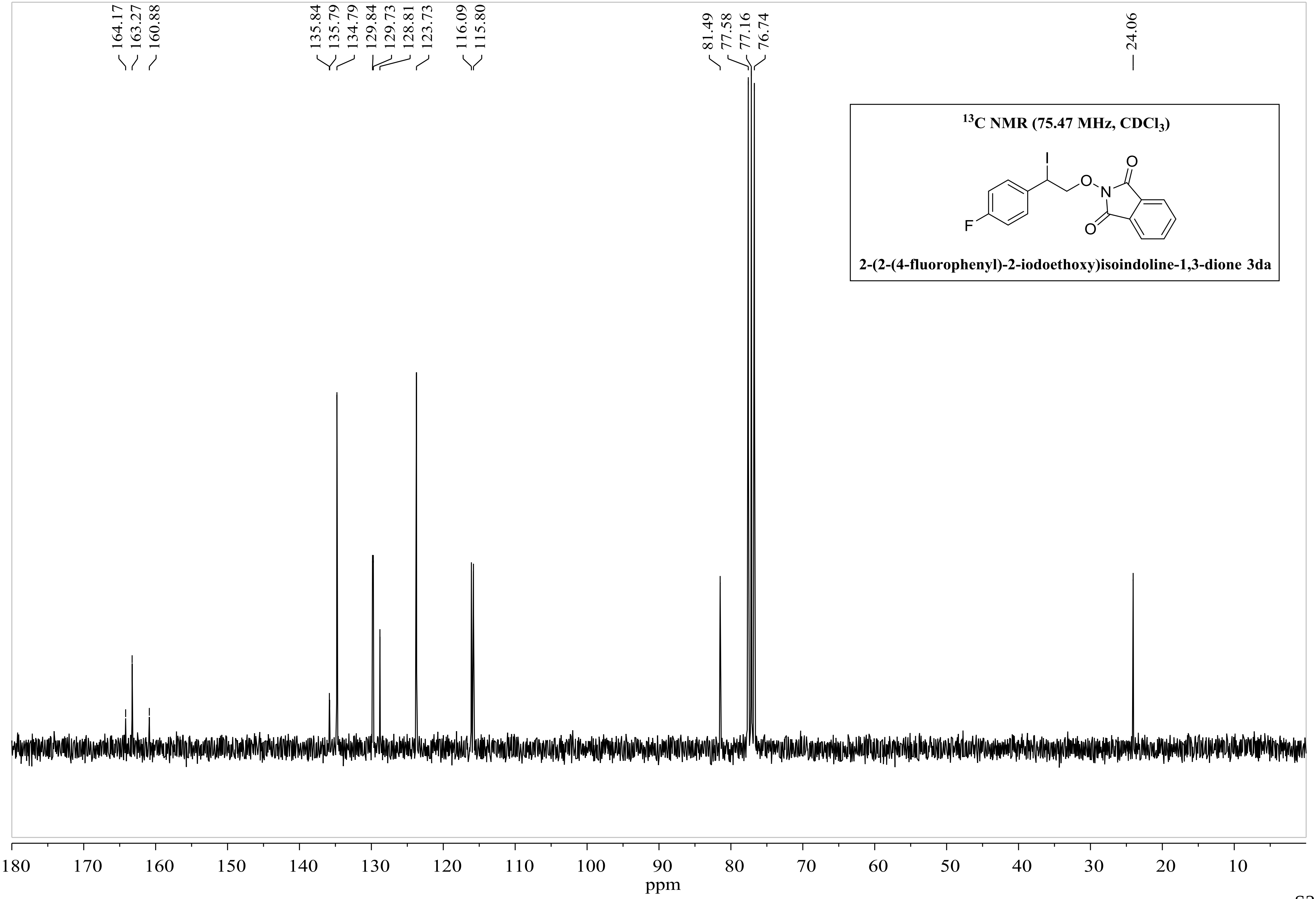
164.17
163.27
160.88
135.84
135.79
134.79
129.84
129.73
128.81
123.73
116.09
115.80
81.49
77.58
77.16
76.74
24.06
^{13}C NMR (75.47 MHz, $CDCl_3$)
I
O
N
F
2-(2-(4-fluorophenyl)-2-iodoethoxy)isoindoline-1,3-dione 3da
180
170
160
150
140
130
120
110
100
90
80
70
60
50
40
30
20
10
ppm

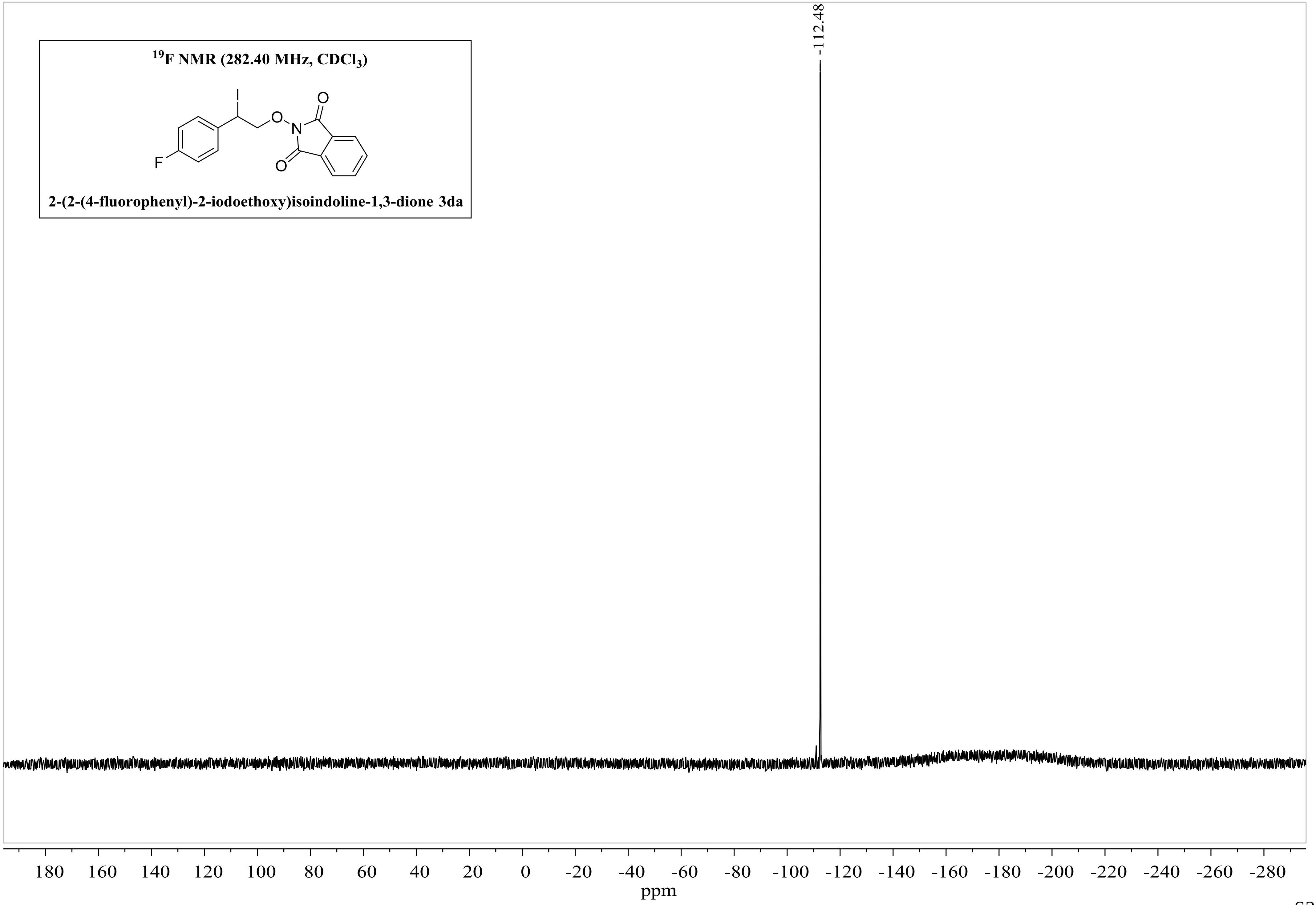

-112.48
^{19}F NMR (282.40 MHz, CDCl$_3$)
I
O
N
O
O
F
2-(2-(4-fluorophenyl)-2-iodoethoxy)isoindoline-1,3-dione 3da
180 160 140 120 100 80 60 40 20 0 -20 -40 -60 -80 -100 -120 -140 -160 -180 -200 -220 -240 -260 -280
ppm

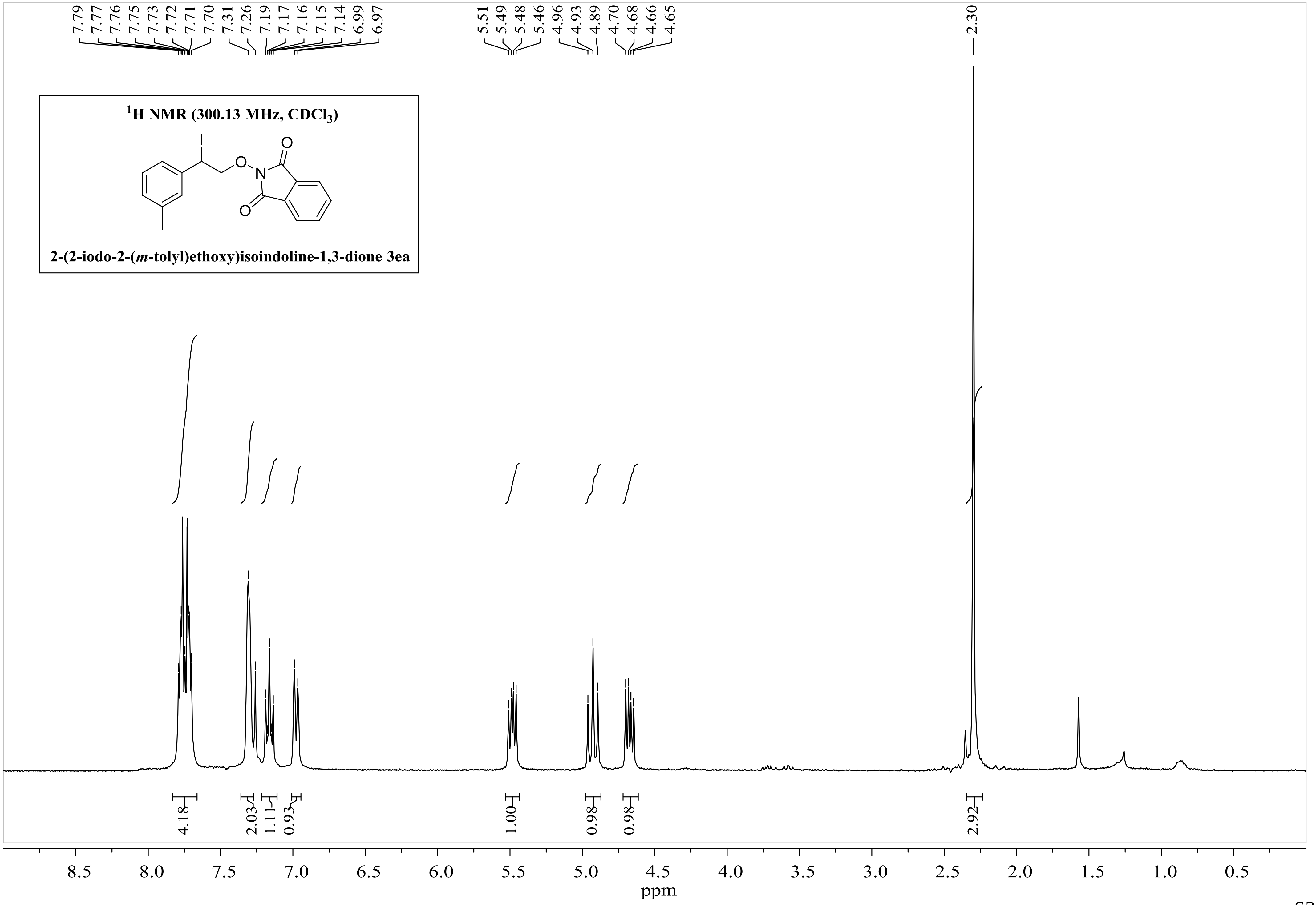
7.79 7.77 7.76 7.75 7.73 7.72 7.71 7.70 7.31 7.26 7.19 7.17 7.16 7.15 7.14 6.99 6.97
5.51 5.49 5.48 5.46 4.96 4.93 4.89 4.70 4.68 4.66 4.65
2.30
1H NMR (300.13 MHz, CDCl3)
2-(2-iodo-2-(m-tolyl)ethoxy)isoindoline-1,3-dione 3ea
4.18
2.03
1.11
0.93
1.00
0.98
0.98
2.92
8.5 8.0 7.5 7.0 6.5 6.0 5.5 5.0 4.5 4.0 3.5 3.0 2.5 2.0 1.5 1.0 0.5
ppm

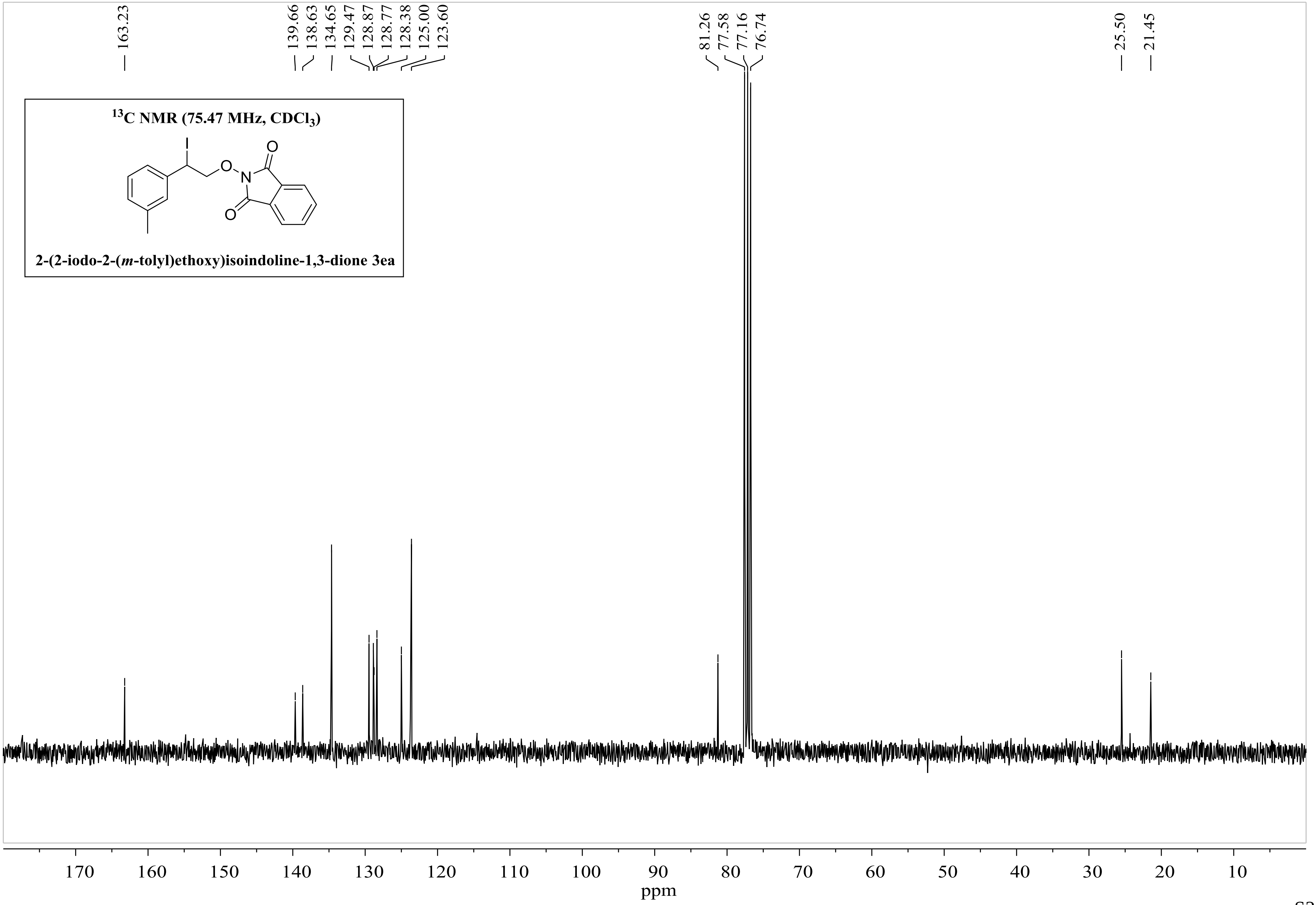
163.23
139.66
138.63
134.65
129.47
128.87
128.77
128.38
125.00
123.60
81.26
77.58
77.16
76.74
25.50
21.45
^{13}C NMR (75.47 MHz, CDCl$_3$)
I
O
N
O
O
2-(2-iodo-2-(*m*-tolyl)ethoxy)isoindoline-1,3-dione 3ea
170
160
150
140
130
120
110
100
90
80
70
60
50
40
30
20
10
ppm

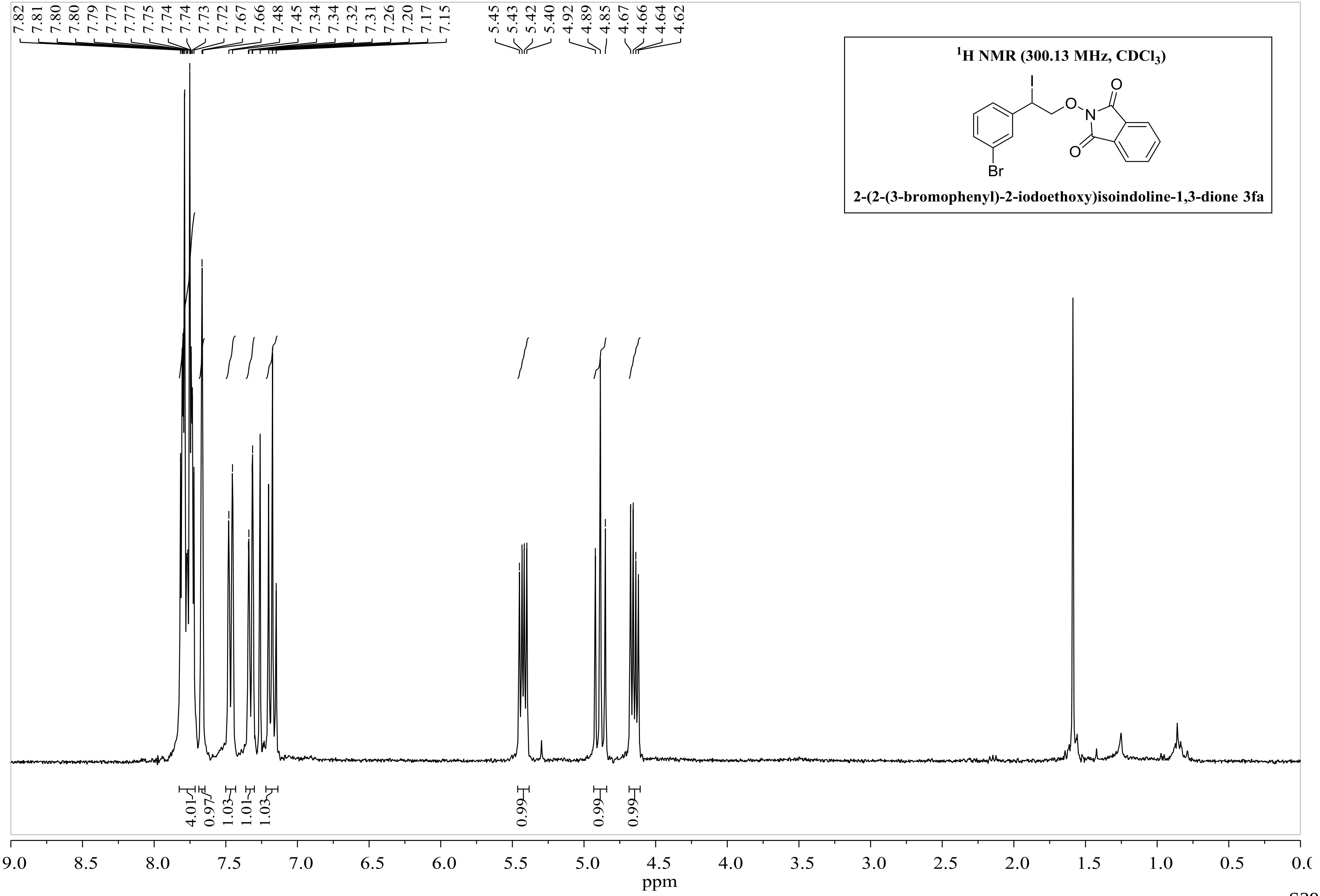
7.82 7.81 7.80 7.80 7.79 7.77 7.77 7.75 7.74 7.74 7.73 7.72 7.67 7.66 7.48 7.45 7.34 7.34 7.32 7.31 7.26 7.20 7.17 7.15
5.45 5.43 5.42 5.40 4.92 4.89 4.85 4.67 4.66 4.64 4.62
^{1}H NMR (300.13 MHz, CDCl$_3$)
I
O
N
O
O
Br
2-(2-(3-bromophenyl)-2-iodoethoxy)isoindoline-1,3-dione 3fa
4.01 0.97 1.03 1.01 1.03
0.99 0.99 0.99
9.0 8.5 8.0 7.5 7.0 6.5 6.0 5.5 5.0 4.5 4.0 3.5 3.0 2.5 2.0 1.5 1.0 0.5 0.0
ppm

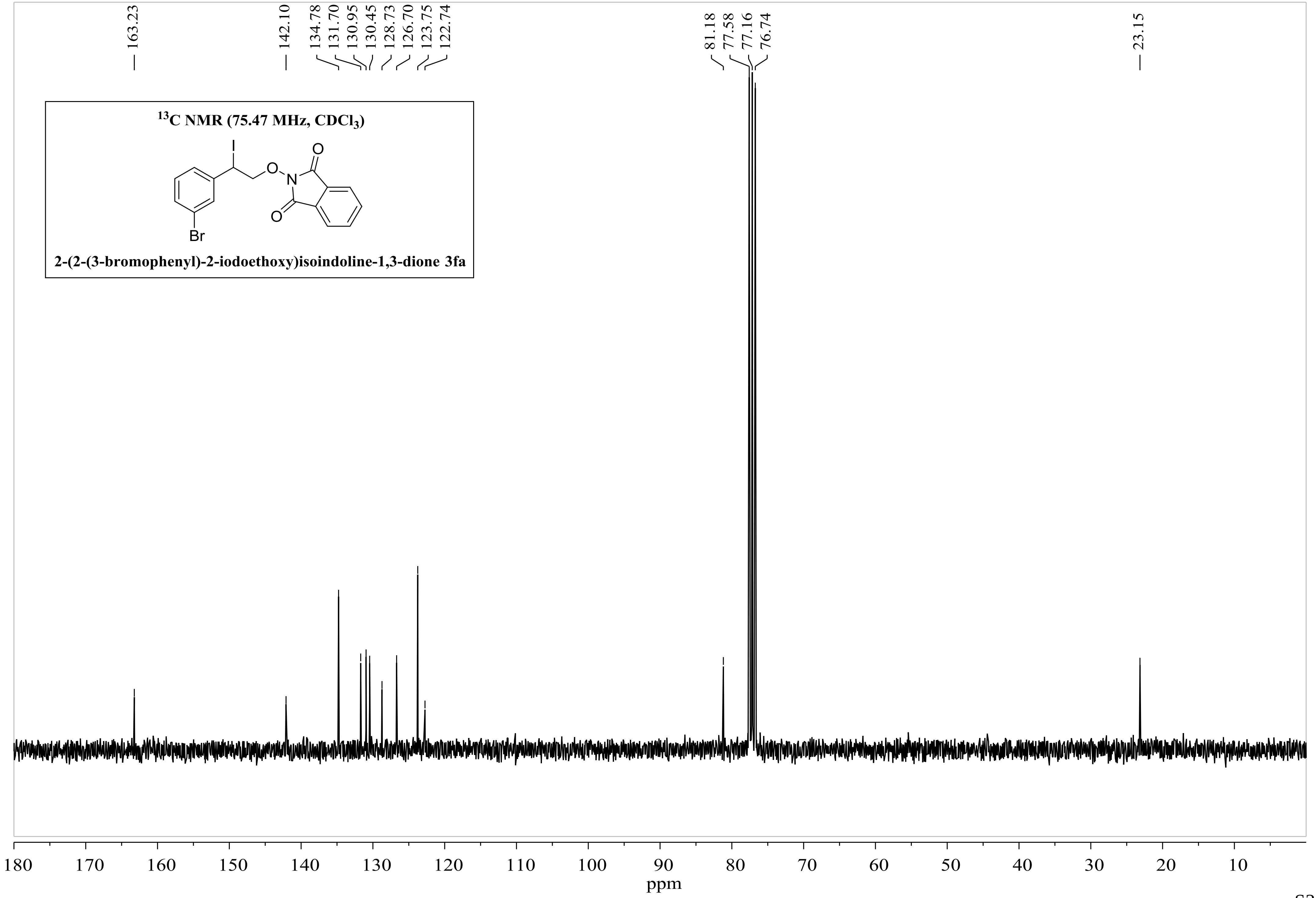

163.23
142.10
134.78
131.70
130.95
130.45
128.73
126.70
123.75
122.74
81.18
77.58
77.16
76.74
23.15
13C NMR (75.47 MHz, CDCl3)
I
O
O
N
O
Br
2-(2-(3-bromophenyl)-2-iodoethoxy)isoindoline-1,3-dione 3fa
180
170
160
150
140
130
120
110
100
90
80
70
60
50
40
30
20
10
ppm

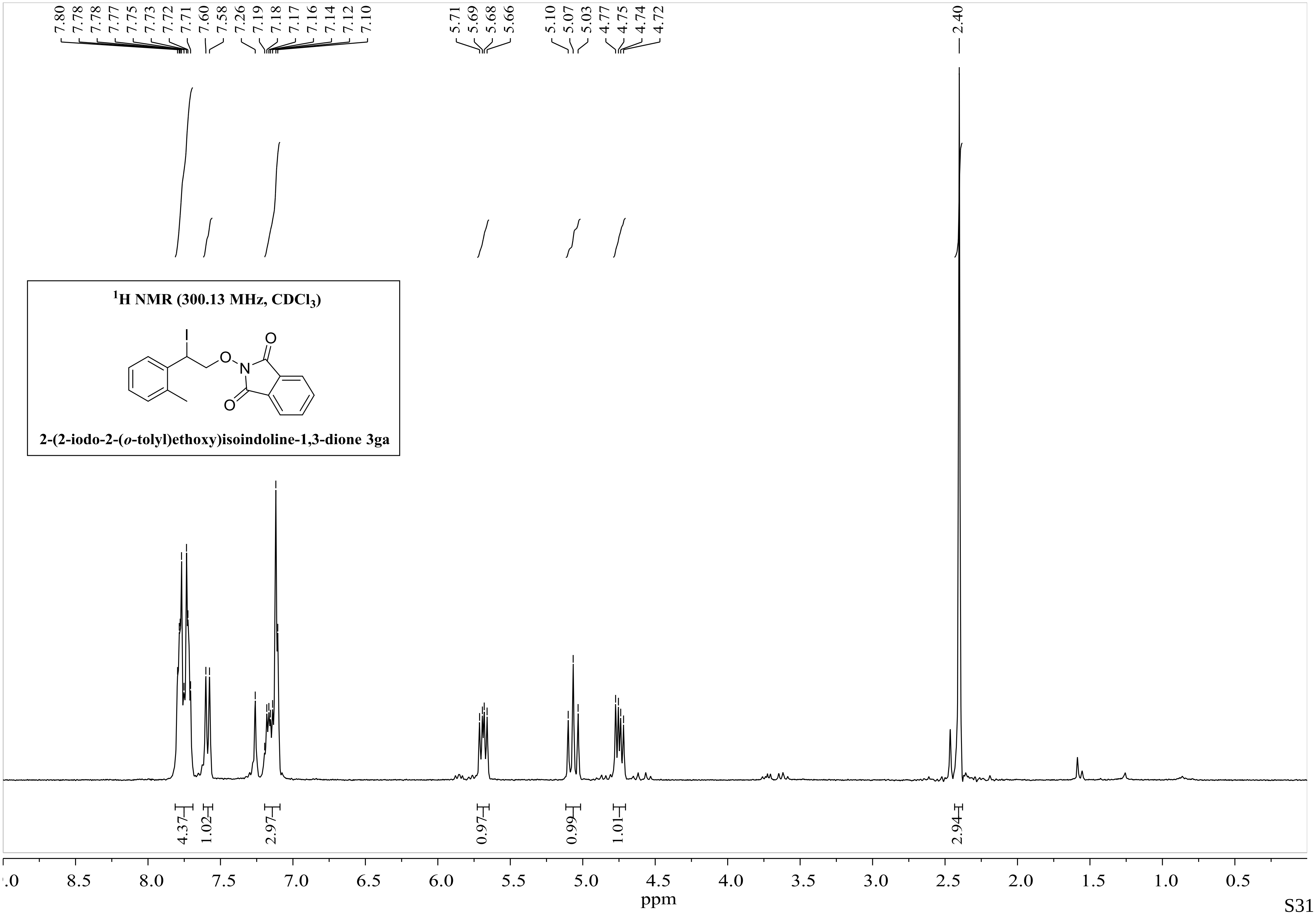
7.80 7.78 7.78 7.77 7.75 7.73 7.72 7.71 7.60 7.58 7.26 7.19 7.18 7.17 7.16 7.14 7.12 7.10
5.71 5.69 5.68 5.66
5.10 5.07 5.03 4.77 4.75 4.74 4.72
2.40
1H NMR (300.13 MHz, CDCl3)
I
O
N
O
O
2-(2-iodo-2-(o-tolyl)ethoxy)isoindoline-1,3-dione 3ga
4.37
1.02
2.97
0.97
0.99
1.01
2.94
8.5 8.0 7.5 7.0 6.5 6.0 5.5 5.0 4.5 4.0 3.5 3.0 2.5 2.0 1.5 1.0 0.5
ppm

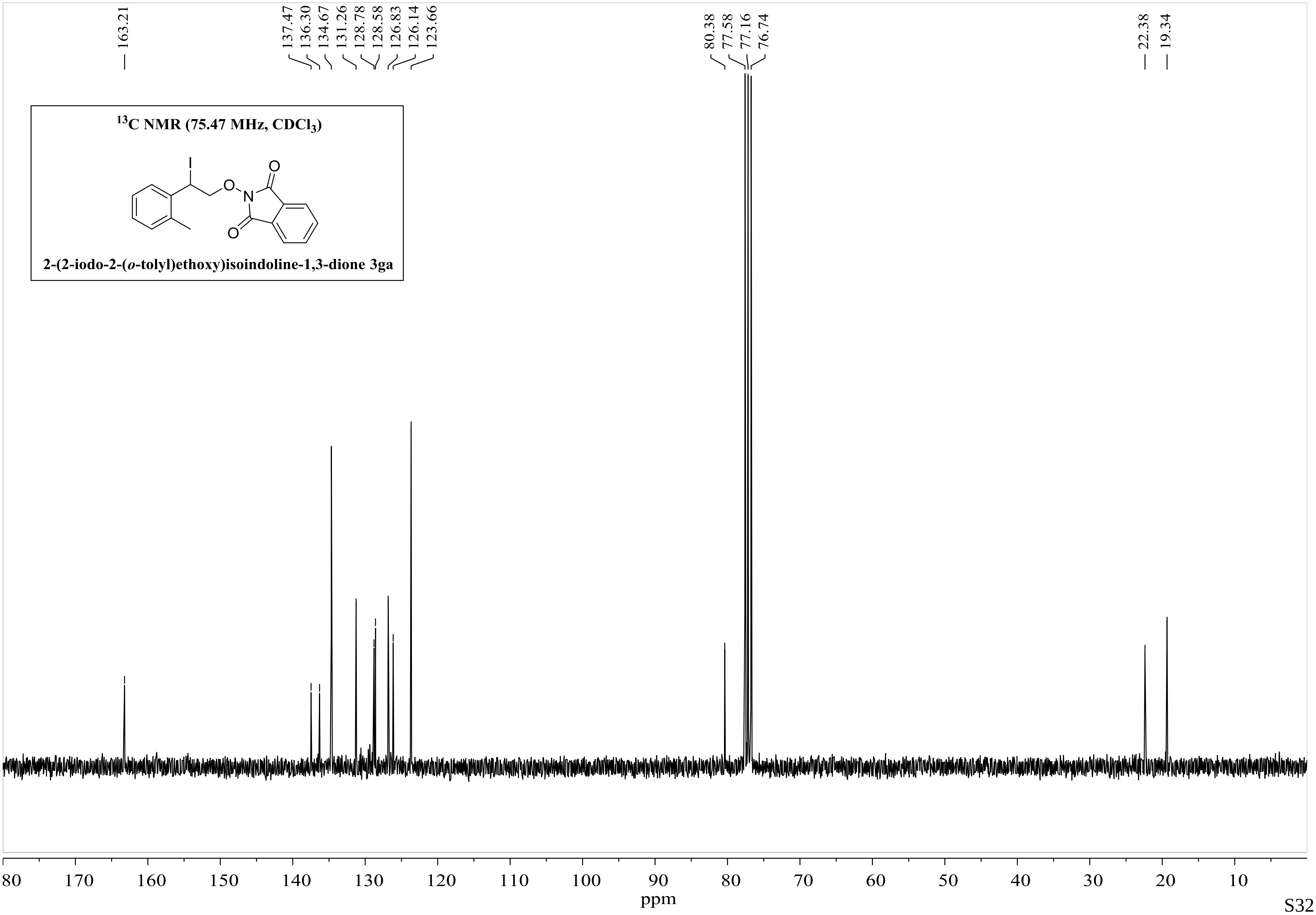
163.21
137.47
136.30
134.67
131.26
128.78
128.58
126.83
126.14
123.66
80.38
77.58
77.16
76.74
22.38
19.34
13C NMR (75.47 MHz, CDCl3)
I
O
O
N
O
2-(2-iodo-2-(o-tolyl)ethoxy)isoindoline-1,3-dione 3ga
80
170
160
150
140
130
120
110
100
90
80
70
60
50
40
30
20
10
ppm

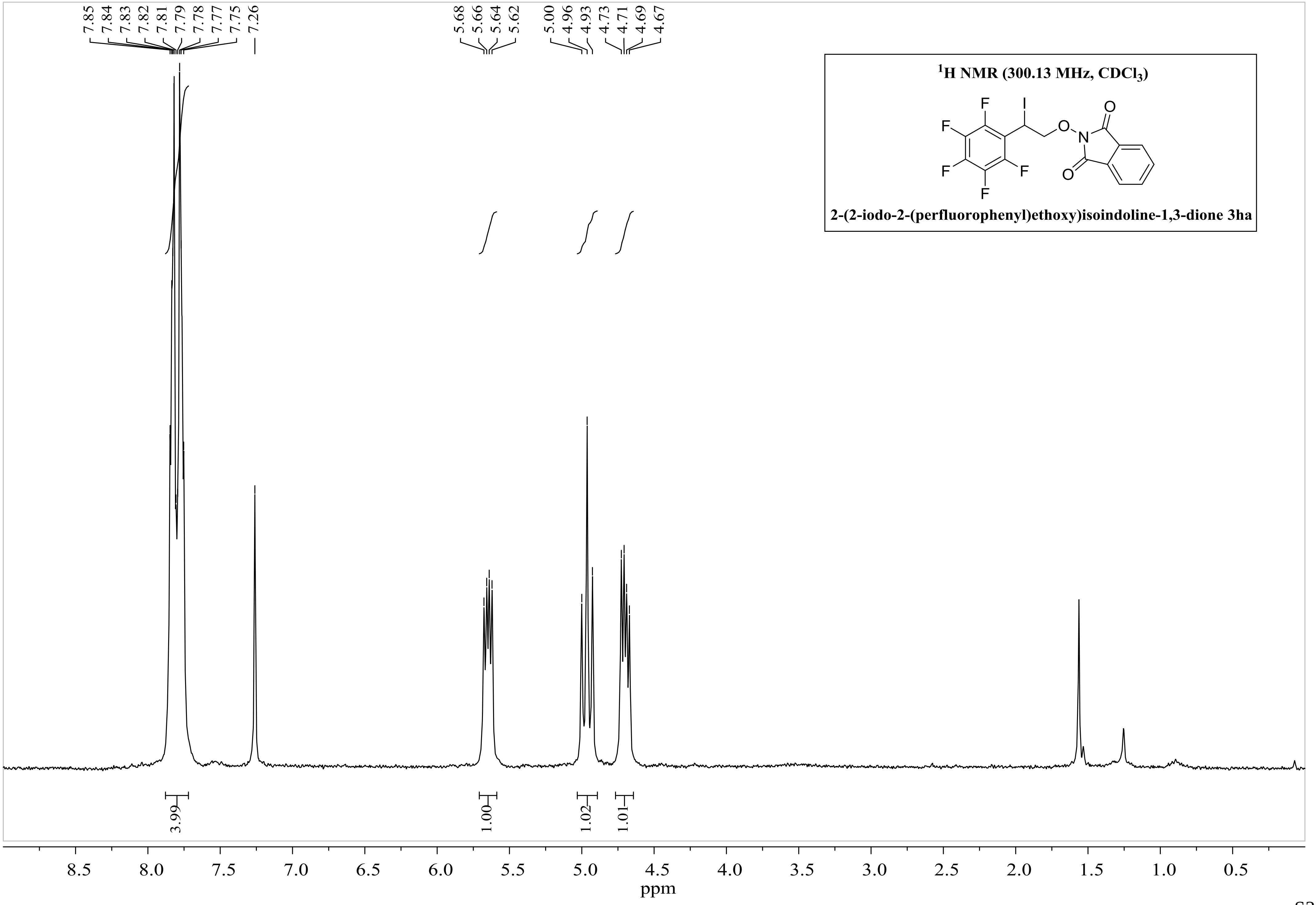

7.85 7.84 7.83 7.82 7.81 7.79 7.78 7.77 7.75 7.26
5.68 5.66 5.64 5.62
5.00 4.96 4.93 4.73 4.71 4.69 4.67
1H NMR (300.13 MHz, CDCl3)
2-(2-iodo-2-(perfluorophenyl)ethoxy)isoindoline-1,3-dione 3ha
3.99
1.00
1.02
1.01
8.5 8.0 7.5 7.0 6.5 6.0 5.5 5.0 4.5 4.0 3.5 3.0 2.5 2.0 1.5 1.0 0.5
ppm

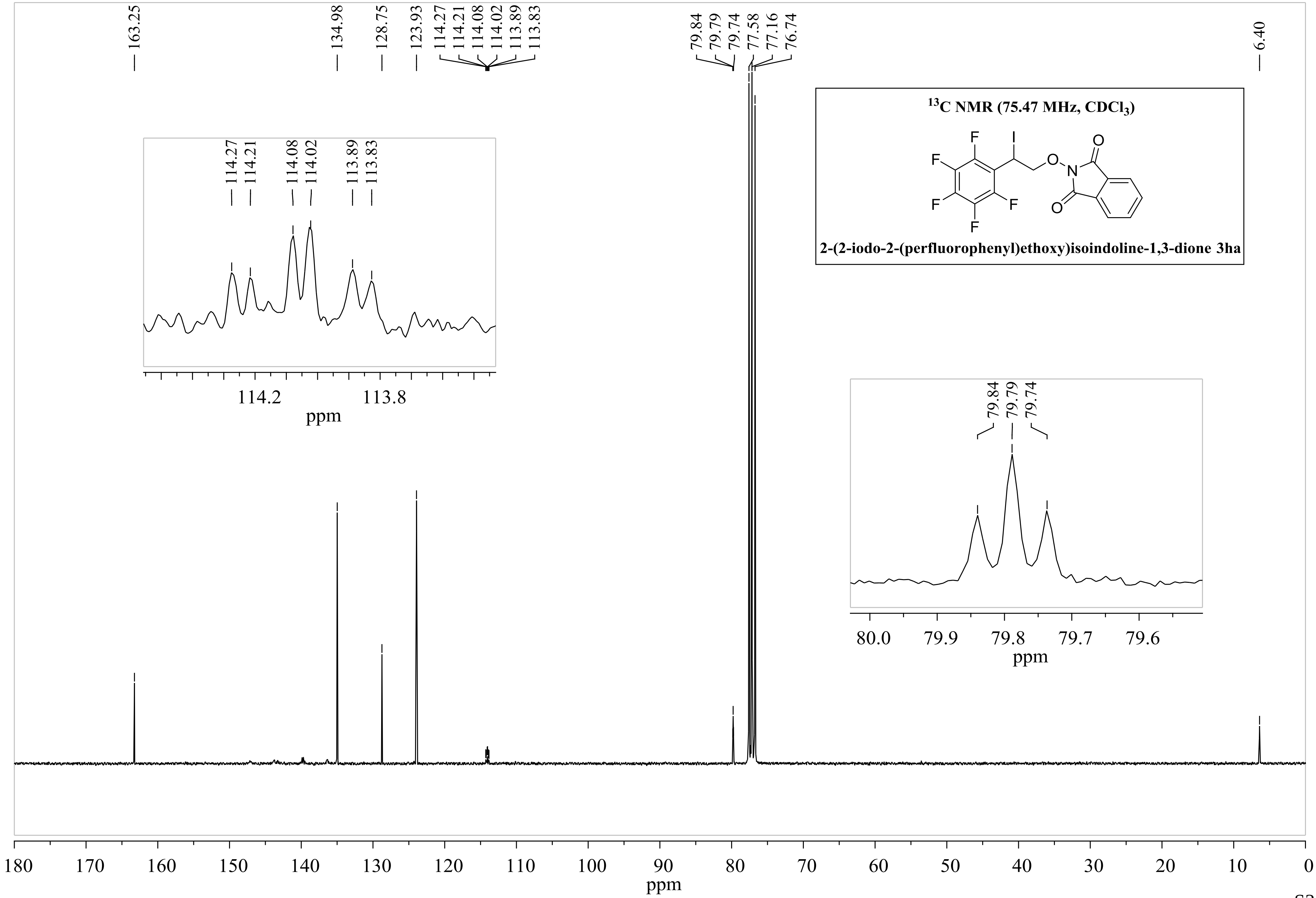

163.25
134.98
128.75
123.93
114.27
114.21
114.08
114.02
113.89
113.83
79.84
79.79
79.74
77.58
77.16
76.74
6.40
^{13}C NMR (75.47 MHz, CDCl$_3$)
2-(2-iodo-2-(perfluorophenyl)ethoxy)isoindoline-1,3-dione 3ha
114.2
113.8
ppm
80.0
79.9
79.8
79.7
79.6
ppm
180
170
160
150
140
130
120
110
100
90
80
70
60
50
40
30
20
10
0
ppm

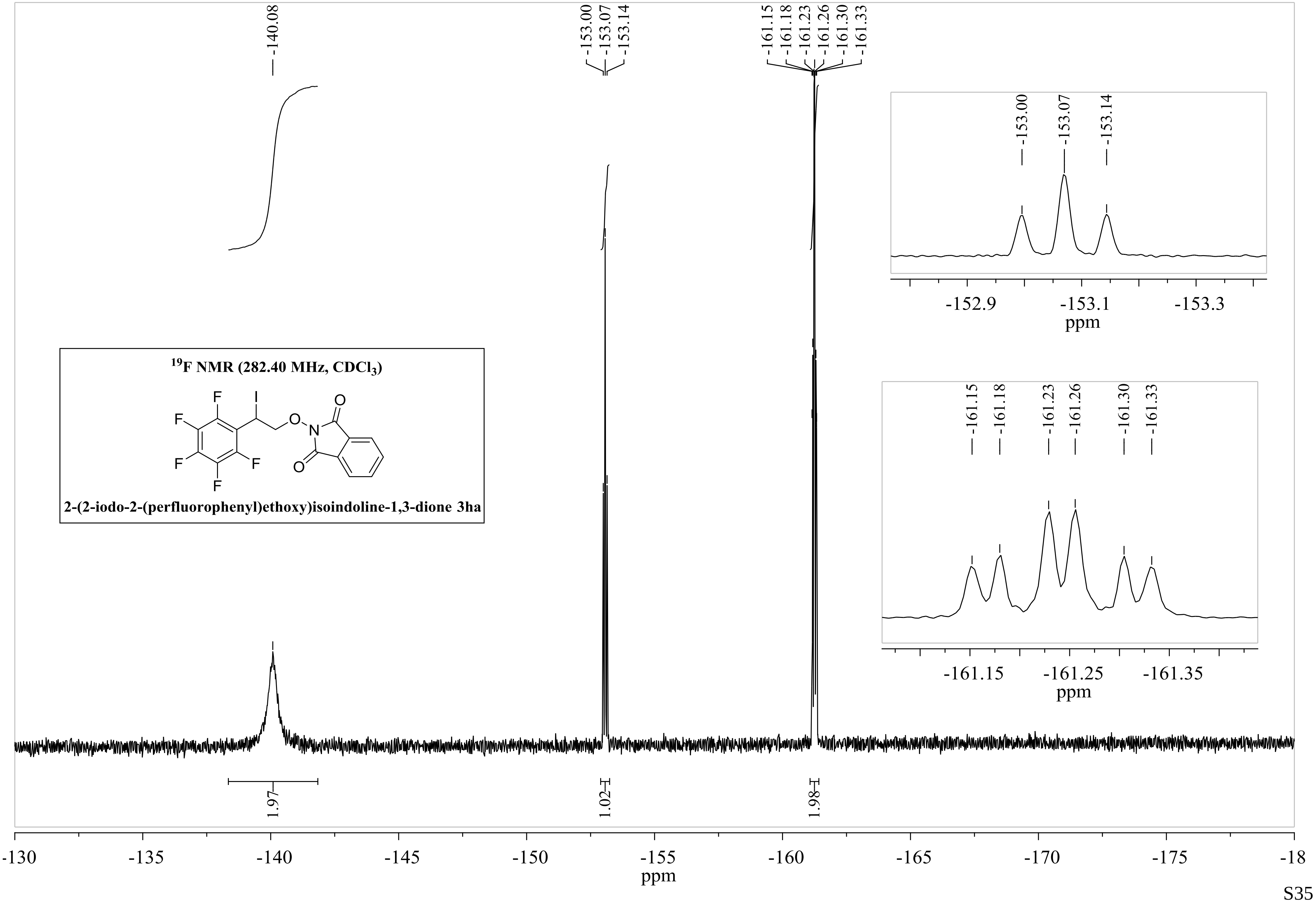

-140.08
-153.00
-153.07
-153.14
-161.15
-161.18
-161.23
-161.26
-161.30
-161.33
-152.9
-153.1
-153.3
ppm
-161.15
-161.25
-161.35
ppm
^{19}F NMR (282.40 MHz, CDCl$_3$)
F
I
O
F
O
N
F
F
F
O
F
2-(2-iodo-2-(perfluorophenyl)ethoxy)isoindoline-1,3-dione 3ha
1.97
1.02
1.98
-130
-135
-140
-145
-150
-155
-160
-165
-170
-175
-18
ppm

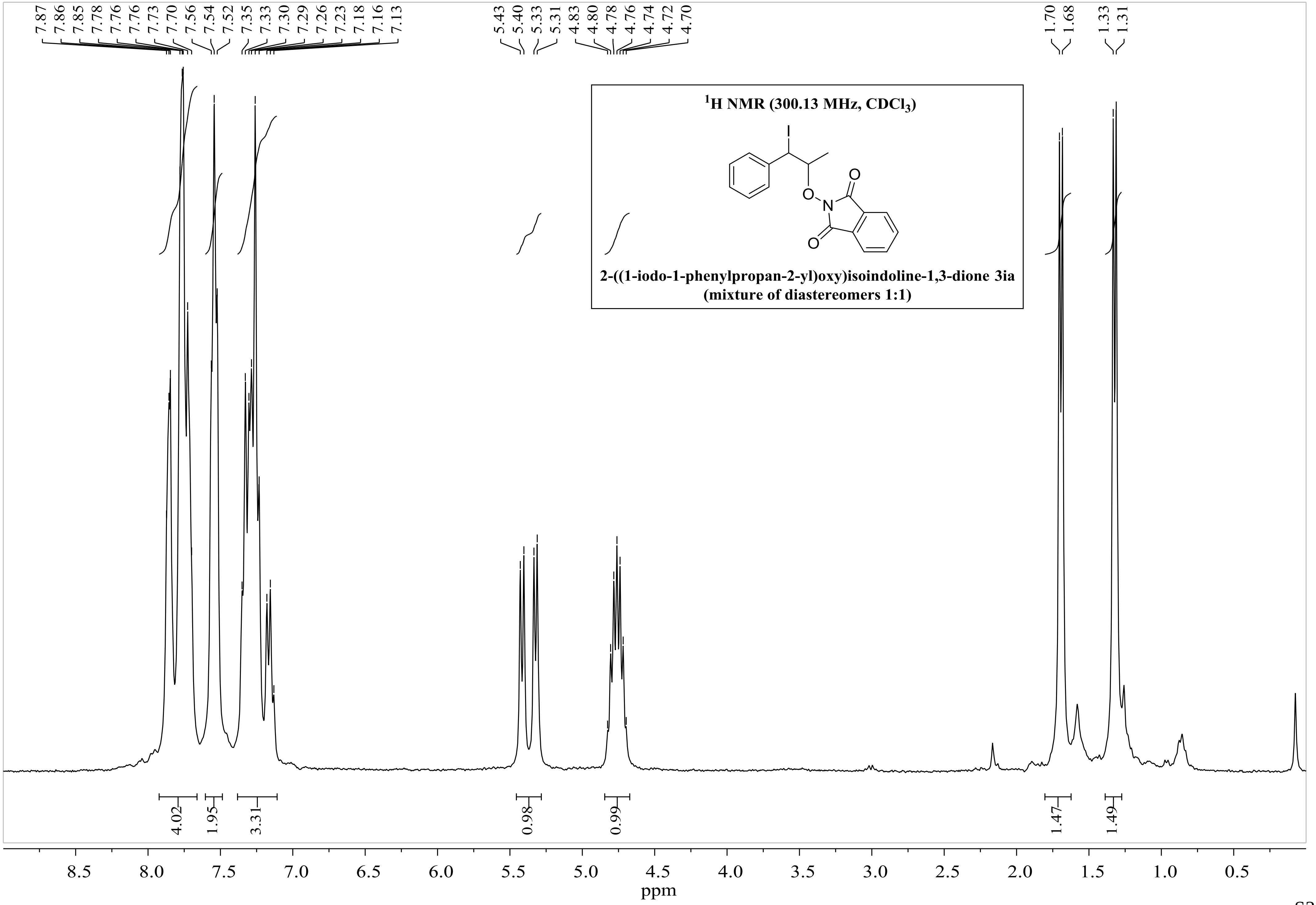

7.87 7.86 7.85 7.78 7.76 7.76 7.73 7.70 7.56 7.54 7.52 7.35 7.33 7.30 7.29 7.26 7.23 7.18 7.16 7.13
5.43 5.40 5.33 5.31
4.83 4.80 4.78 4.76 4.74 4.72 4.70
1.70 1.68
1.33 1.31
^{1}H NMR (300.13 MHz, $CDCl_3$)
I
O
N
O
O
2-((1-iodo-1-phenylpropan-2-yl)oxy)isoindoline-1,3-dione 3ia
(mixture of diastereomers 1:1)
4.02 1.95 3.31 0.98 0.99 1.47 1.49
8.5 8.0 7.5 7.0 6.5 6.0 5.5 5.0 4.5 4.0 3.5 3.0 2.5 2.0 1.5 1.0 0.5
ppm

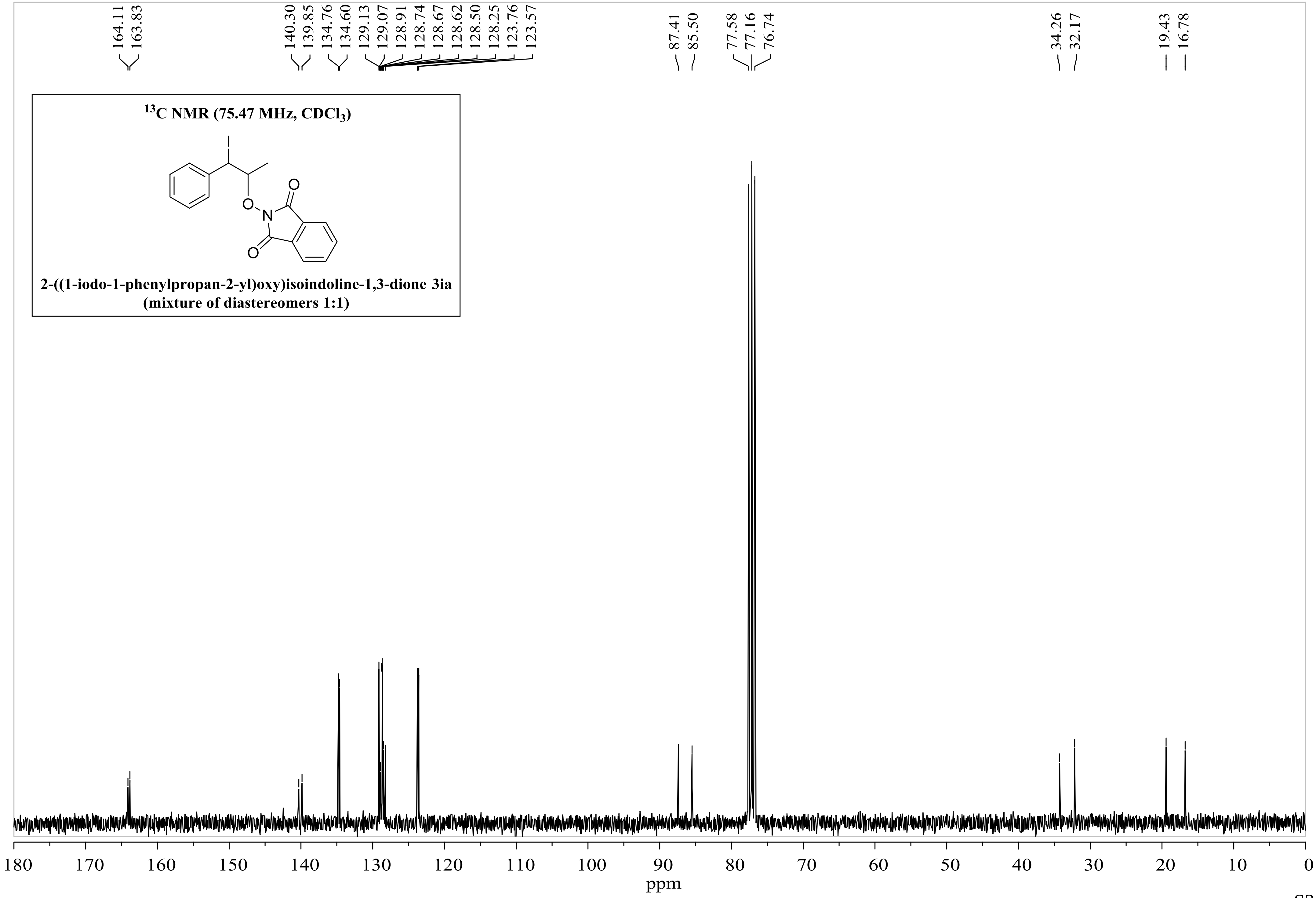
164.11
163.83
140.30
139.85
134.76
134.60
129.13
129.07
128.91
128.74
128.67
128.62
128.50
128.25
123.76
123.57
87.41
85.50
77.58
77.16
76.74
34.26
32.17
19.43
16.78
^{13}C NMR (75.47 MHz, $CDCl_3$)
I
O
O
N
O
2-((1-iodo-1-phenylpropan-2-yl)oxy)isoindoline-1,3-dione 3ia
(mixture of diastereomers 1:1)
180 170 160 150 140 130 120 110 100 90 80 70 60 50 40 30 20 10 0
ppm

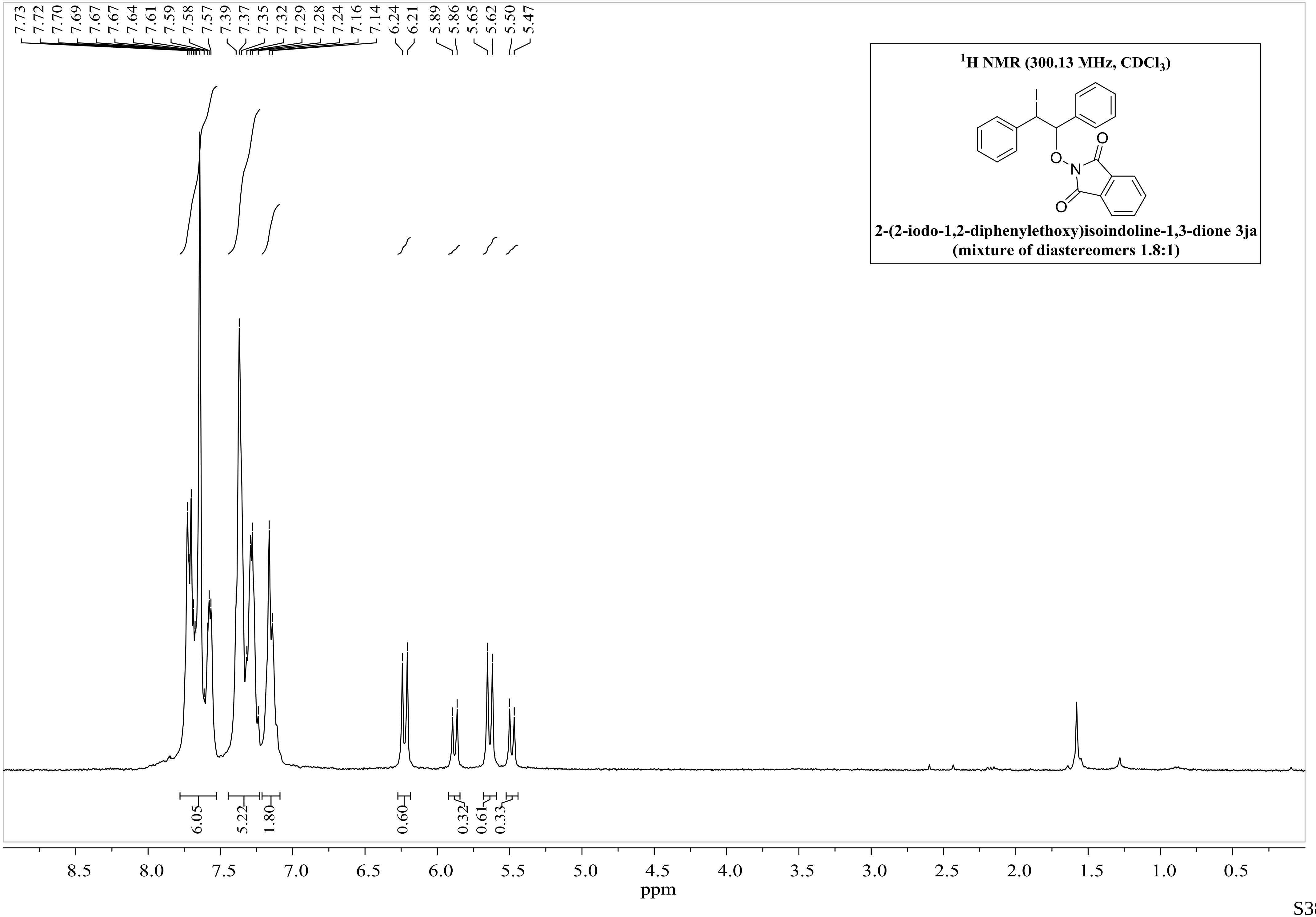
7.73 7.72 7.70 7.69 7.67 7.67 7.64 7.61 7.59 7.58 7.57 7.39 7.37 7.35 7.32 7.29 7.28 7.24 7.16 7.14 6.24 6.21 5.89 5.86 5.65 5.62 5.50 5.47
1H NMR (300.13 MHz, CDCl3)
I
O
N
O
O
2-(2-iodo-1,2-diphenylethoxy)isoindoline-1,3-dione 3ja
(mixture of diastereomers 1.8:1)
6.05
5.22
1.80
0.60
0.32
0.61
0.33
8.5 8.0 7.5 7.0 6.5 6.0 5.5 5.0 4.5 4.0 3.5 3.0 2.5 2.0 1.5 1.0 0.5
ppm

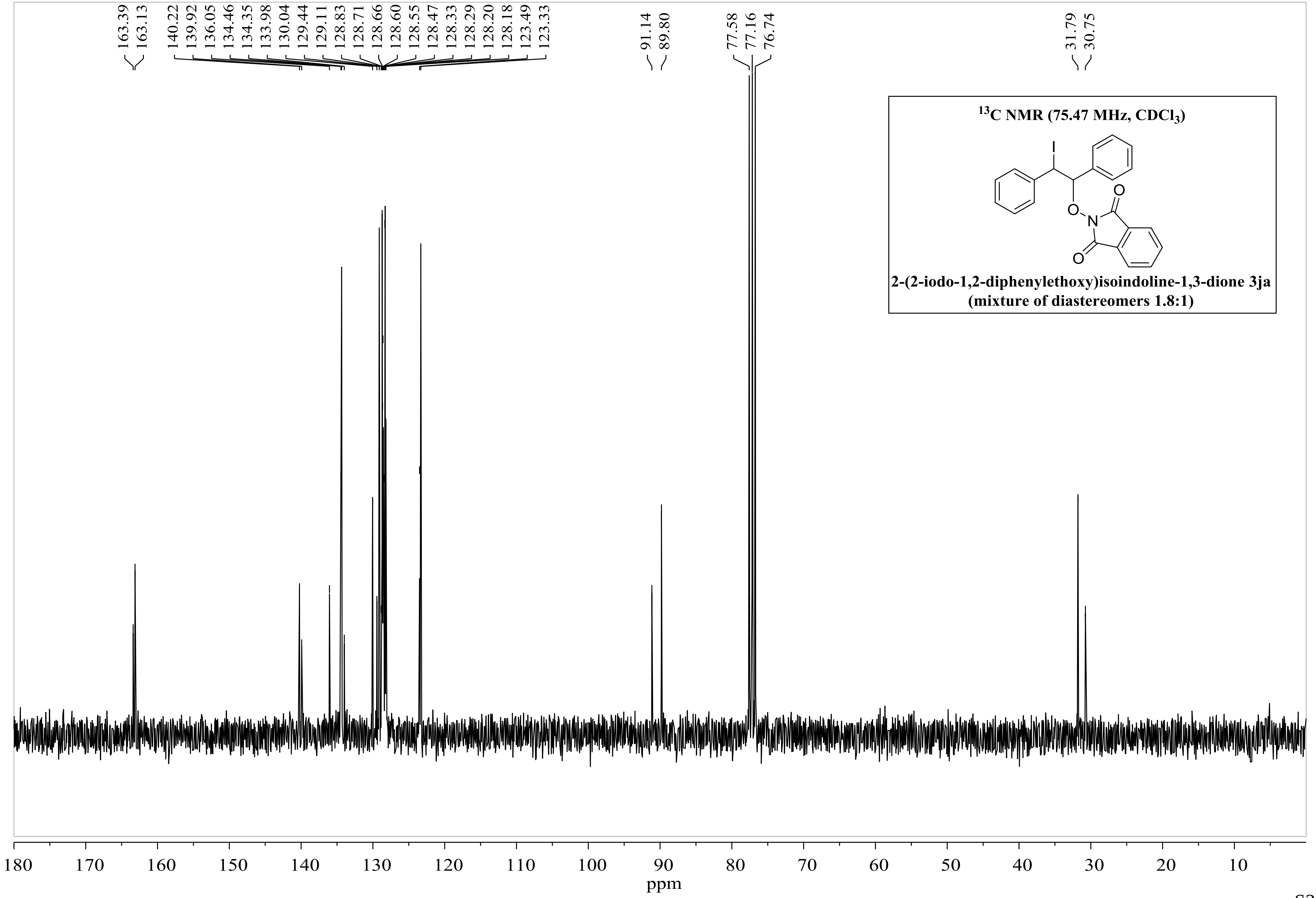

163.39
163.13
140.22
139.92
136.05
134.46
134.35
133.98
130.04
129.44
129.11
128.83
128.71
128.66
128.60
128.55
128.47
128.33
128.29
128.20
128.18
123.49
123.33
91.14
89.80
77.58
77.16
76.74
31.79
30.75
13C NMR (75.47 MHz, CDCl3)
I
O
O
N
O
O
2-(2-iodo-1,2-diphenylethoxy)isoindoline-1,3-dione 3ja
(mixture of diastereomers 1.8:1)
180 170 160 150 140 130 120 110 100 90 80 70 60 50 40 30 20 10
ppm

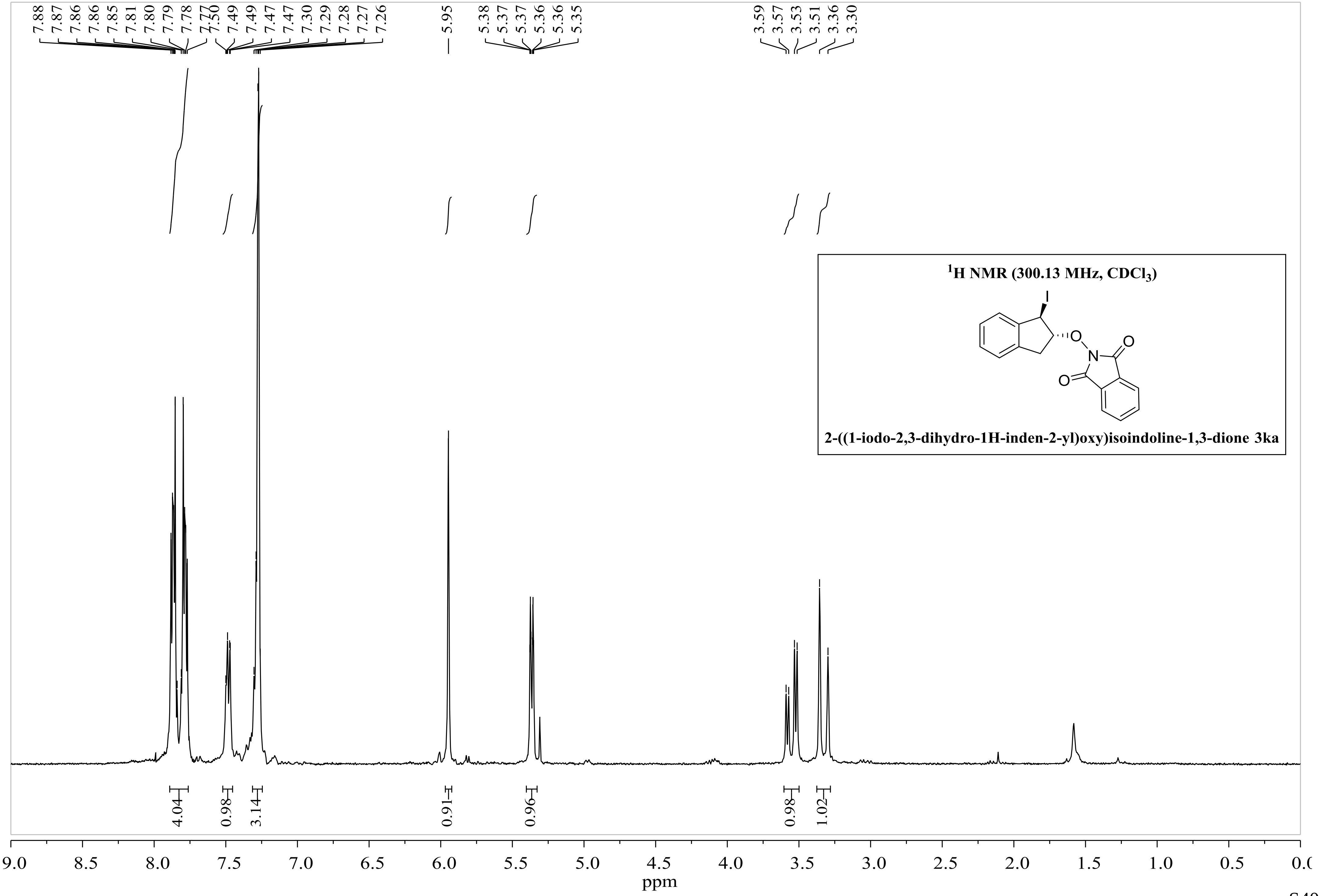

7.88 7.87 7.86 7.86 7.85 7.81 7.80 7.79 7.78 7.77 7.50 7.49 7.49 7.47 7.47 7.30 7.29 7.28 7.27 7.26
5.95
5.38 5.37 5.37 5.36 5.36 5.35
3.59 3.57 3.53 3.51 3.36 3.30
^{1}H NMR (300.13 MHz, $CDCl_3$)
I
O
N
O
O
2-((1-iodo-2,3-dihydro-1H-inden-2-yl)oxy)isoindoline-1,3-dione 3ka
4.04
0.98
3.14
0.91
0.96
0.98
1.02
9.0 8.5 8.0 7.5 7.0 6.5 6.0 5.5 5.0 4.5 4.0 3.5 3.0 2.5 2.0 1.5 1.0 0.5
ppm

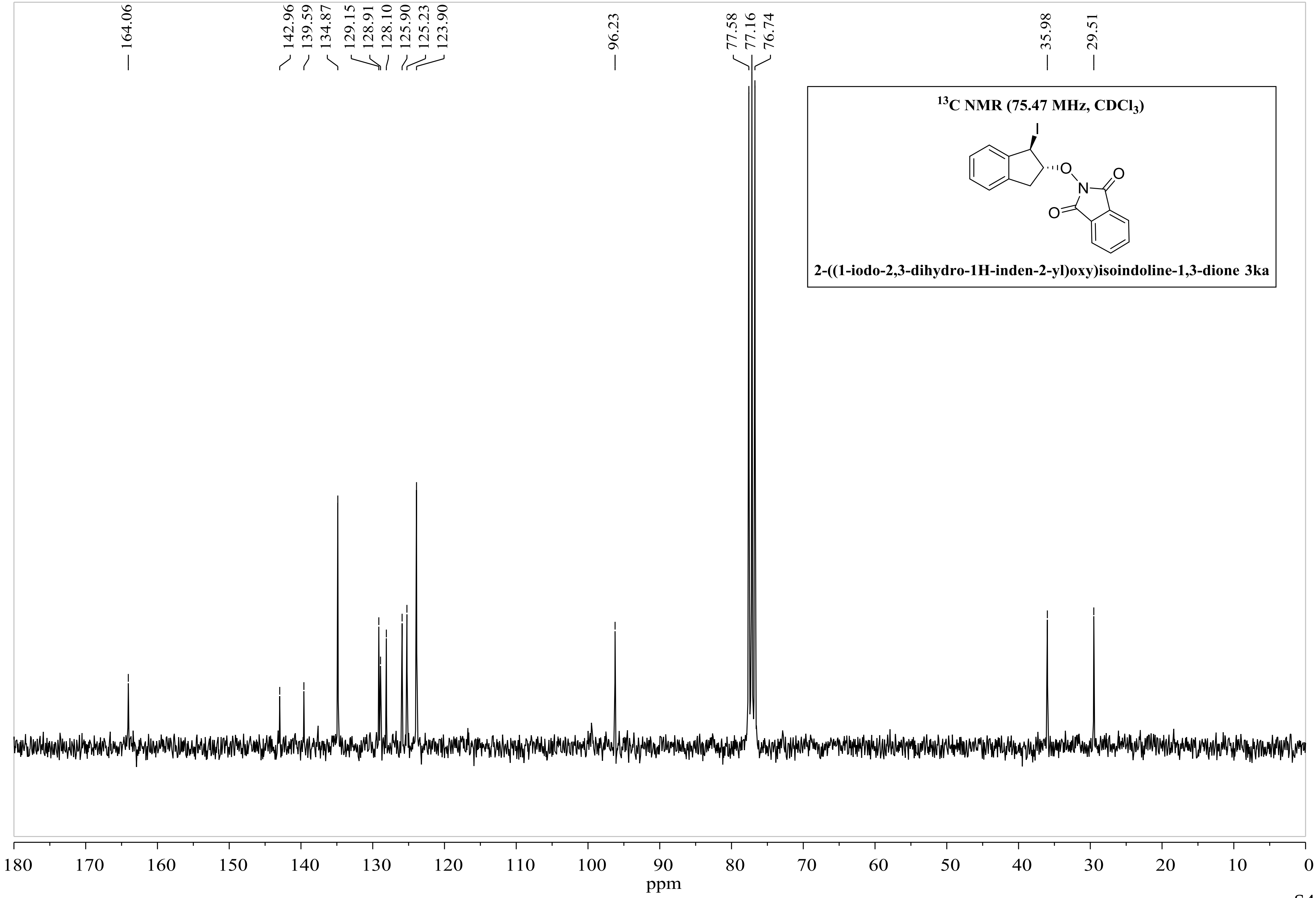
164.06
142.96
139.59
134.87
129.15
128.91
128.10
125.90
125.23
123.90
96.23
77.58
77.16
76.74
35.98
29.51
^{13}C NMR (75.47 MHz, CDCl$_3$)
I
O
N
O
O
2-((1-iodo-2,3-dihydro-1H-inden-2-yl)oxy)isoindoline-1,3-dione 3ka
180
170
160
150
140
130
120
110
100
90
80
70
60
50
40
30
20
10
0
ppm

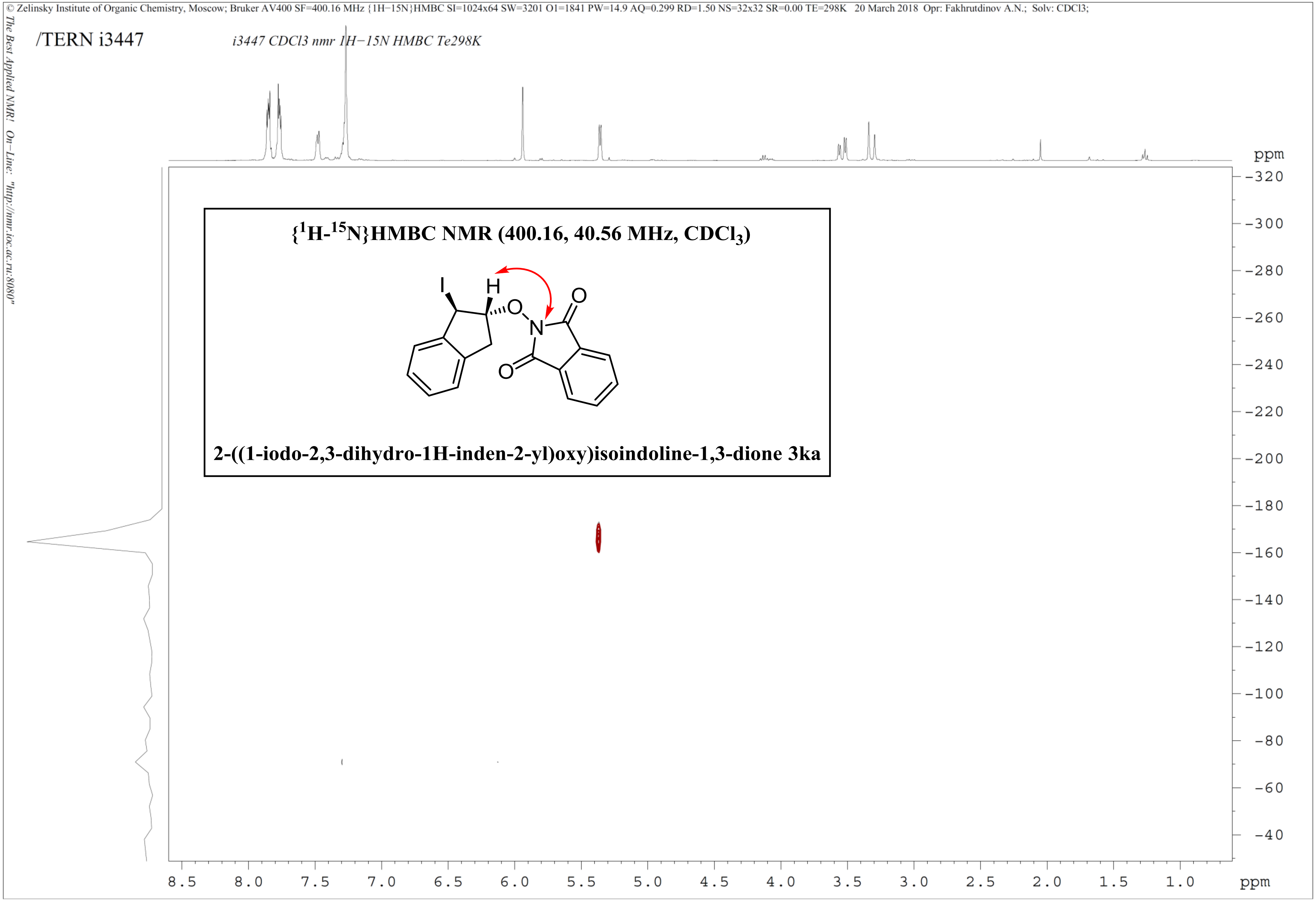
© Zelinsky Institute of Organic Chemistry, Moscow; Bruker AV400 SF=400.16 MHz {1H−15N}HMBC SI=1024x64 SW=3201 O1=1841 PW=14.9 AQ=0.299 RD=1.50 NS=32x32 SR=0.00 TE=298K 20 March 2018 Opr: Fakhrutdinov A.N.; Solv: CDCl3;
The Best Applied NMR! On−Line: "http://nmr.ioc.ac.ru:8080"
/TERN i3447
i3447 CDCl3 nmr 1H−15N HMBC Te298K
{1H-15N}HMBC NMR (400.16, 40.56 MHz, CDCl3)
I
H
O
N
O
O
2-((1-iodo-2,3-dihydro-1H-inden-2-yl)oxy)isoindoline-1,3-dione 3ka
ppm
-320
-300
-280
-260
-240
-220
-200
-180
-160
-140
-120
-100
-80
-60
-40
8.5
8.0
7.5
7.0
6.5
6.0
5.5
5.0
4.5
4.0
3.5
3.0
2.5
2.0
1.5
1.0
ppm

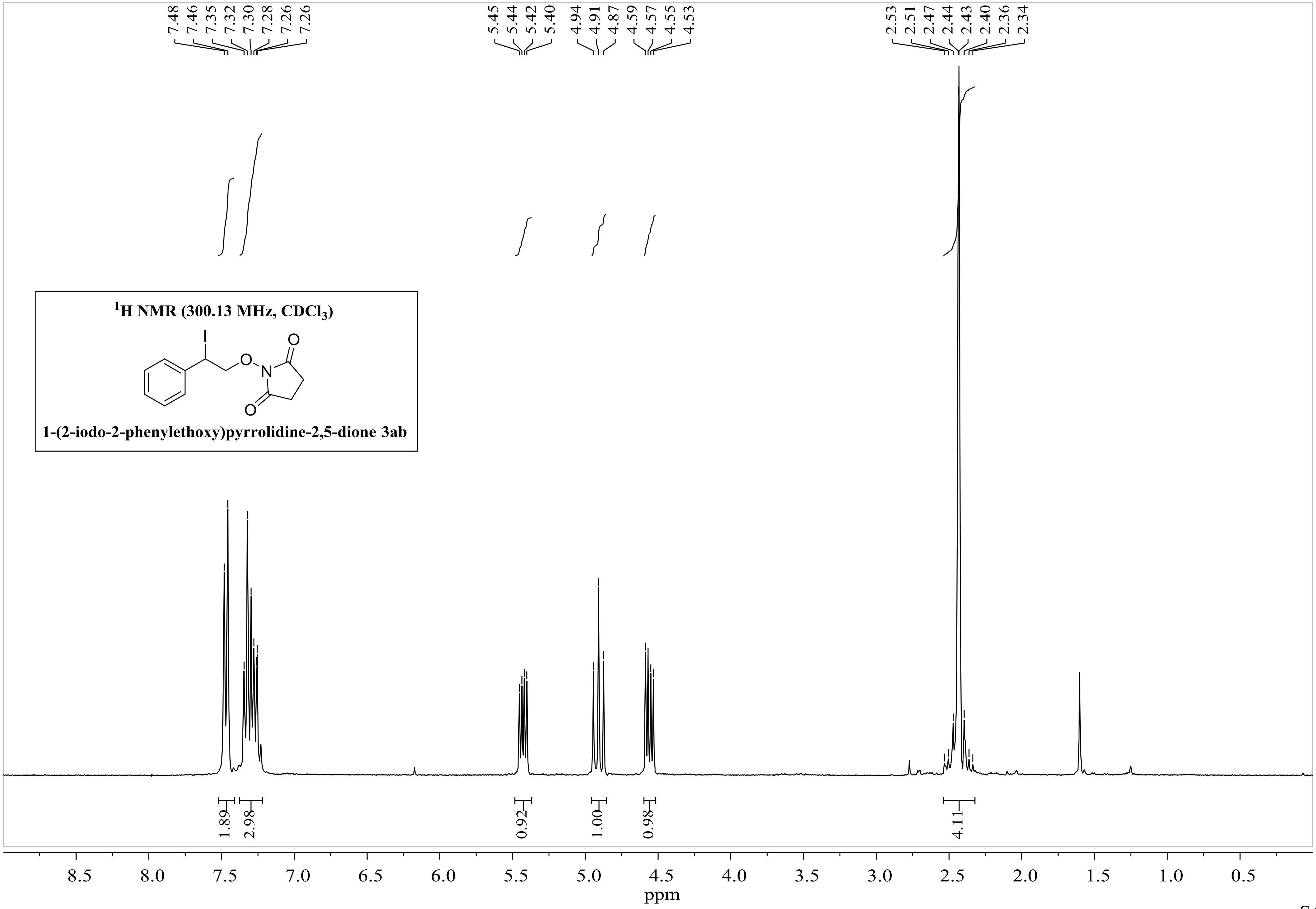
7.48 7.46 7.35 7.32 7.30 7.28 7.26 7.26
5.45 5.44 5.42 5.40 4.94 4.91 4.87 4.59 4.57 4.55 4.53
2.53 2.51 2.47 2.44 2.43 2.40 2.36 2.34
1H NMR (300.13 MHz, CDCl3)
1-(2-iodo-2-phenylethoxy)pyrrolidine-2,5-dione 3ab
1.89 2.98 0.92 1.00 0.98 4.11
8.5 8.0 7.5 7.0 6.5 6.0 5.5 5.0 4.5 4.0 3.5 3.0 2.5 2.0 1.5 1.0 0.5
ppm

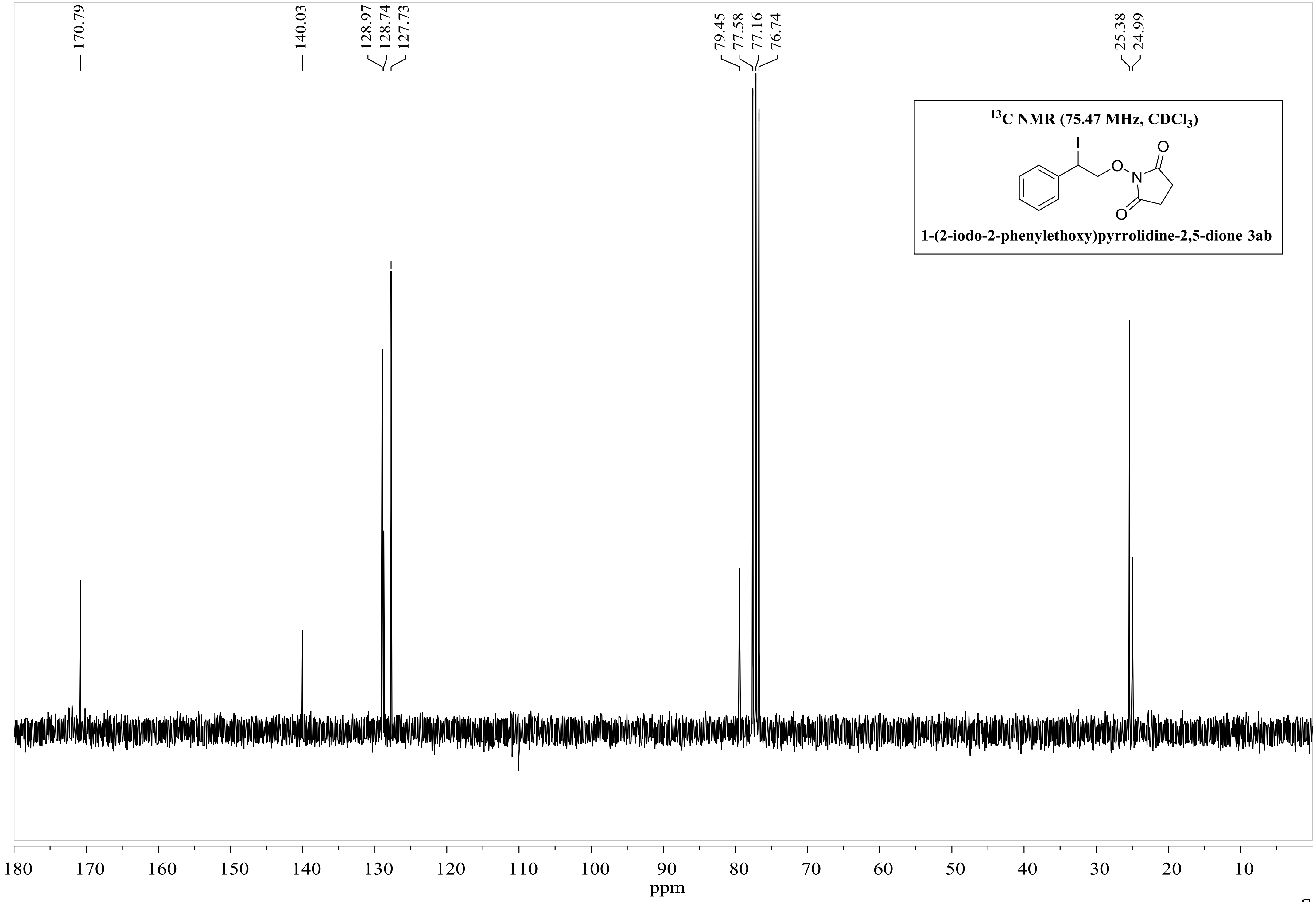
170.79
140.03
128.97
128.74
127.73
79.45
77.58
77.16
76.74
25.38
24.99
^{13}C NMR (75.47 MHz, CDCl$_3$)
I
O
N
O
O
1-(2-iodo-2-phenylethoxy)pyrrolidine-2,5-dione 3ab
180
170
160
150
140
130
120
110
100
90
80
70
60
50
40
30
20
10
ppm

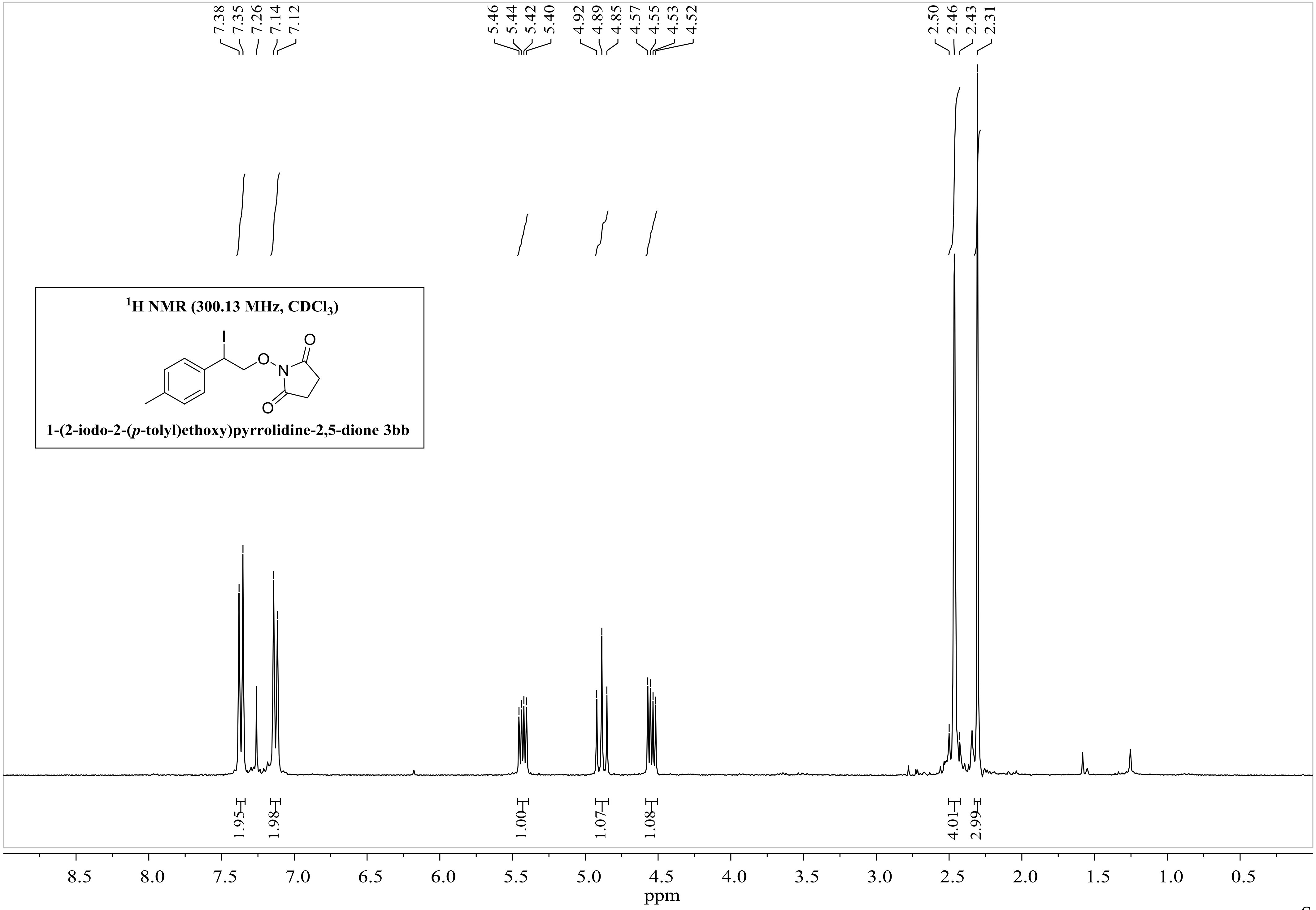

7.38
7.35
7.26
7.14
7.12
5.46
5.44
5.42
5.40
4.92
4.89
4.85
4.57
4.55
4.53
4.52
2.50
2.46
2.43
2.31
^{1}H NMR (300.13 MHz, CDCl$_3$)
1-(2-iodo-2-(*p*-tolyl)ethoxy)pyrrolidine-2,5-dione 3bb
1.95
1.98
1.00
1.07
1.08
4.01
2.99
8.5
8.0
7.5
7.0
6.5
6.0
5.5
5.0
4.5
4.0
3.5
3.0
2.5
2.0
1.5
1.0
0.5
ppm

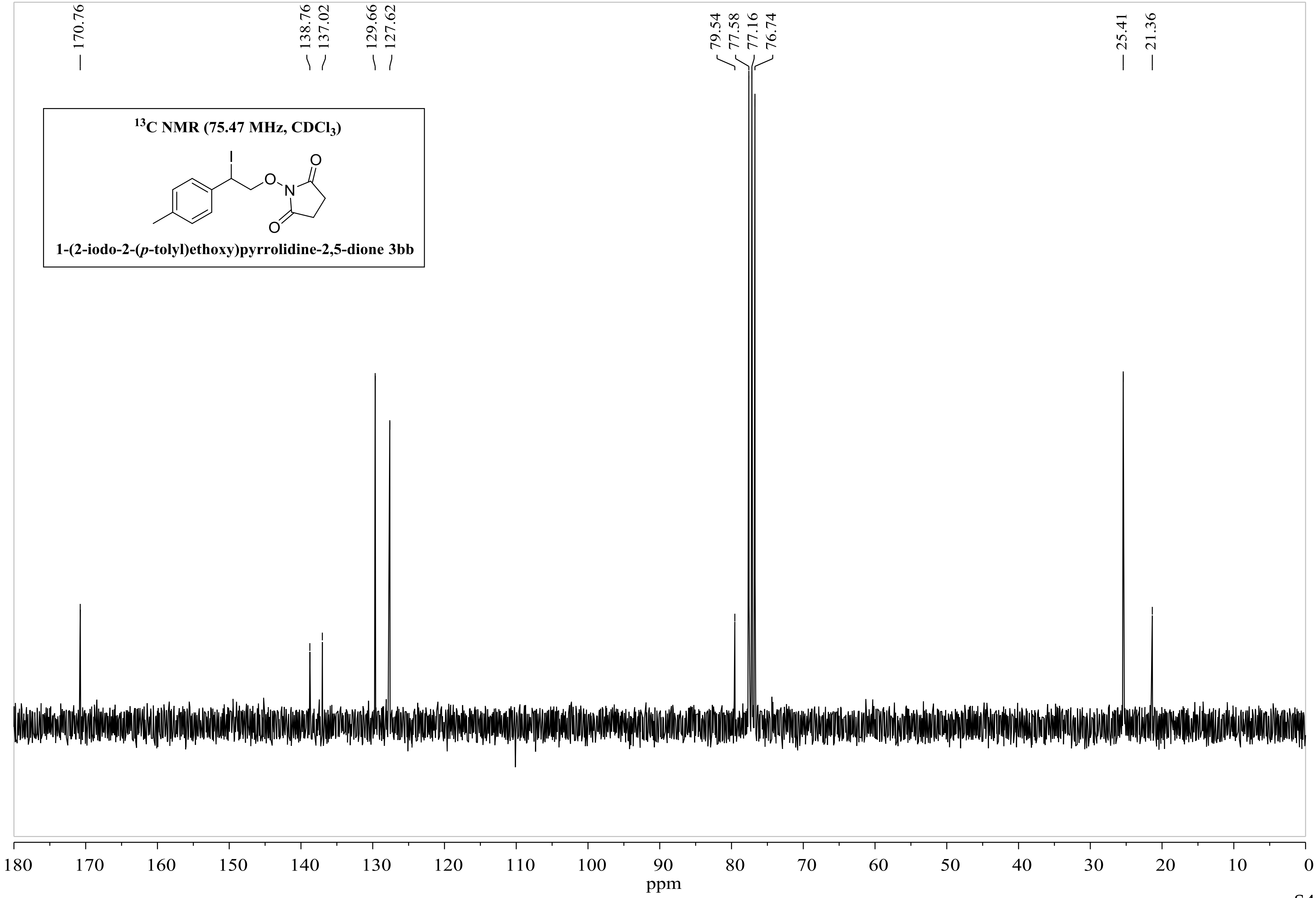

170.76
138.76
137.02
129.66
127.62
79.54
77.58
77.16
76.74
25.41
21.36
^{13}C NMR (75.47 MHz, CDCl$_3$)
I
O
N
1-(2-iodo-2-(*p*-tolyl)ethoxy)pyrrolidine-2,5-dione 3bb
180
170
160
150
140
130
120
110
100
90
80
70
60
50
40
30
20
10
0
ppm

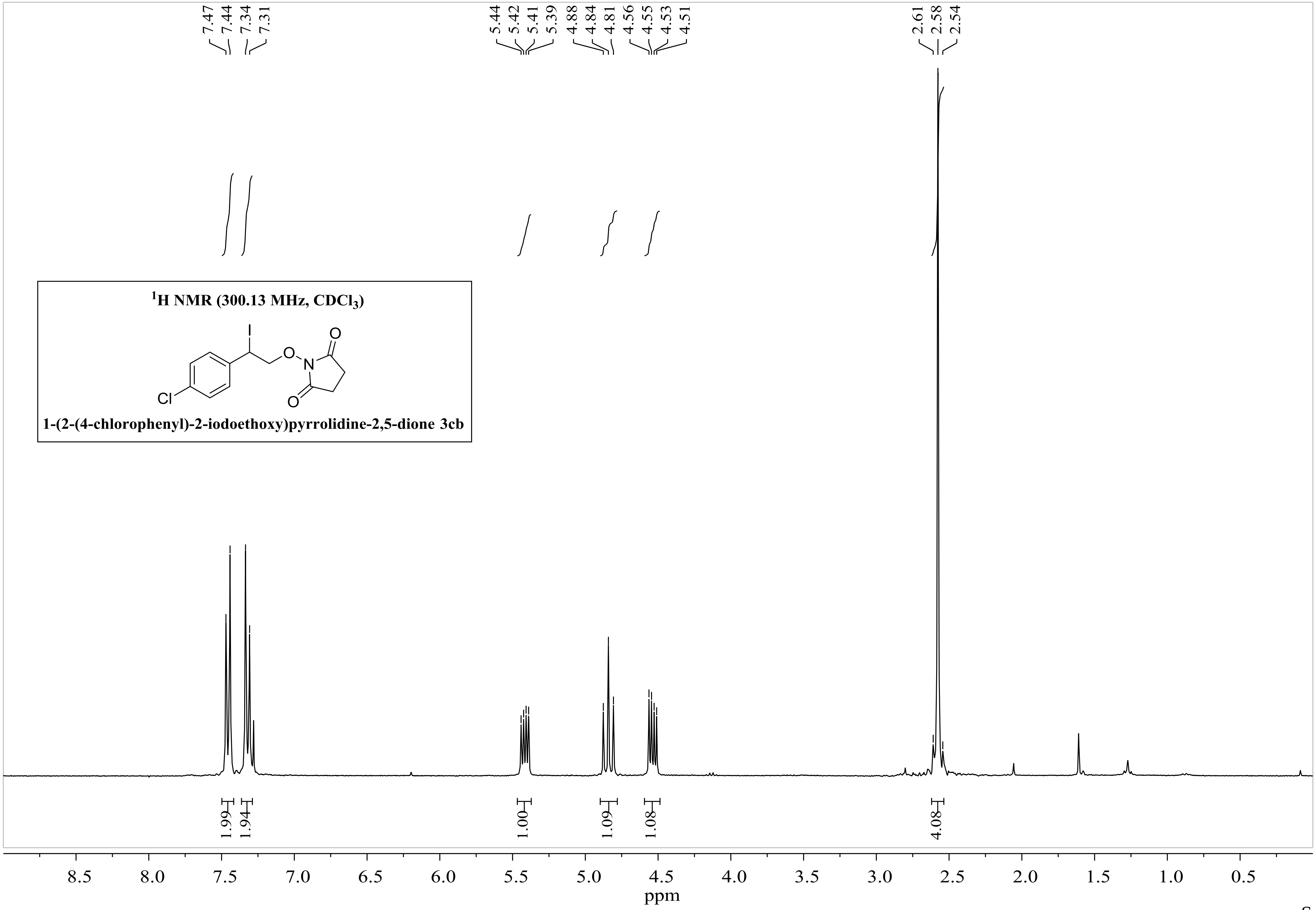

7.47
7.44
7.34
7.31
5.44
5.42
5.41
5.39
4.88
4.84
4.81
4.56
4.55
4.53
4.51
2.61
2.58
2.54
1H NMR (300.13 MHz, CDCl3)
I
O
O
N
Cl
O
1-(2-(4-chlorophenyl)-2-iodoethoxy)pyrrolidine-2,5-dione 3cb
1.99
1.94
1.00
1.09
1.08
4.08
8.5
8.0
7.5
7.0
6.5
6.0
5.5
5.0
4.5
4.0
3.5
3.0
2.5
2.0
1.5
1.0
0.5
ppm

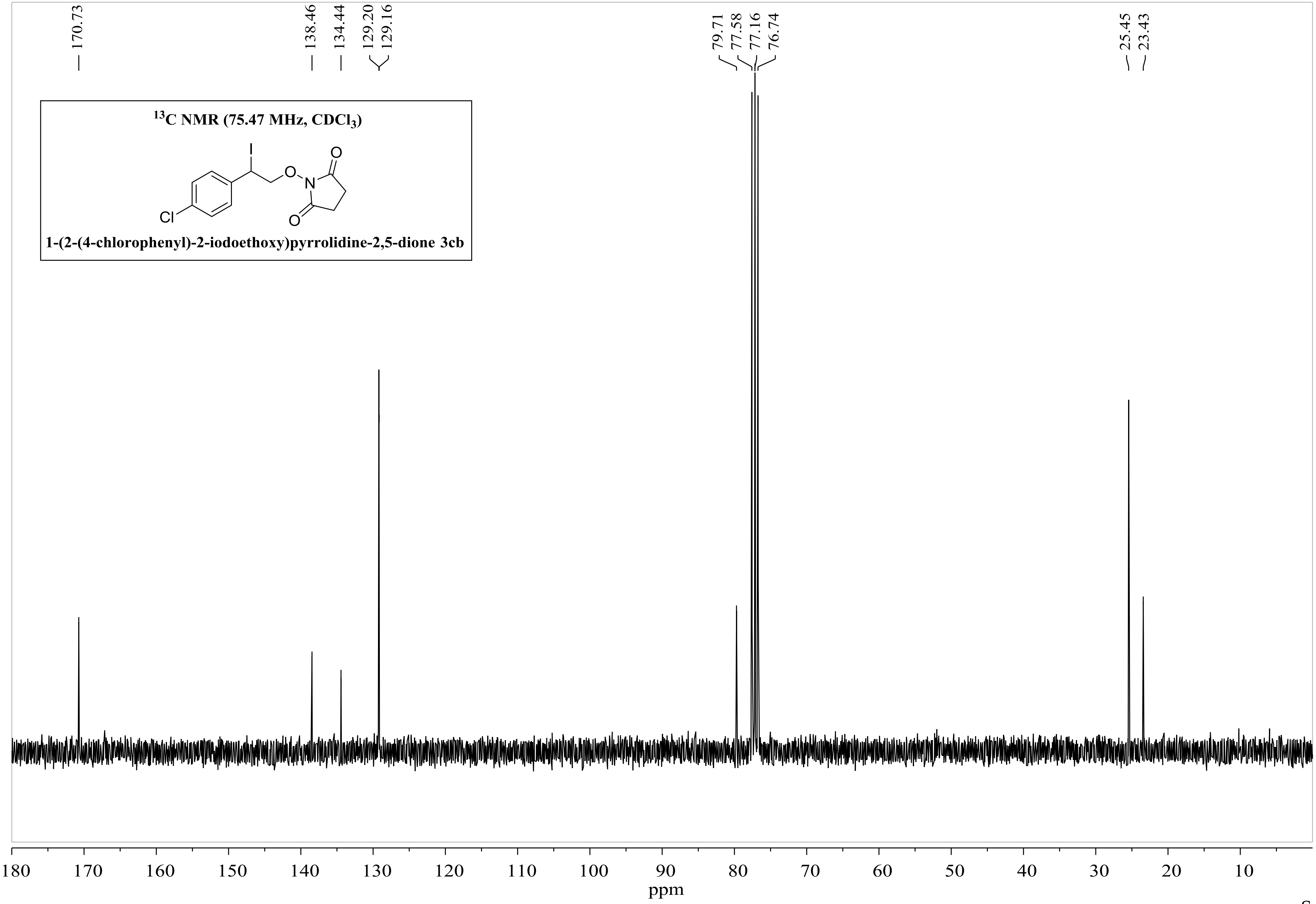

170.73
138.46
134.44
129.20
129.16
79.71
77.58
77.16
76.74
25.45
23.43
13C NMR (75.47 MHz, CDCl3)
I
O
N
Cl
1-(2-(4-chlorophenyl)-2-iodoethoxy)pyrrolidine-2,5-dione 3cb
180 170 160 150 140 130 120 110 100 90 80 70 60 50 40 30 20 10
ppm

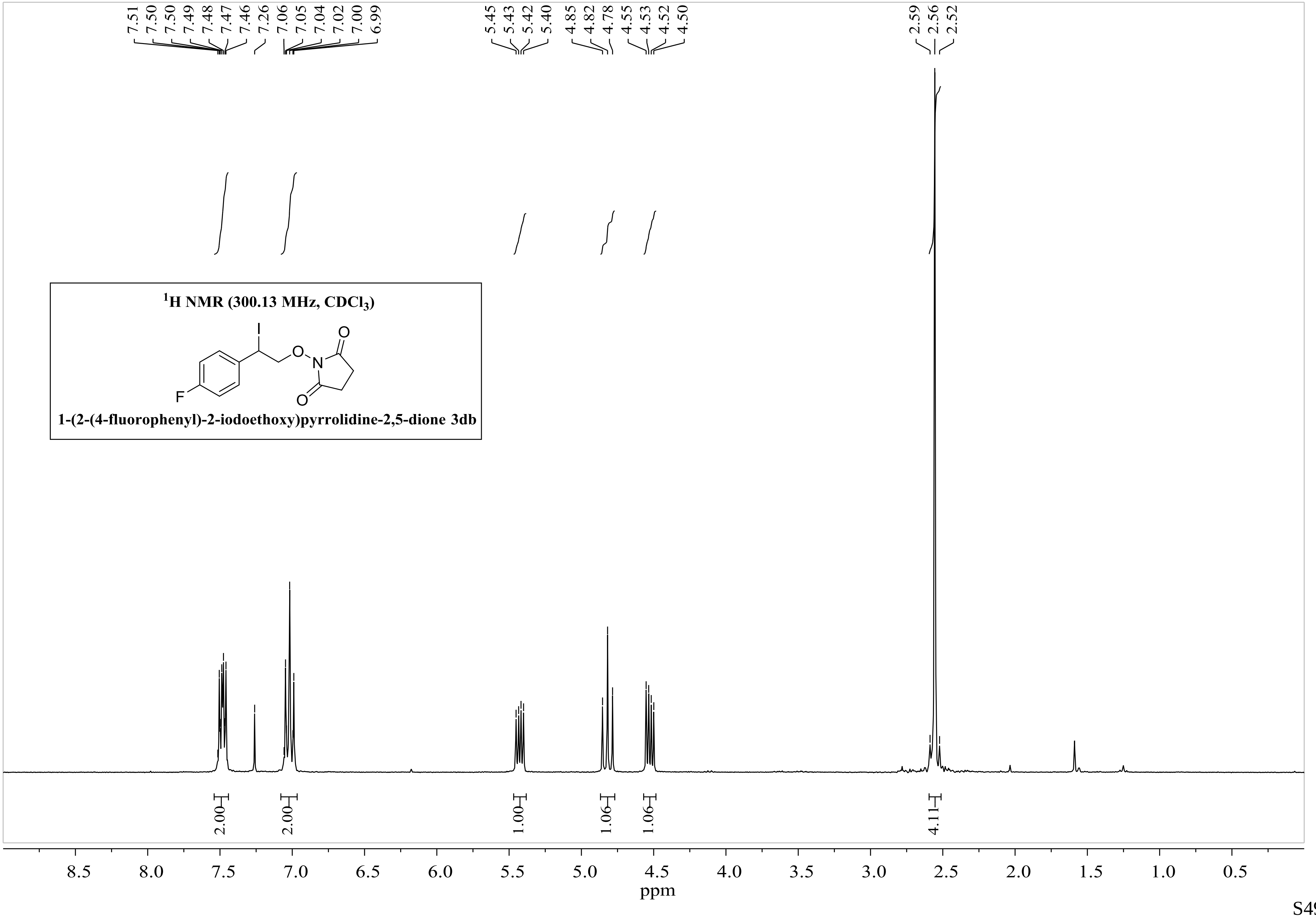
7.51 7.50 7.50 7.49 7.48 7.47 7.46 7.26 7.06 7.05 7.04 7.02 7.00 6.99
5.45 5.43 5.42 5.40 4.85 4.82 4.78 4.55 4.53 4.52 4.50
2.59 2.56 2.52
^{1}H NMR (300.13 MHz, CDCl$_3$)
I
O
N
O
O
F
1-(2-(4-fluorophenyl)-2-iodoethoxy)pyrrolidine-2,5-dione 3db
2.00
2.00
1.00
1.06
1.06
4.11
8.5 8.0 7.5 7.0 6.5 6.0 5.5 5.0 4.5 4.0 3.5 3.0 2.5 2.0 1.5 1.0 0.5
ppm

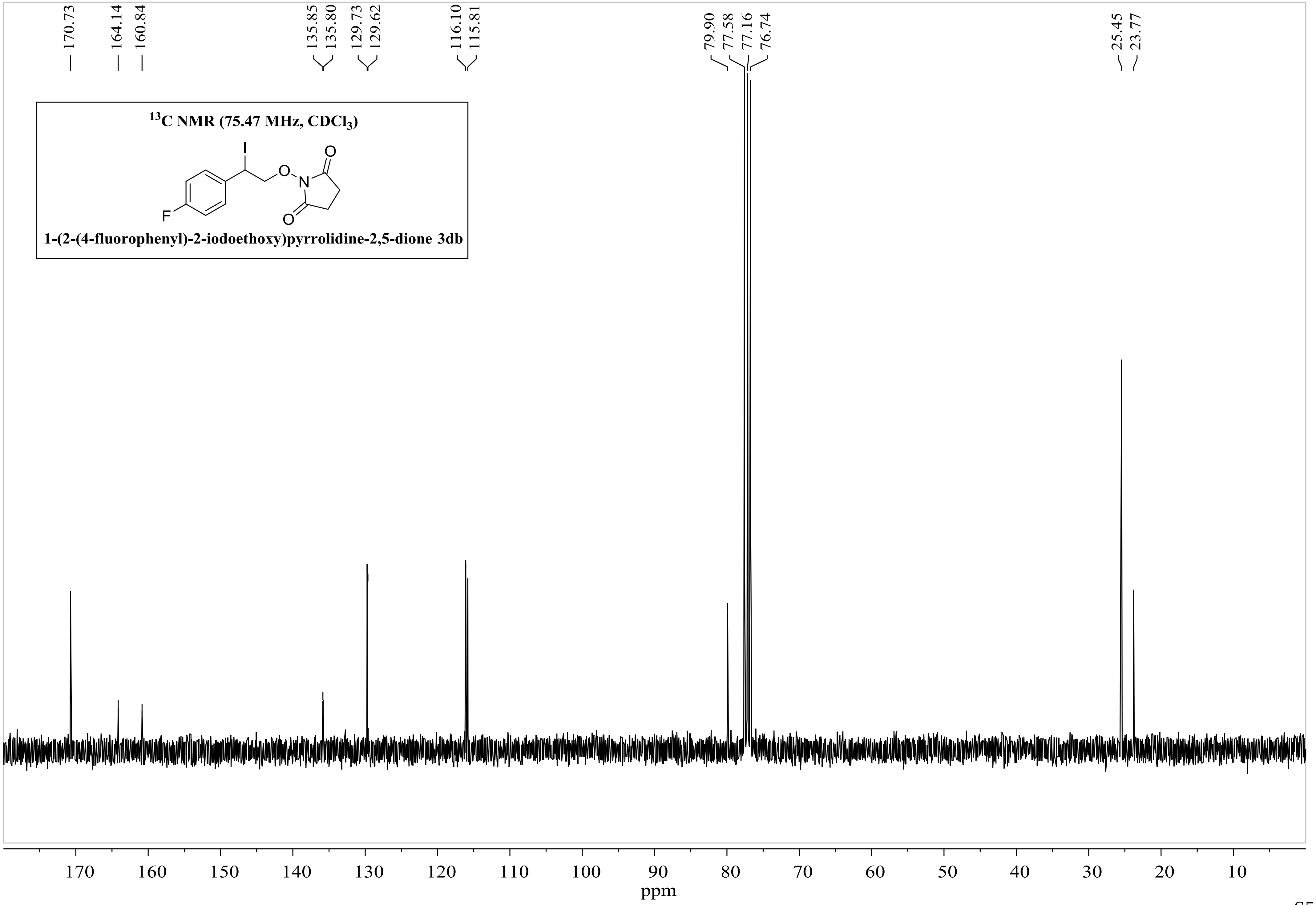
170.73
164.14
160.84
135.85
135.80
129.73
129.62
116.10
115.81
79.90
77.58
77.16
76.74
25.45
23.77
13C NMR (75.47 MHz, CDCl3)
I
O
O
N
O
F
1-(2-(4-fluorophenyl)-2-iodoethoxy)pyrrolidine-2,5-dione 3db
170
160
150
140
130
120
110
100
90
80
70
60
50
40
30
20
10
ppm

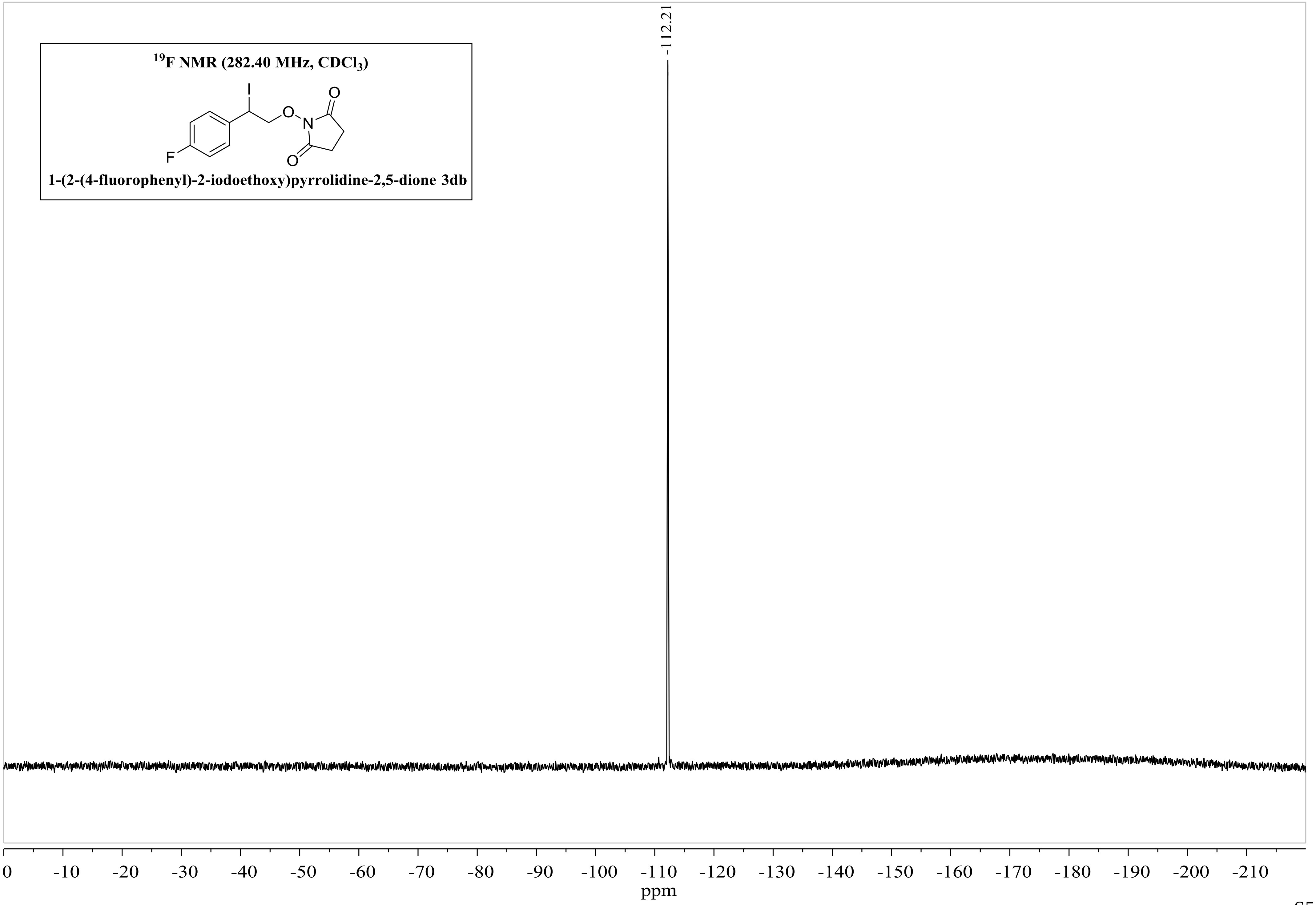

^{19}F NMR (282.40 MHz, CDCl$_3$)
I
O
O
N
F
O
1-(2-(4-fluorophenyl)-2-iodoethoxy)pyrrolidine-2,5-dione 3db
-112.21
0 -10 -20 -30 -40 -50 -60 -70 -80 -90 -100 -110 -120 -130 -140 -150 -160 -170 -180 -190 -200 -210
ppm

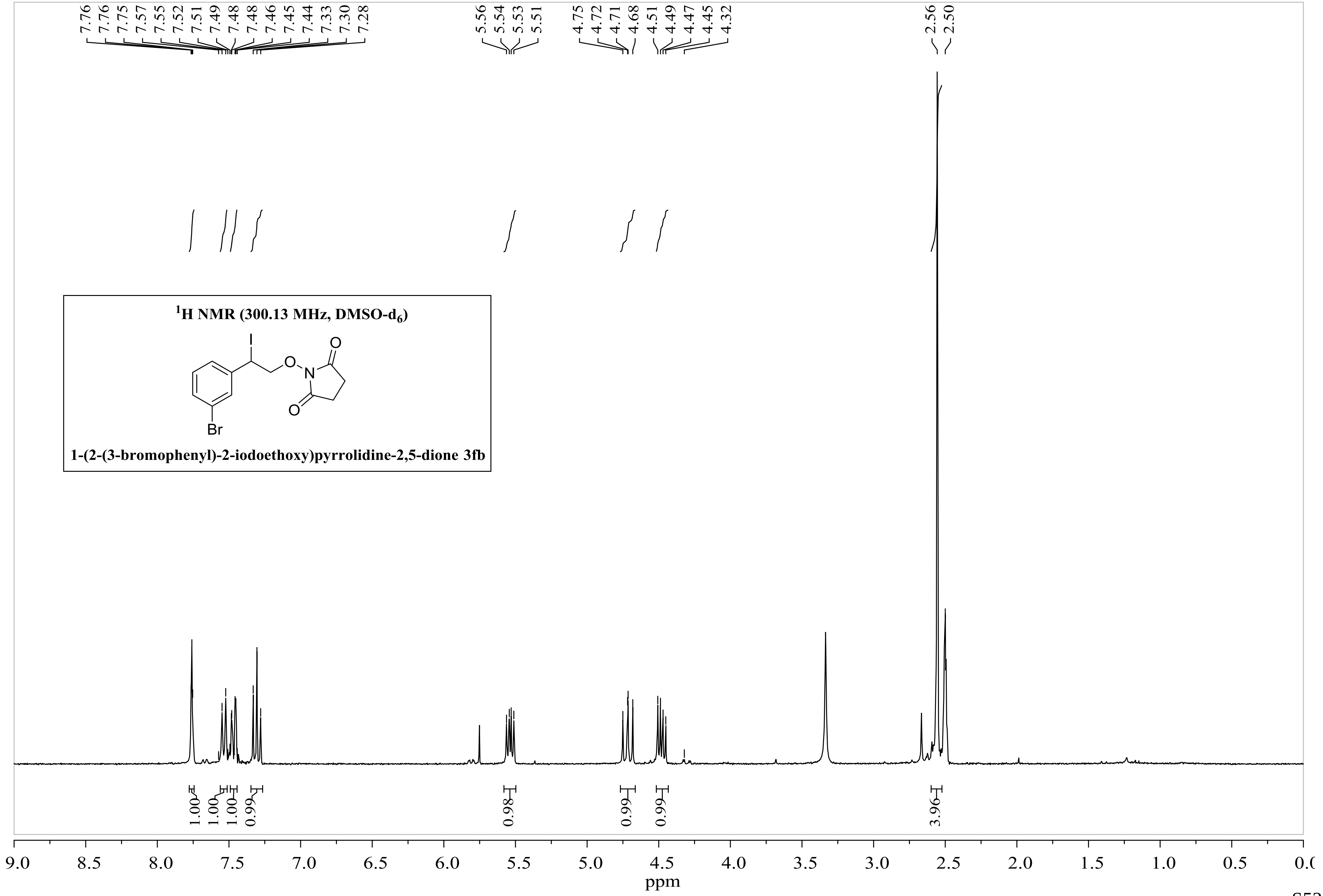

7.76
7.76
7.75
7.57
7.55
7.52
7.51
7.49
7.48
7.48
7.46
7.45
7.44
7.33
7.30
7.28
5.56
5.54
5.53
5.51
4.75
4.72
4.71
4.68
4.51
4.49
4.47
4.45
4.32
2.56
2.50
1H NMR (300.13 MHz, DMSO-d6)
I
O
O
N
O
O
Br
1-(2-(3-bromophenyl)-2-iodoethoxy)pyrrolidine-2,5-dione 3fb
1.00
1.00
1.00
0.99
0.98
0.99
0.99
3.96
9.0
8.5
8.0
7.5
7.0
6.5
6.0
5.5
5.0
4.5
4.0
3.5
3.0
2.5
2.0
1.5
1.0
0.5
ppm

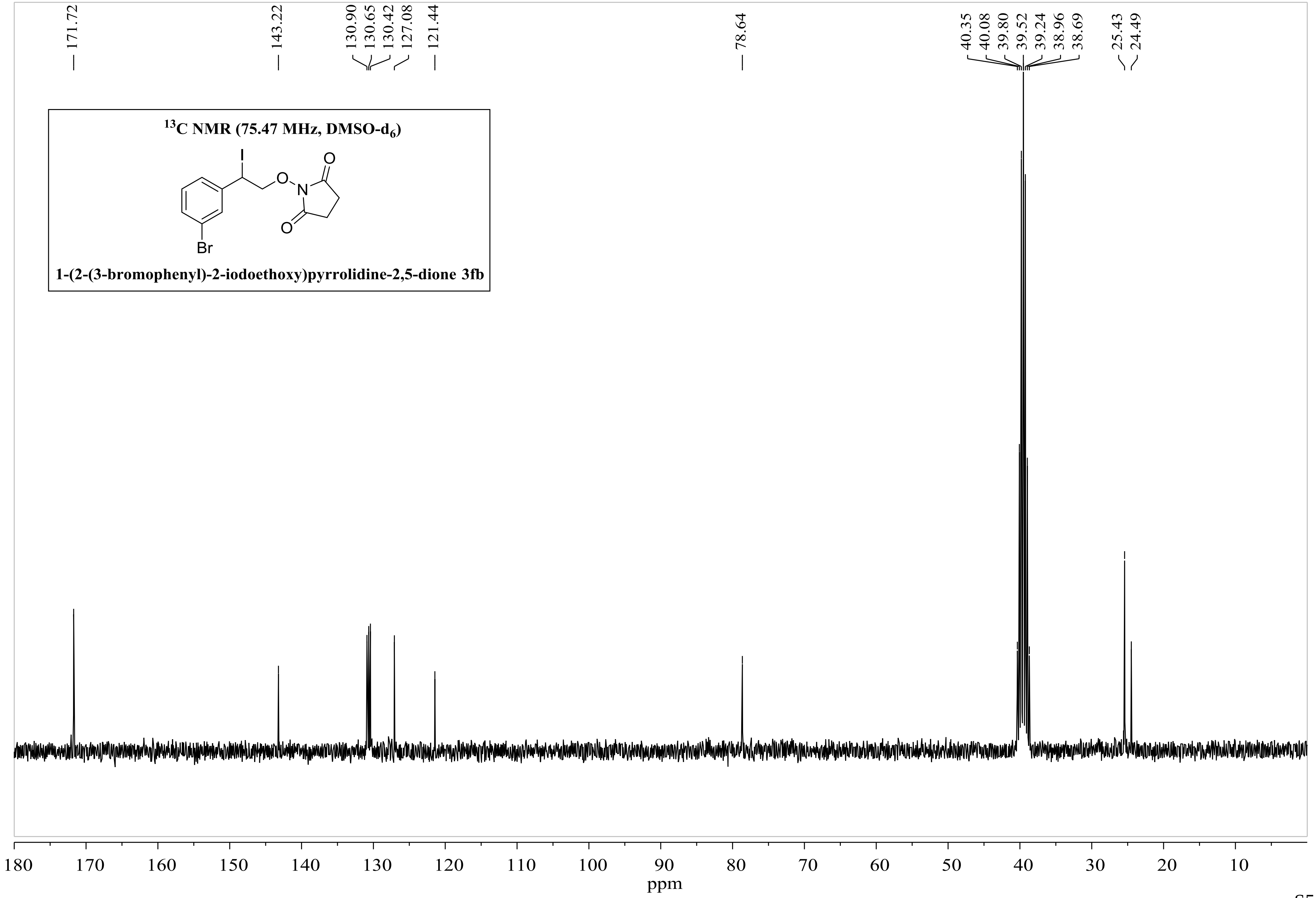

171.72
143.22
130.90
130.65
130.42
127.08
121.44
78.64
40.35
40.08
39.80
39.52
39.24
38.96
38.69
25.43
24.49
^{13}C NMR (75.47 MHz, DMSO-d_6)
I
O
O
N
O
Br
1-(2-(3-bromophenyl)-2-iodoethoxy)pyrrolidine-2,5-dione 3fb
180
170
160
150
140
130
120
110
100
90
80
70
60
50
40
30
20
10
ppm

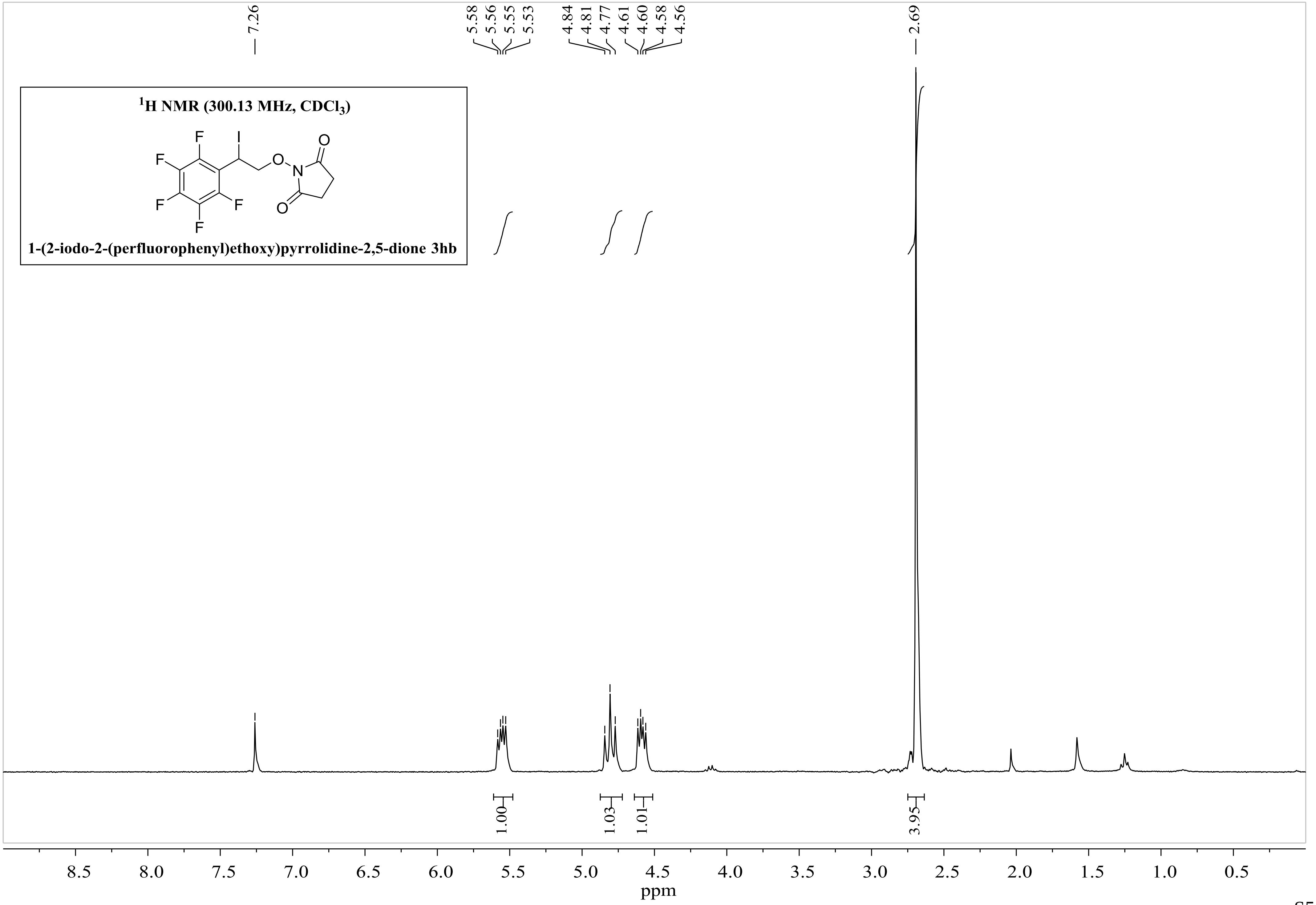

1H NMR (300.13 MHz, CDCl3)
1-(2-iodo-2-(perfluorophenyl)ethoxy)pyrrolidine-2,5-dione 3hb
7.26
5.58 5.56 5.55 5.53
4.84 4.81 4.77 4.61 4.60 4.58 4.56
2.69
1.00
1.03
1.01
3.95
8.5 8.0 7.5 7.0 6.5 6.0 5.5 5.0 4.5 4.0 3.5 3.0 2.5 2.0 1.5 1.0 0.5
ppm

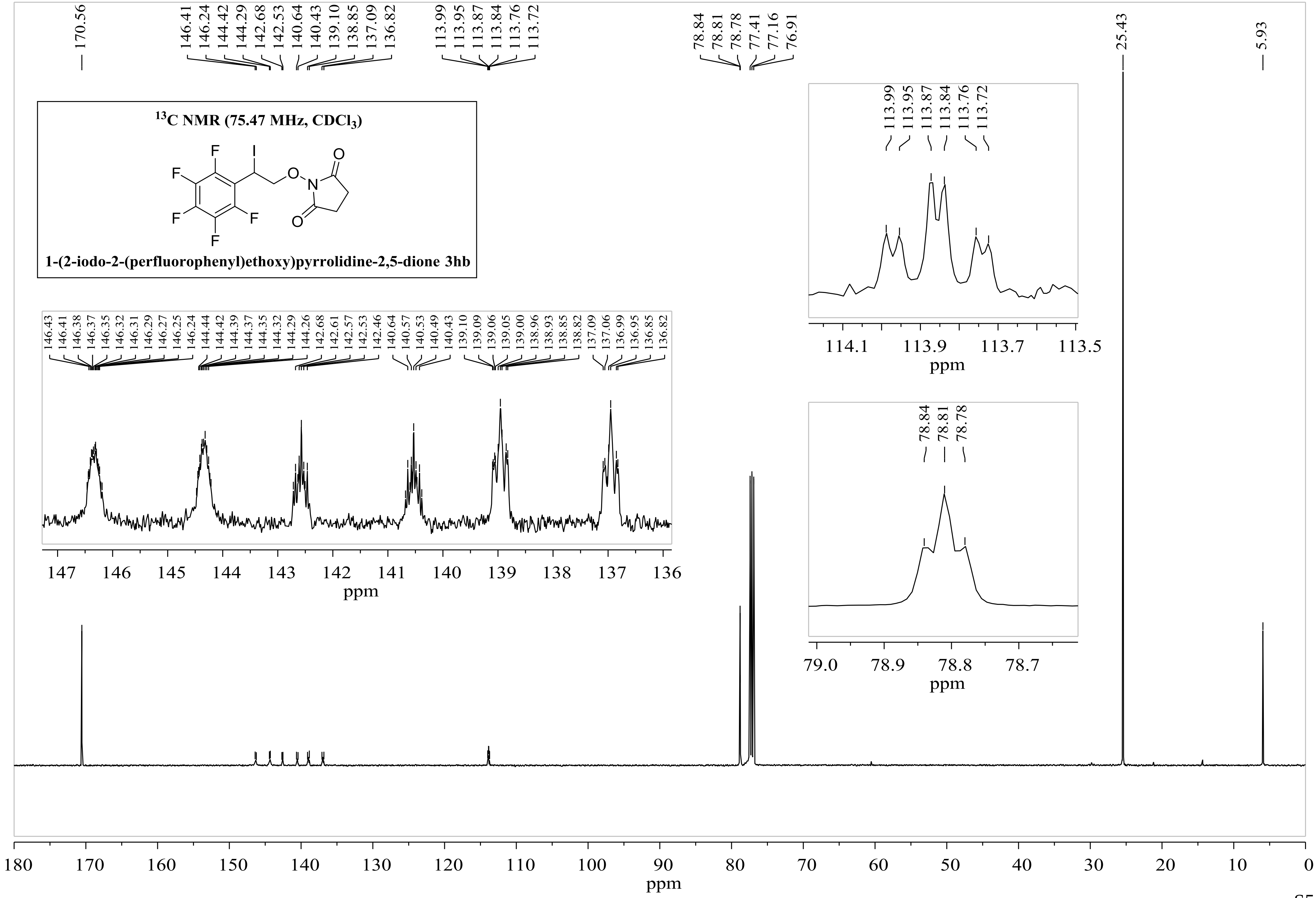

^{13}C NMR (75.47 MHz, CDCl$_3$)
1-(2-iodo-2-(perfluorophenyl)ethoxy)pyrrolidine-2,5-dione 3hb
170.56
146.41
146.24
144.42
144.29
142.68
142.53
140.64
140.43
139.10
138.85
137.09
136.82
113.99
113.95
113.87
113.84
113.76
113.72
78.84
78.81
78.78
77.41
77.16
76.91
25.43
5.93
ppm

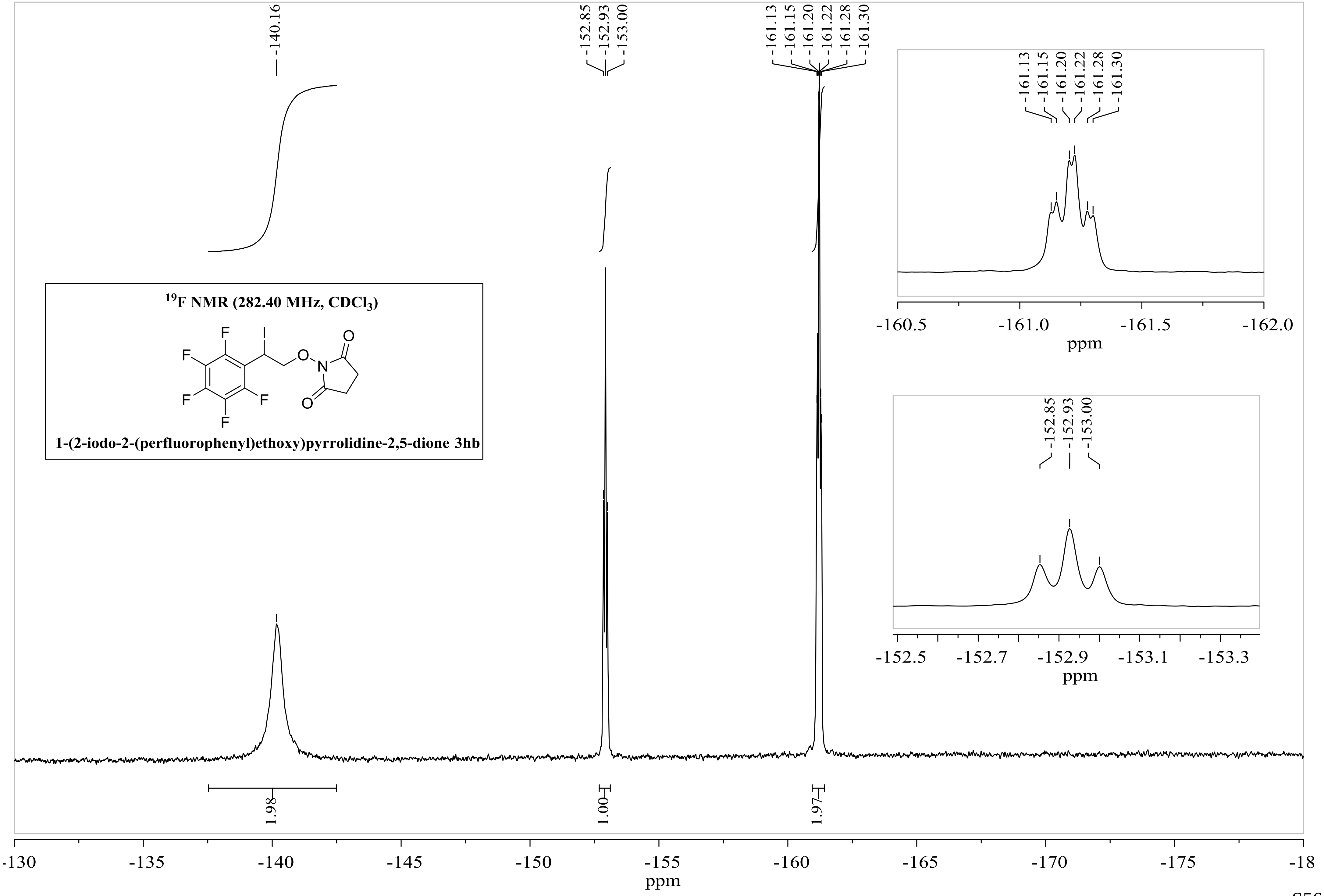
-140.16
-152.85
-152.93
-153.00
-161.13
-161.15
-161.20
-161.22
-161.28
-161.30
^{19}F NMR (282.40 MHz, $CDCl_3$)
F
F
F
F
F
I
O
N
O
O
1-(2-iodo-2-(perfluorophenyl)ethoxy)pyrrolidine-2,5-dione 3hb
1.98
1.00
1.97
-130
-135
-140
-145
-150
-155
-160
-165
-170
-175
ppm
-160.5
-161.0
-161.5
-162.0
ppm
-152.5
-152.7
-152.9
-153.1
-153.3
ppm

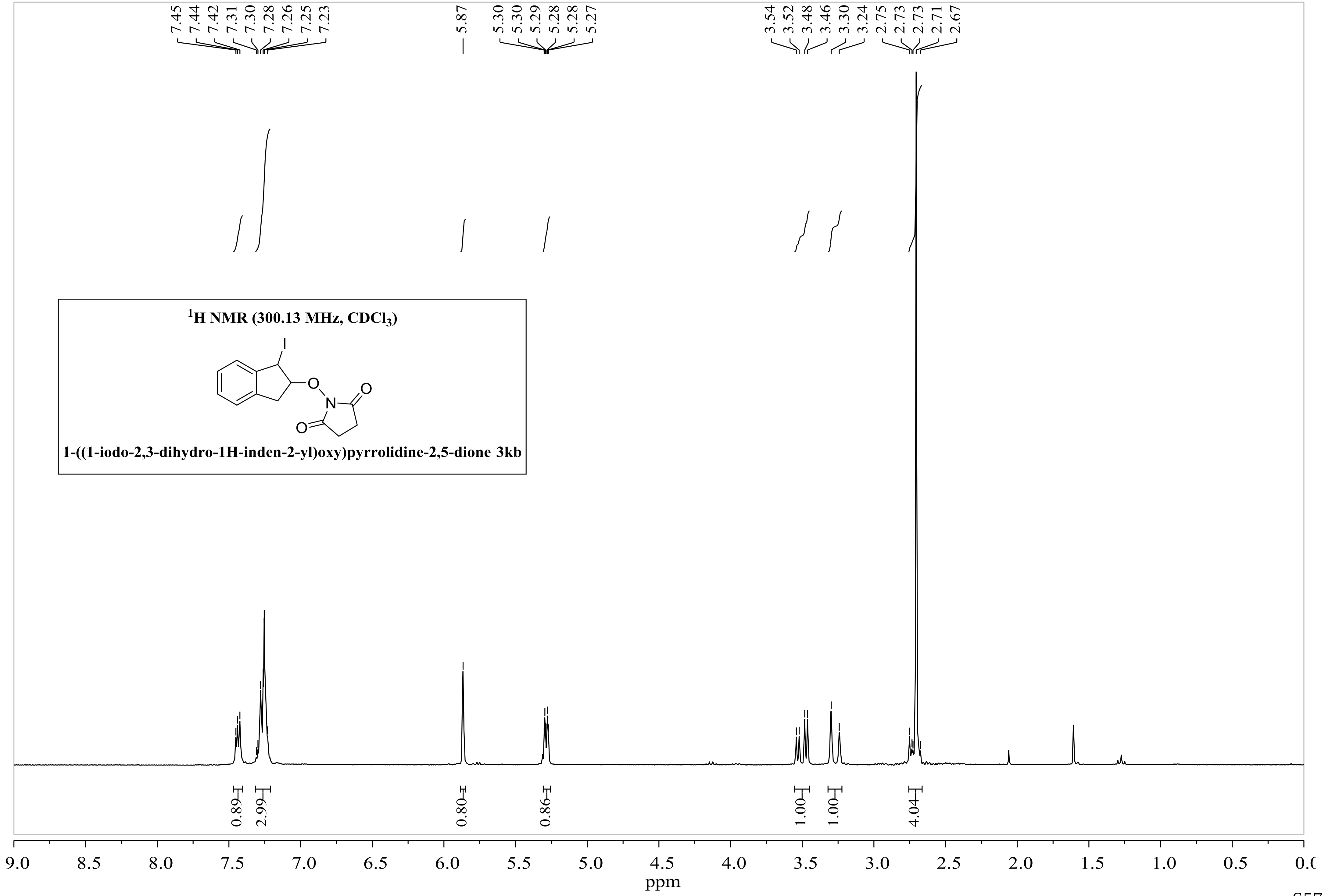
7.45 7.44 7.42 7.31 7.30 7.28 7.26 7.25 7.23
5.87
5.30 5.30 5.29 5.28 5.28 5.27
3.54 3.52 3.48 3.46 3.30 3.24 2.75 2.73 2.73 2.71 2.67
^{1}H NMR (300.13 MHz, CDCl$_3$)
I
O
N
O
O
1-((1-iodo-2,3-dihydro-1H-inden-2-yl)oxy)pyrrolidine-2,5-dione 3kb
0.89 2.99 0.80 0.86 1.00 1.00 4.04
9.0 8.5 8.0 7.5 7.0 6.5 6.0 5.5 5.0 4.5 4.0 3.5 3.0 2.5 2.0 1.5 1.0 0.5 0.0
ppm

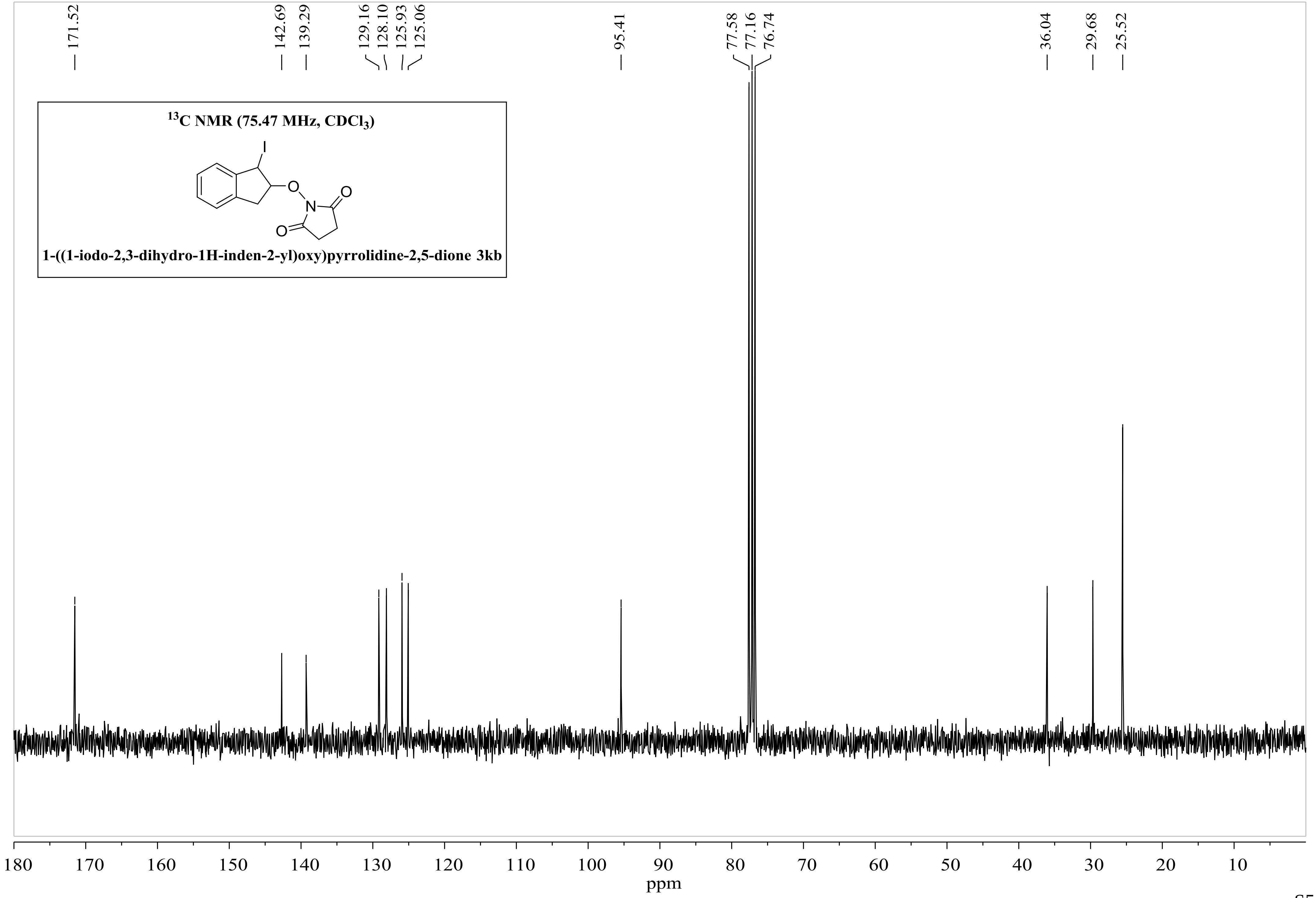
171.52
142.69
139.29
129.16
128.10
125.93
125.06
95.41
77.58
77.16
76.74
36.04
29.68
25.52
13C NMR (75.47 MHz, CDCl3)
I
O
N
O
O
1-((1-iodo-2,3-dihydro-1H-inden-2-yl)oxy)pyrrolidine-2,5-dione 3kb
180
170
160
150
140
130
120
110
100
90
80
70
60
50
40
30
20
10
ppm

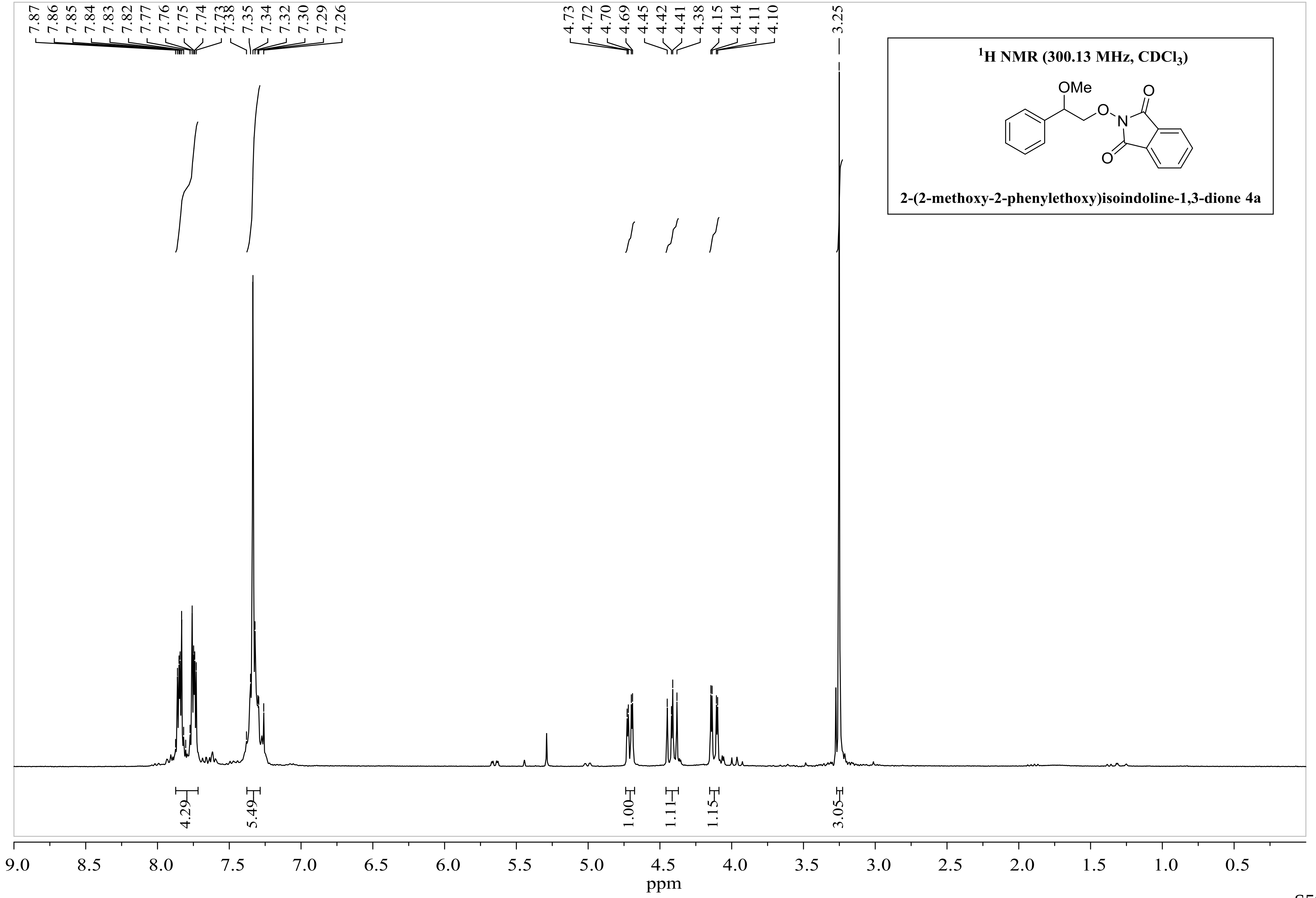

7.87 7.86 7.85 7.84 7.83 7.82 7.77 7.76 7.75 7.74 7.73 7.38 7.35 7.34 7.32 7.30 7.29 7.26
4.73 4.72 4.70 4.69 4.45 4.42 4.41 4.38 4.15 4.14 4.11 4.10
3.25
¹H NMR (300.13 MHz, CDCl3)
OMe
O
N
2-(2-methoxy-2-phenylethoxy)isoindoline-1,3-dione 4a
4.29
5.49
1.00
1.11
1.15
3.05
9.0 8.5 8.0 7.5 7.0 6.5 6.0 5.5 5.0 4.5 4.0 3.5 3.0 2.5 2.0 1.5 1.0 0.5
ppm

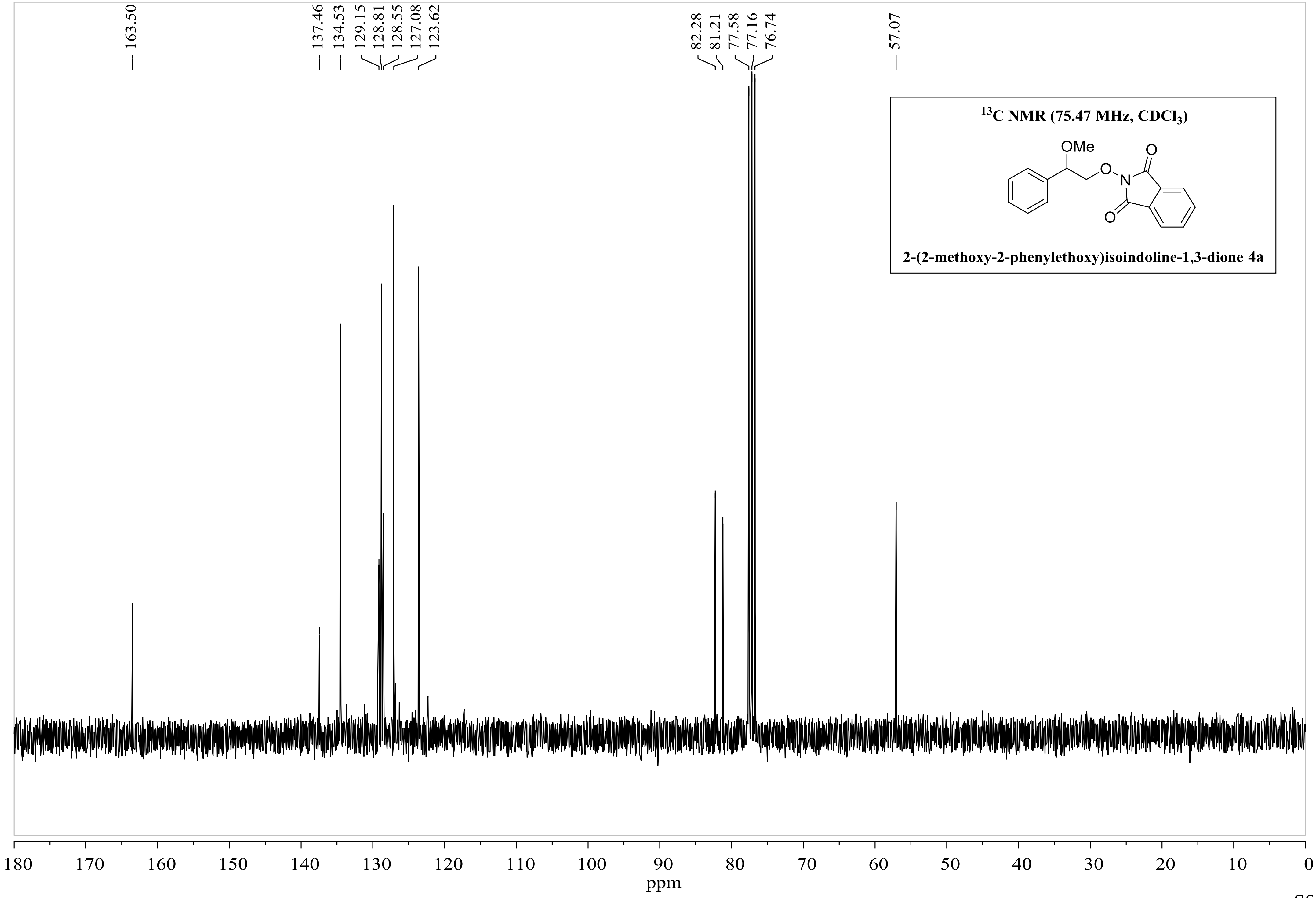
163.50
137.46
134.53
129.15
128.81
128.55
127.08
123.62
82.28
81.21
77.58
77.16
76.74
57.07
13C NMR (75.47 MHz, CDCl3)
OMe
O
N
O
O
2-(2-methoxy-2-phenylethoxy)isoindoline-1,3-dione 4a
180 170 160 150 140 130 120 110 100 90 80 70 60 50 40 30 20 10 0
ppm

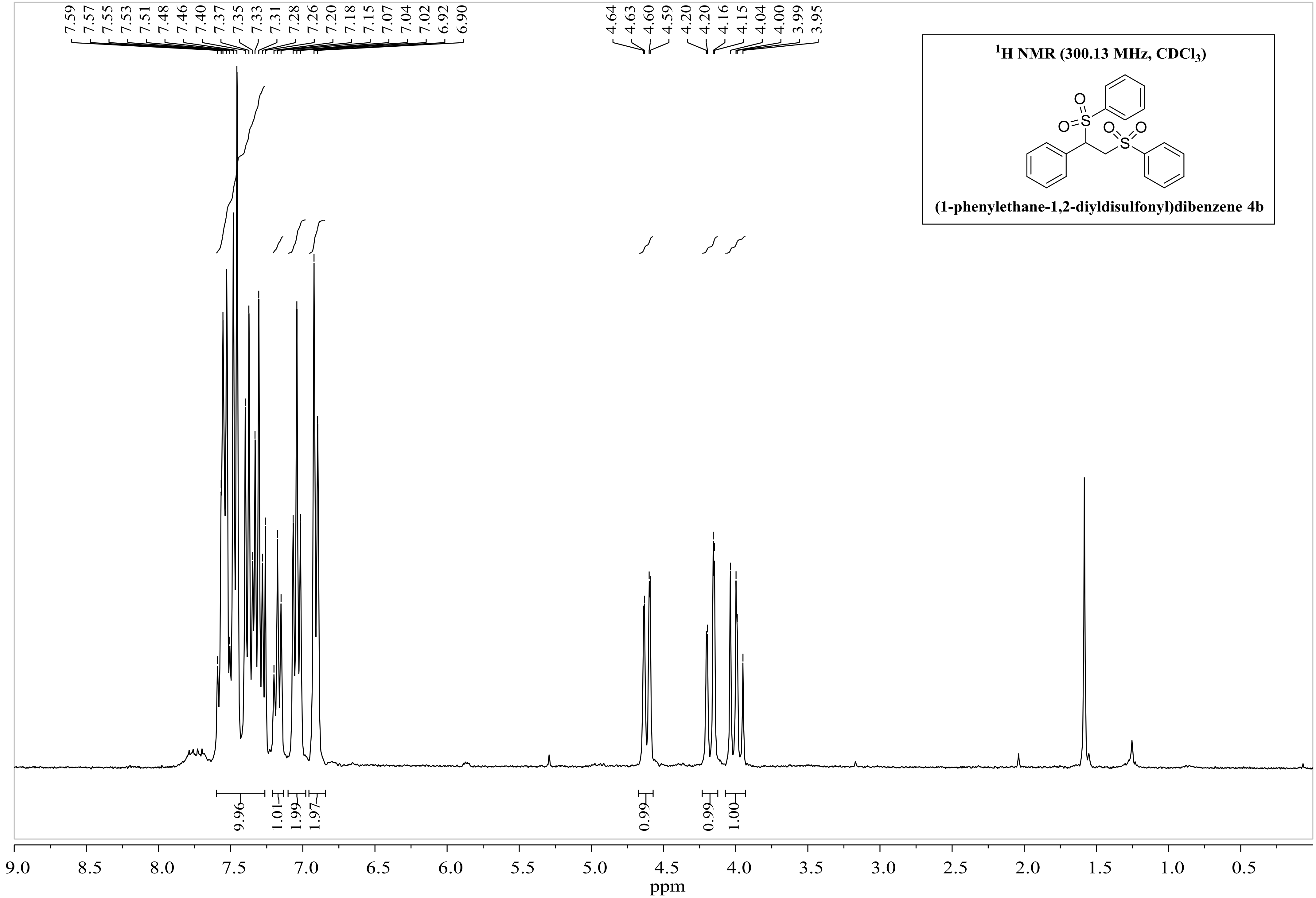

7.59 7.57 7.55 7.53 7.51 7.48 7.46 7.40 7.37 7.35 7.33 7.31 7.28 7.26 7.20 7.18 7.15 7.07 7.04 7.02 6.92 6.90
4.64 4.63 4.60 4.59 4.20 4.20 4.16 4.15 4.04 4.00 3.99 3.95
1H NMR (300.13 MHz, CDCl3)
(1-phenylethane-1,2-diyldisulfonyl)dibenzene 4b
9.96 1.01 1.99 1.97 0.99 0.99 1.00
9.0 8.5 8.0 7.5 7.0 6.5 6.0 5.5 5.0 4.5 4.0 3.5 3.0 2.5 2.0 1.5 1.0 0.5
ppm

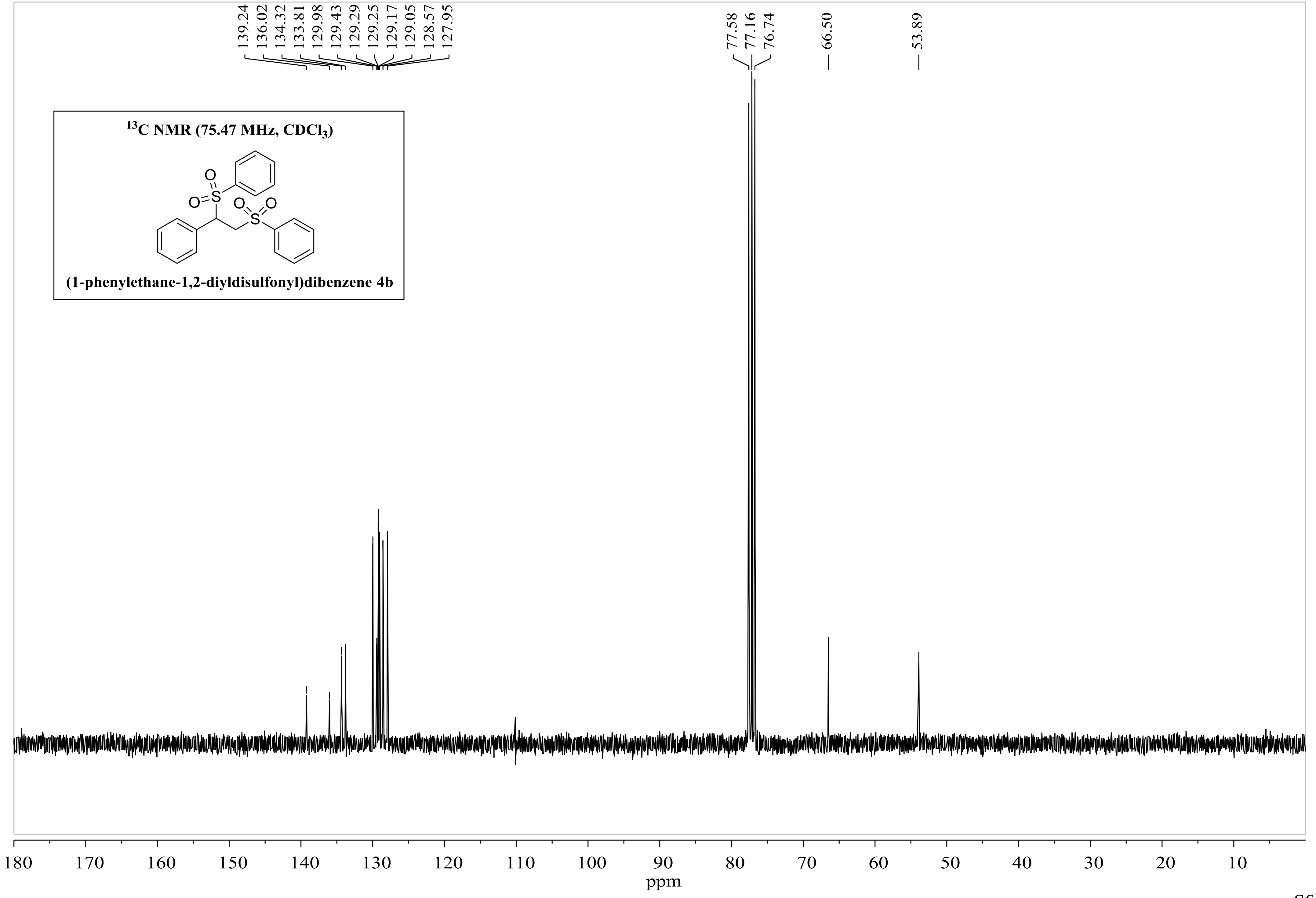
139.24
136.02
134.32
133.81
129.98
129.43
129.29
129.25
129.17
129.05
128.57
127.95
77.58
77.16
76.74
66.50
53.89
^{13}C NMR (75.47 MHz, $CDCl_3$)
(1-phenylethane-1,2-diyldisulfonyl)dibenzene 4b
180 170 160 150 140 130 120 110 100 90 80 70 60 50 40 30 20 10
ppm

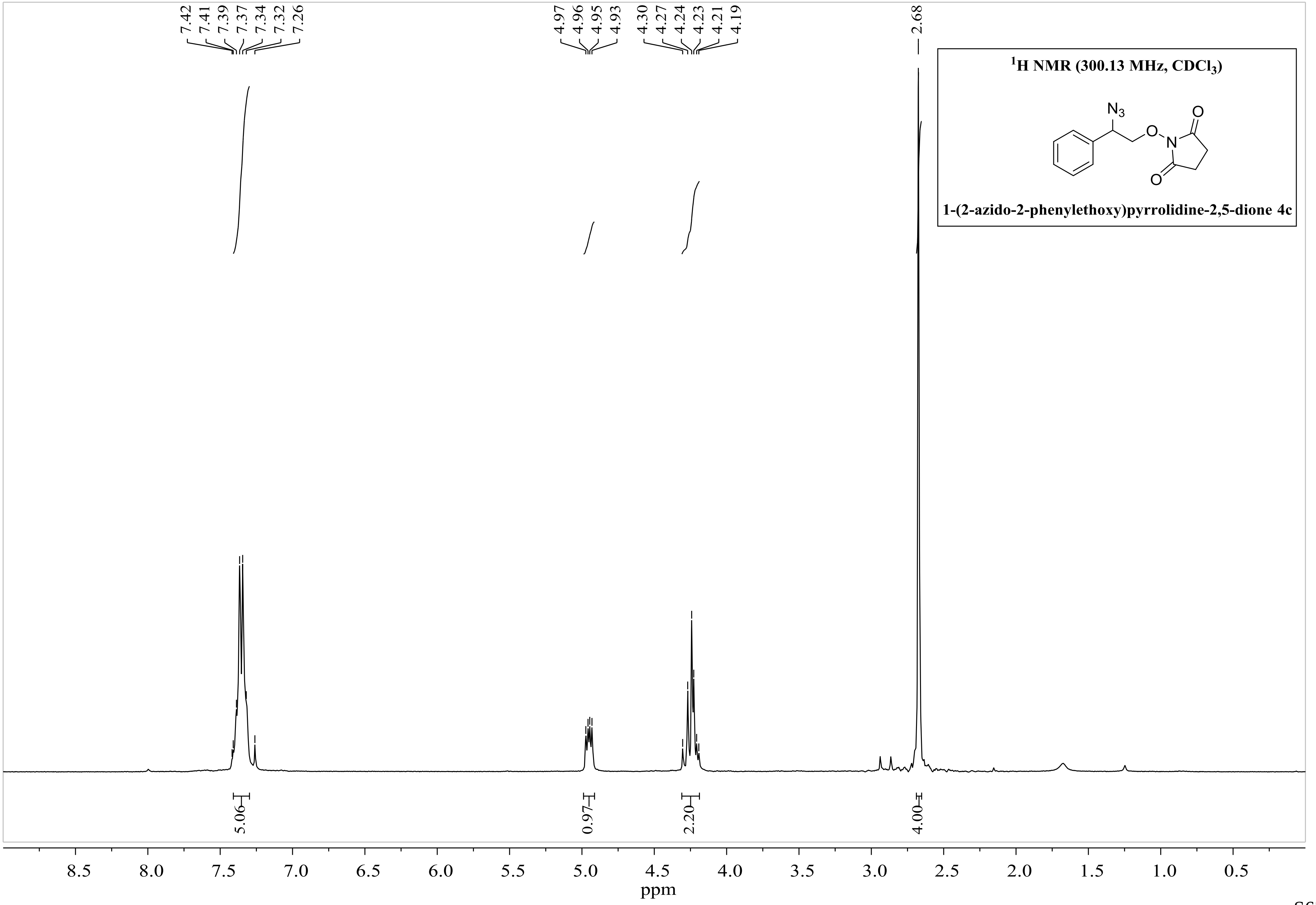

7.42
7.41
7.39
7.37
7.34
7.32
7.26
4.97
4.96
4.95
4.93
4.30
4.27
4.24
4.23
4.21
4.19
2.68
1H NMR (300.13 MHz, CDCl3)
N3
O
N
O
O
O
1-(2-azido-2-phenylethoxy)pyrrolidine-2,5-dione 4c
5.06
0.97
2.20
4.00
8.5
8.0
7.5
7.0
6.5
6.0
5.5
5.0
4.5
4.0
3.5
3.0
2.5
2.0
1.5
1.0
0.5
ppm

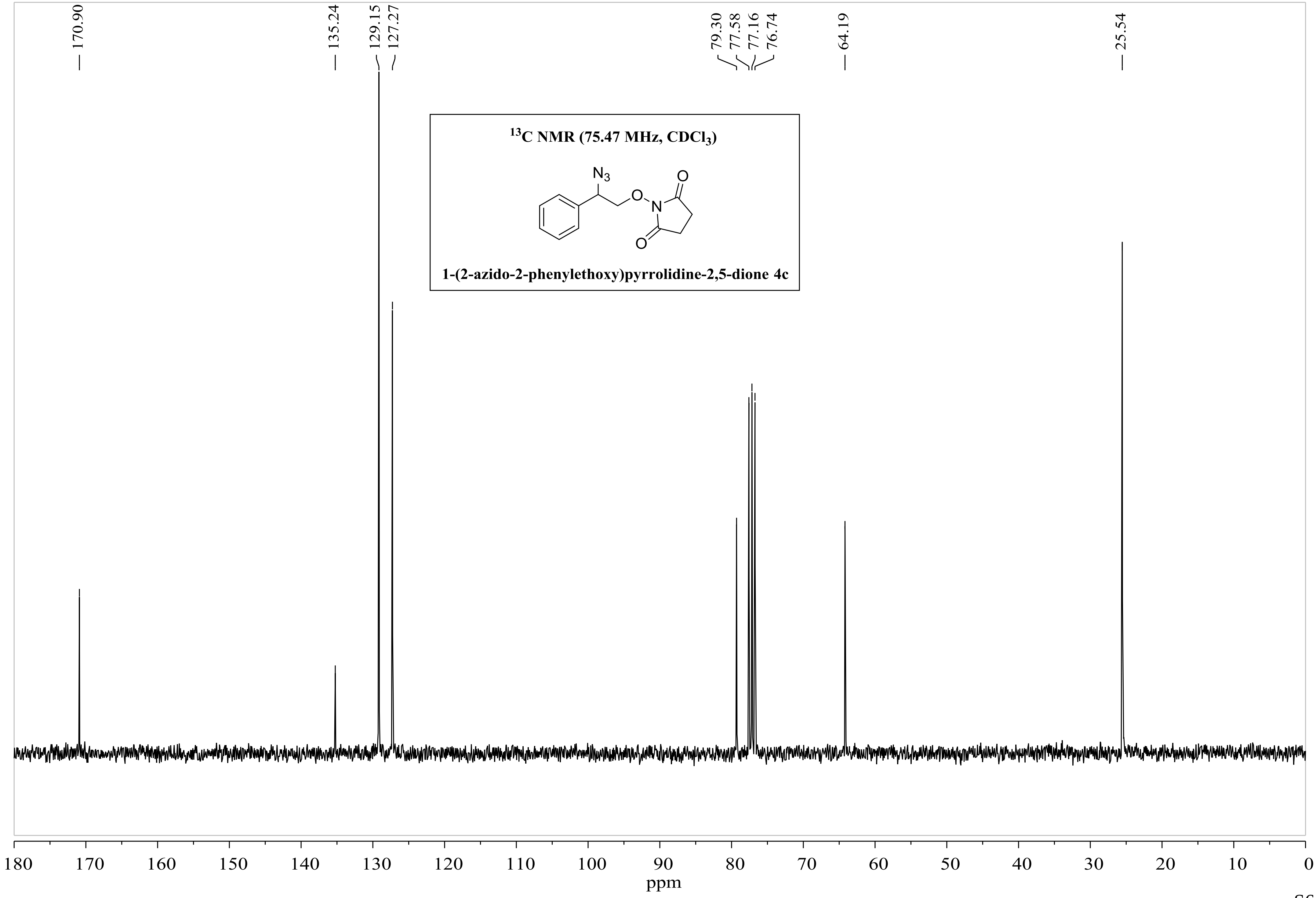
170.90
135.24
129.15
127.27
79.30
77.58
77.16
76.74
64.19
25.54
^{13}C NMR (75.47 MHz, CDCl$_3$)
N$_3$
O
N
O
O
1-(2-azido-2-phenylethoxy)pyrrolidine-2,5-dione 4c
180
170
160
150
140
130
120
110
100
90
80
70
60
50
40
30
20
10
0
ppm

HRMS (ESI) of 2-(2-iodo-2-(o-tolyl)ethoxy)isoindoline-1,3-dione 3ga

Display Report

Analysis Info

Analysis Name	D:\Data\Kolotyrkina\2018\Pavel'ev\0321018.d	Acquisition Date	21.03.2018 17:43:26
Method	tune_low.m	Operator	BDAL@DE
Sample Name	/TERN NN-16	Instrument / Ser#	micrOTOF 10248
Comment	C17H14INO3 mH 408.0091 clb added		

Acquisition Parameter

Source Type	ESI	Ion Polarity	Positive	Set Nebulizer	0.4 Bar
Focus	Not active			Set Dry Heater	180 °C
Scan Begin	50 m/z	Set Capillary	4500 V	Set Dry Gas	4.0 l/min
Scan End	3000 m/z	Set End Plate Offset	-500 V	Set Divert Valve	Waste

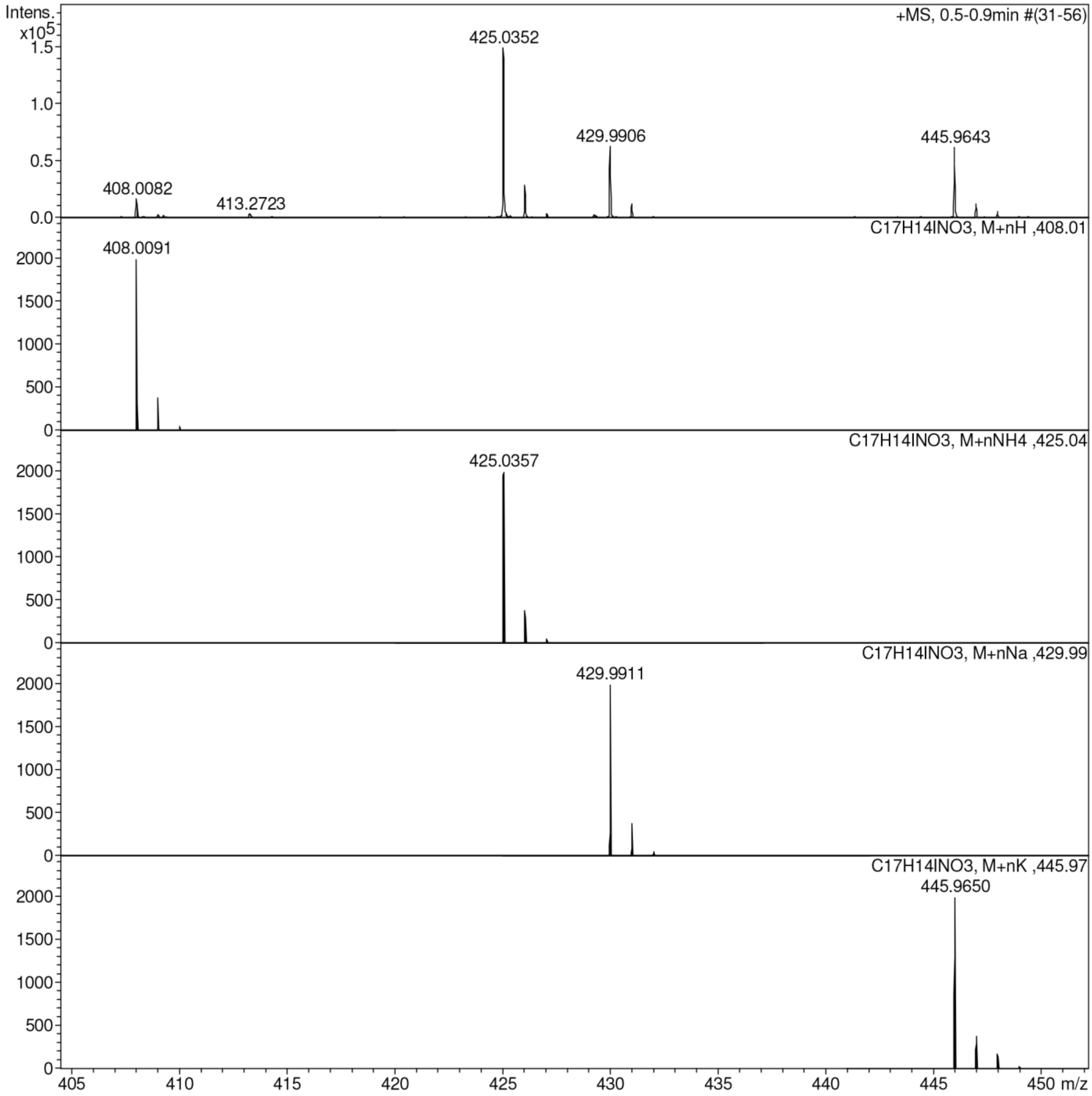

HRMS (ESI) of 2-((1-iodo-1-phenylpropan-2-yl)oxy)isoindoline-1,3-dione 3ia

Display Report

Analysis Info

Analysis Name	D:\Data\Kolotyrkina\2018\Pavel'ev\0321017.d	Acquisition Date	21.03.2018 17:29:33
Method	tune_low.m	Operator	BDAL@DE
Sample Name	/TERN C-800	Instrument / Ser#	micrOTOF 10248
Comment	C17H14INO3 mH 408.0091 clb added		

Acquisition Parameter

Source Type	ESI	Ion Polarity	Positive	Set Nebulizer	0.4 Bar
Focus	Not active			Set Dry Heater	180 °C
Scan Begin	50 m/z	Set Capillary	4500 V	Set Dry Gas	4.0 l/min
Scan End	3000 m/z	Set End Plate Offset	-500 V	Set Divert Valve	Waste

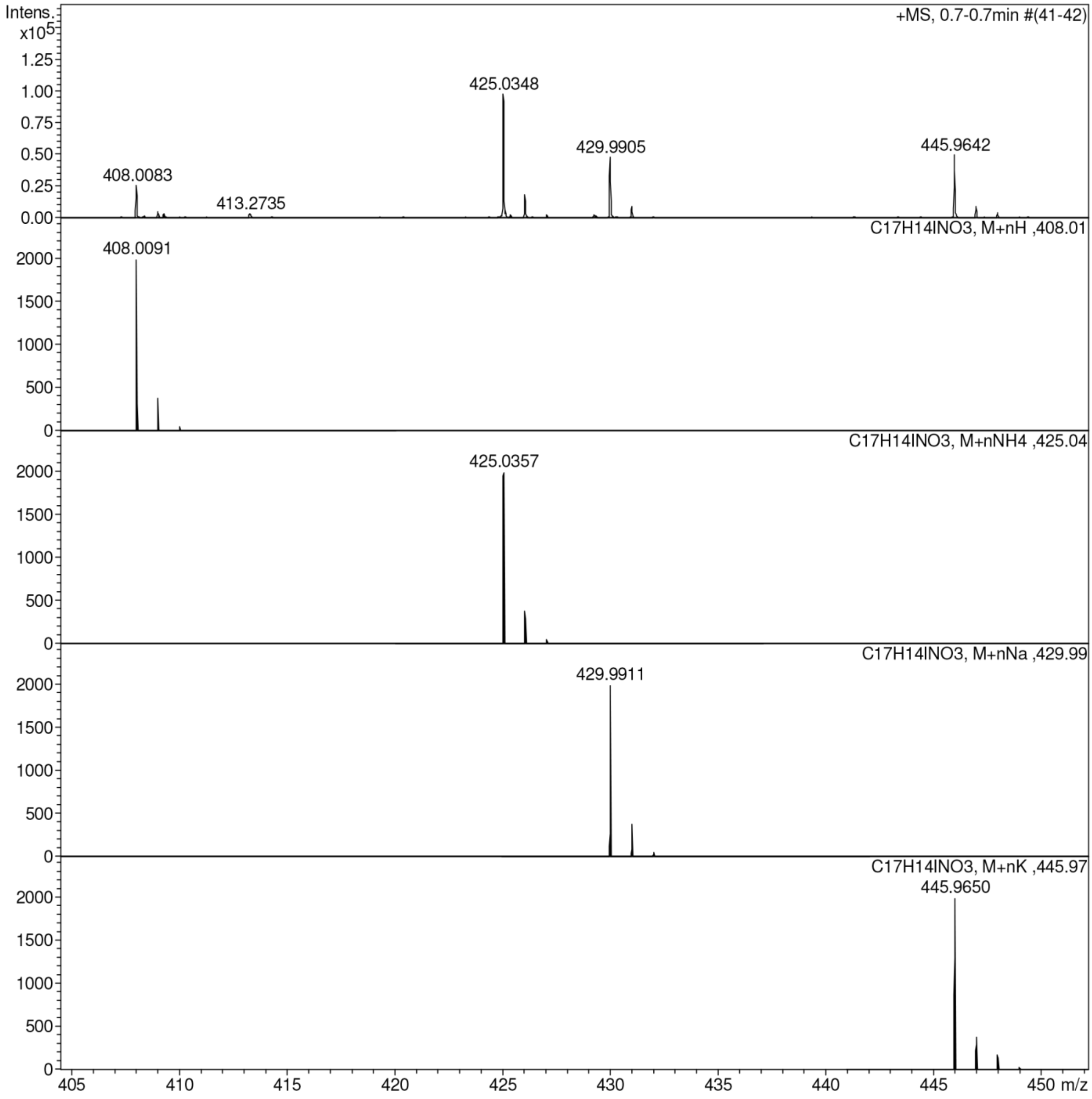

Bruker Compass DataAnalysis 4.0 printed: 21.03.2018 17:33:36 Page 1 of 1

HRMS (ESI) of 1-(2-(4-chlorophenyl)-2-iodoethoxy)pyrrolidine-2,5-dione 3cb

Display Report

Analysis Info

Analysis Name	D:\Data\Kolotyrkina\2018\Pavel'ev\0321021.d	Acquisition Date	21.03.2018 17:59:24
Method	tune_low.m	Operator	BDAL@DE
Sample Name	/TERN NN-7	Instrument / Ser#	micrOTOF 10248
Comment	C12H11ClINO3 mH 379.9544 clb added		

Acquisition Parameter

Source Type	ESI	Ion Polarity	Positive	Set Nebulizer	0.4 Bar
Focus	Not active			Set Dry Heater	180 °C
Scan Begin	50 m/z	Set Capillary	4500 V	Set Dry Gas	4.0 l/min
Scan End	3000 m/z	Set End Plate Offset	-500 V	Set Divert Valve	Waste

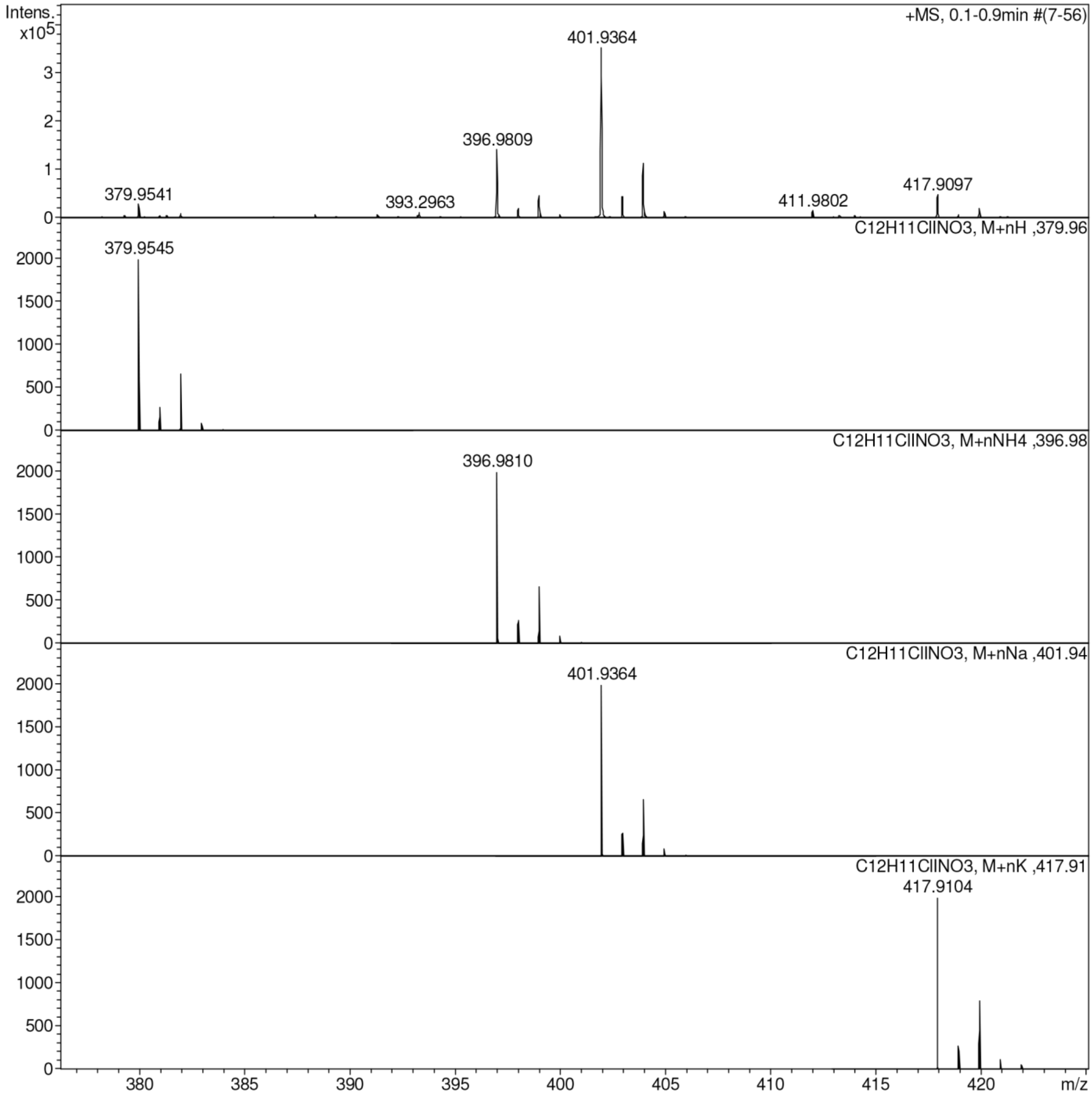

HRMS (ESI) of 2-(2-methoxy-2-phenylethoxy)isoindoline-1,3-dione 4a

Display Report

Analysis Info

Analysis Name	D:\Data\Kolotyrkina\2018\Pavel'ev\0515017.d	Acquisition Date	15.05.2018 17:14:10
Method	tune_50-1600.m	Operator	BDAL@DE
Sample Name	/TERN C891	Instrument / Ser#	micrOTOF 10248
Comment	C17H15NO4 mH 298.1073 clb added		

Acquisition Parameter

Source Type	ESI	Ion Polarity	Positive	Set Nebulizer	1.0 Bar
Focus	Not active			Set Dry Heater	200 °C
Scan Begin	50 m/z	Set Capillary	4500 V	Set Dry Gas	4.0 l/min
Scan End	1600 m/z	Set End Plate Offset	-500 V	Set Divert Valve	Waste

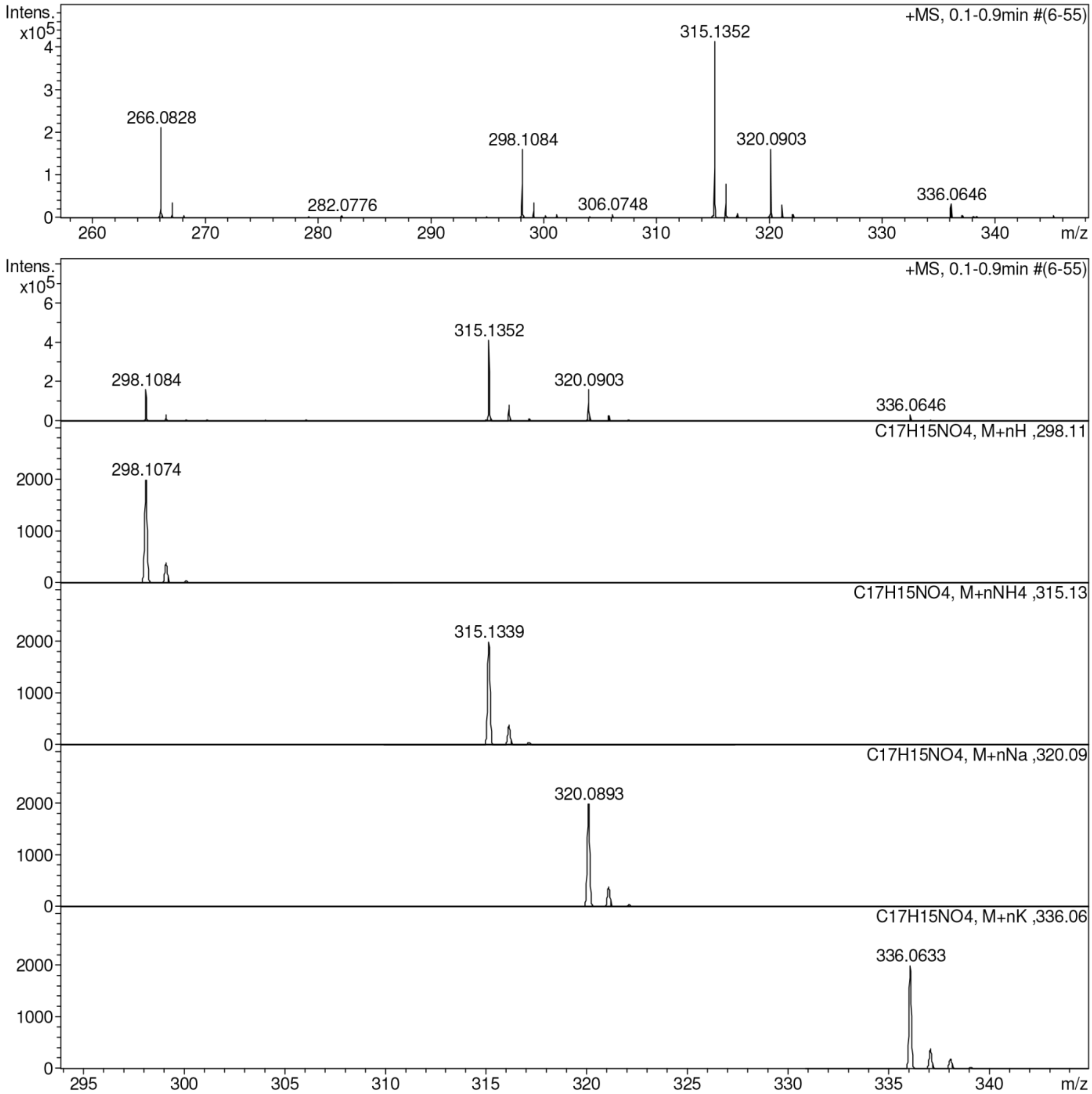

HRMS (ESI) of (1-phenylethane-1,2-diyldisulfonyl)dibenzene 4b

Display Report

Analysis Info

Analysis Name	D:\Data\Chizhov\Terentiev\Paveliev\c-711_&clblow.d	Acquisition Date	17.11.2017 17:41:57
Method	tune_low.m	Operator	BDAL@DE
Sample Name	/TERN C-711	Instrument / Ser#	micrOTOF 10248
Comment	CH3CN 100 %, dil. 200, calibrant added		

Acquisition Parameter

Source Type	ESI	Ion Polarity	Positive	Set Nebulizer	0.4 Bar
Focus	Not active			Set Dry Heater	180 °C
Scan Begin	50 m/z	Set Capillary	4500 V	Set Dry Gas	4.0 l/min
Scan End	3000 m/z	Set End Plate Offset	-500 V	Set Divert Valve	Waste

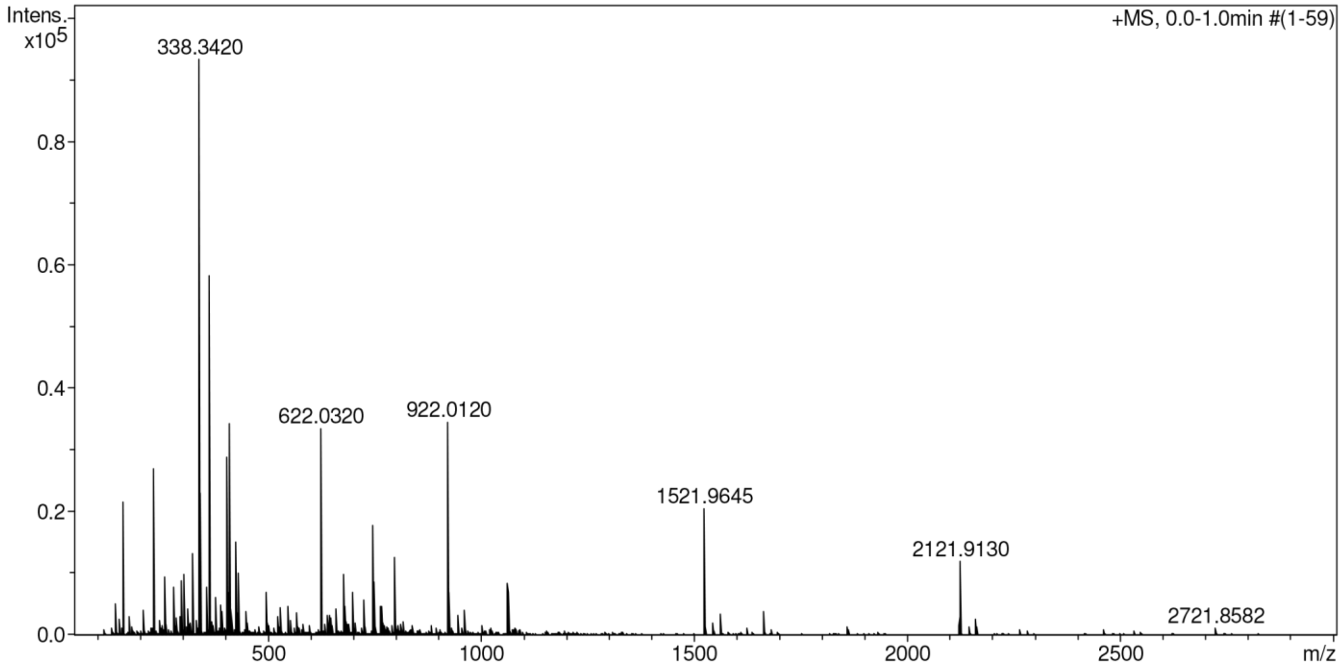

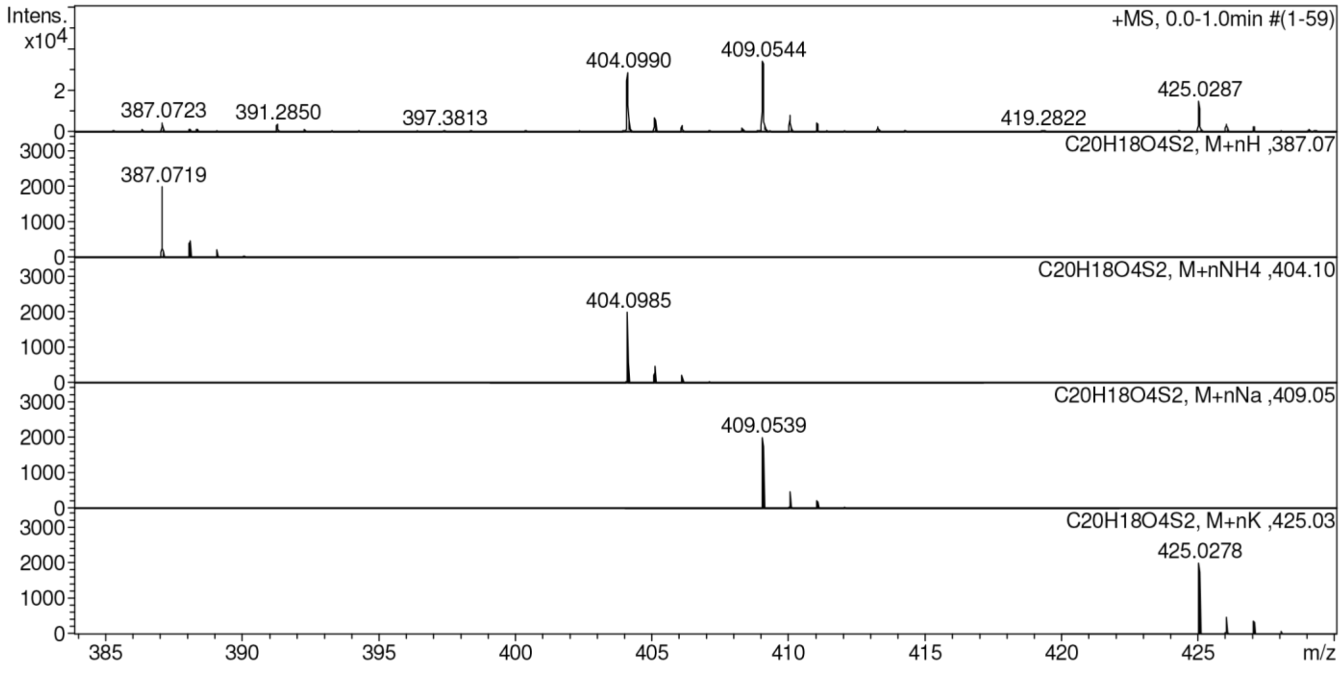

Bruker Compass DataAnalysis 4.0 printed: 17.11.2017 17:46:57 Page 1 of 1

1-(2-azido-2-phenylethoxy)pyrrolidine-2,5-dione 4c

Display Report

Analysis Info

Analysis Name	D:\Data\Kolotyrkina\2018\Pavel'ev\0517008.d	Acquisition Date	17.05.2018 10:28:44
Method	tune_50-1600.m	Operator	BDAL@DE
Sample Name	/TERN C889	Instrument / Ser#	micrOTOF 10248
Comment	C12H12N4O2 mH 261.0982 clb added		

Acquisition Parameter

Source Type	ESI	Ion Polarity	Positive	Set Nebulizer	1.0 Bar
Focus	Not active			Set Dry Heater	200 °C
Scan Begin	50 m/z	Set Capillary	4500 V	Set Dry Gas	4.0 l/min
Scan End	1600 m/z	Set End Plate Offset	-500 V	Set Divert Valve	Waste

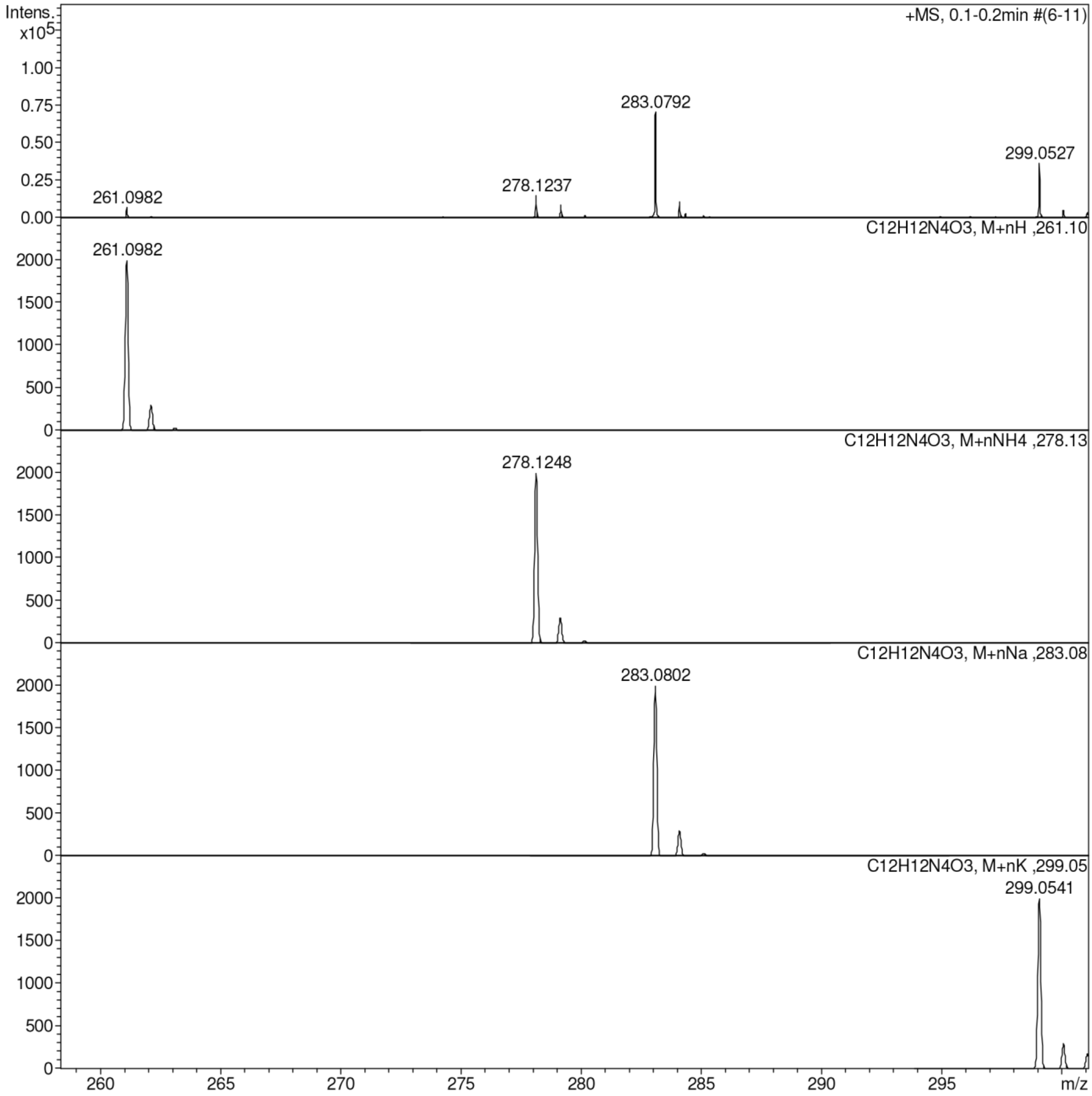

FT-IR spectrum of 2-(2-iodo-2-phenylethoxy)isoindoline-1,3-dione 3aa

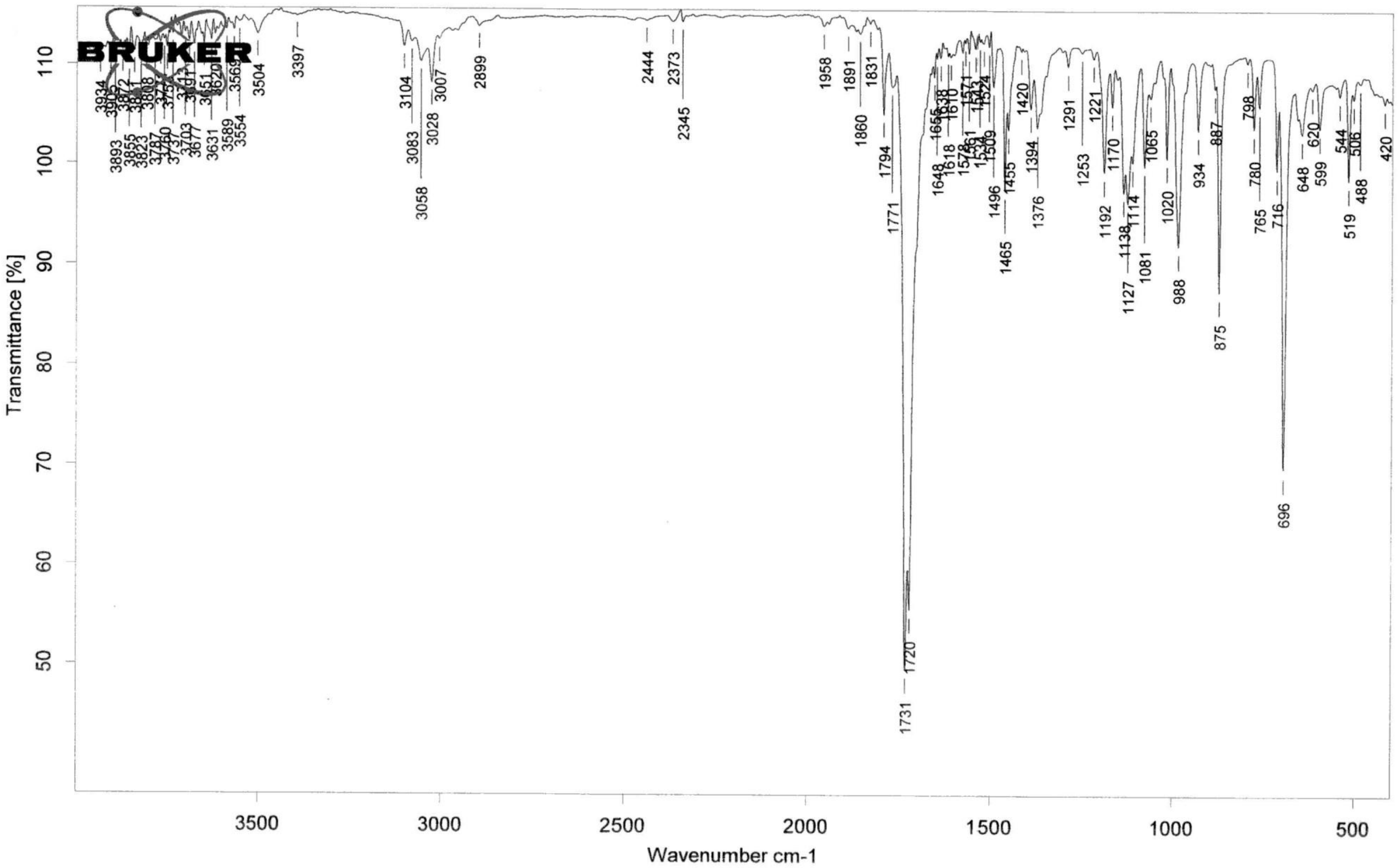

FT-IR (KBr pellet) spectrum of 2-(2-iodo-2-(*p*-tolyl)ethoxy)isoindoline-1,3-dione (mixture of regioisomers 4:1) 3ba

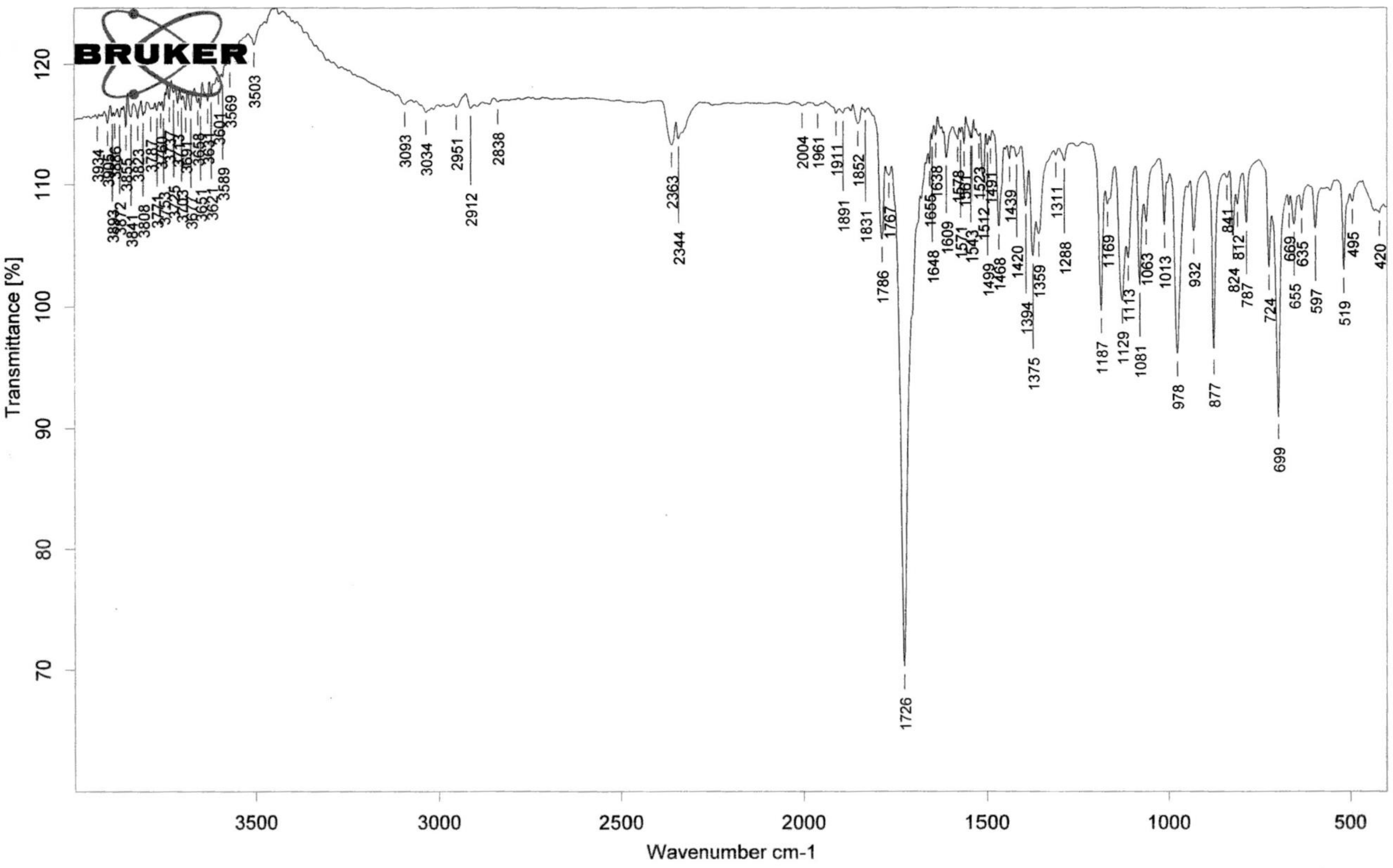

FT-IR (KBr pellet) spectrum of 2-(2-(4-chlorophenyl)-2-iodoethoxy)isoindoline-1,3-dione 3ca

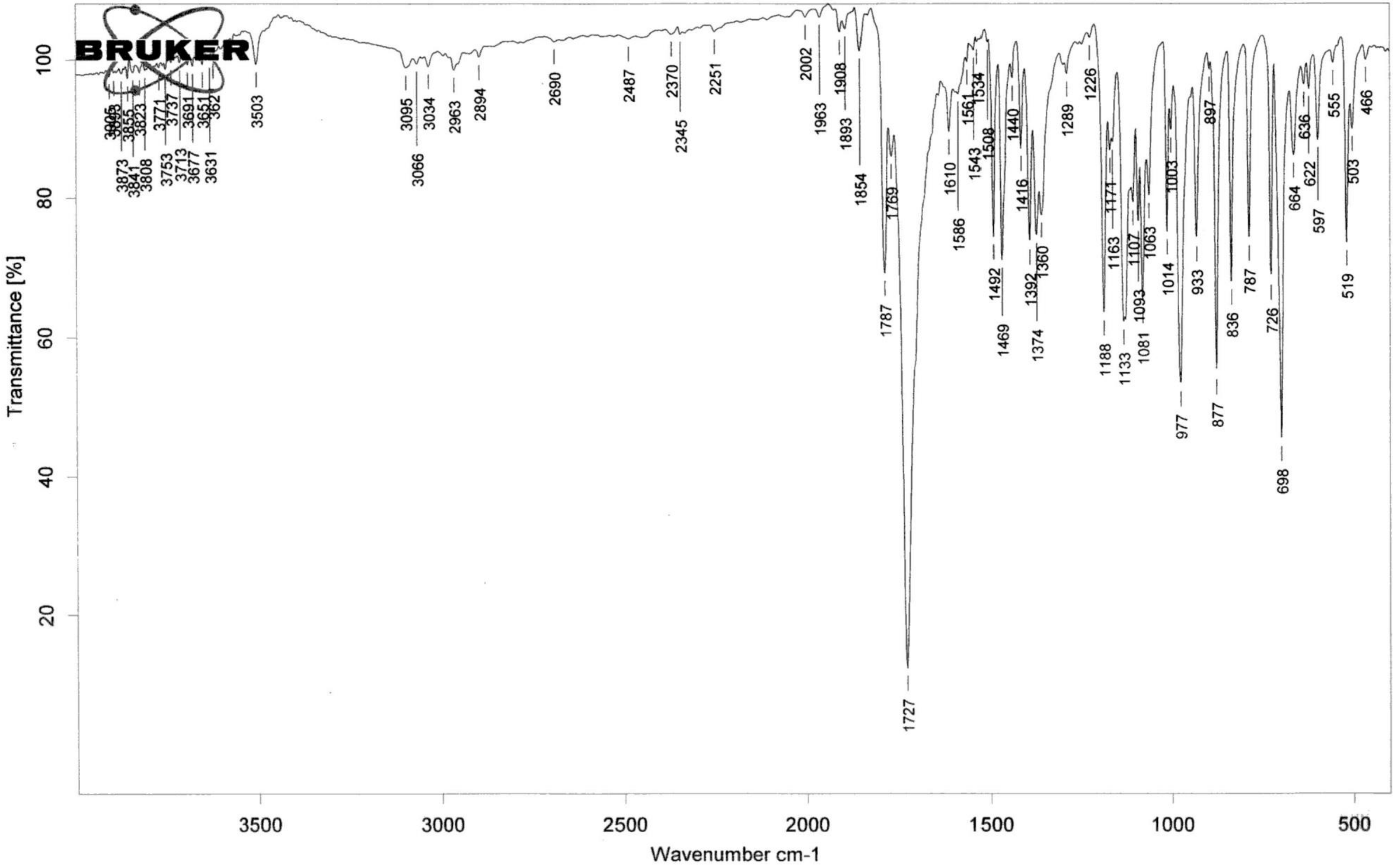

FT-IR (KBr pellet) spectrum of 2-(2-(4-fluorophenyl)-2-iodoethoxy)isoindoline-1,3-dione 3da

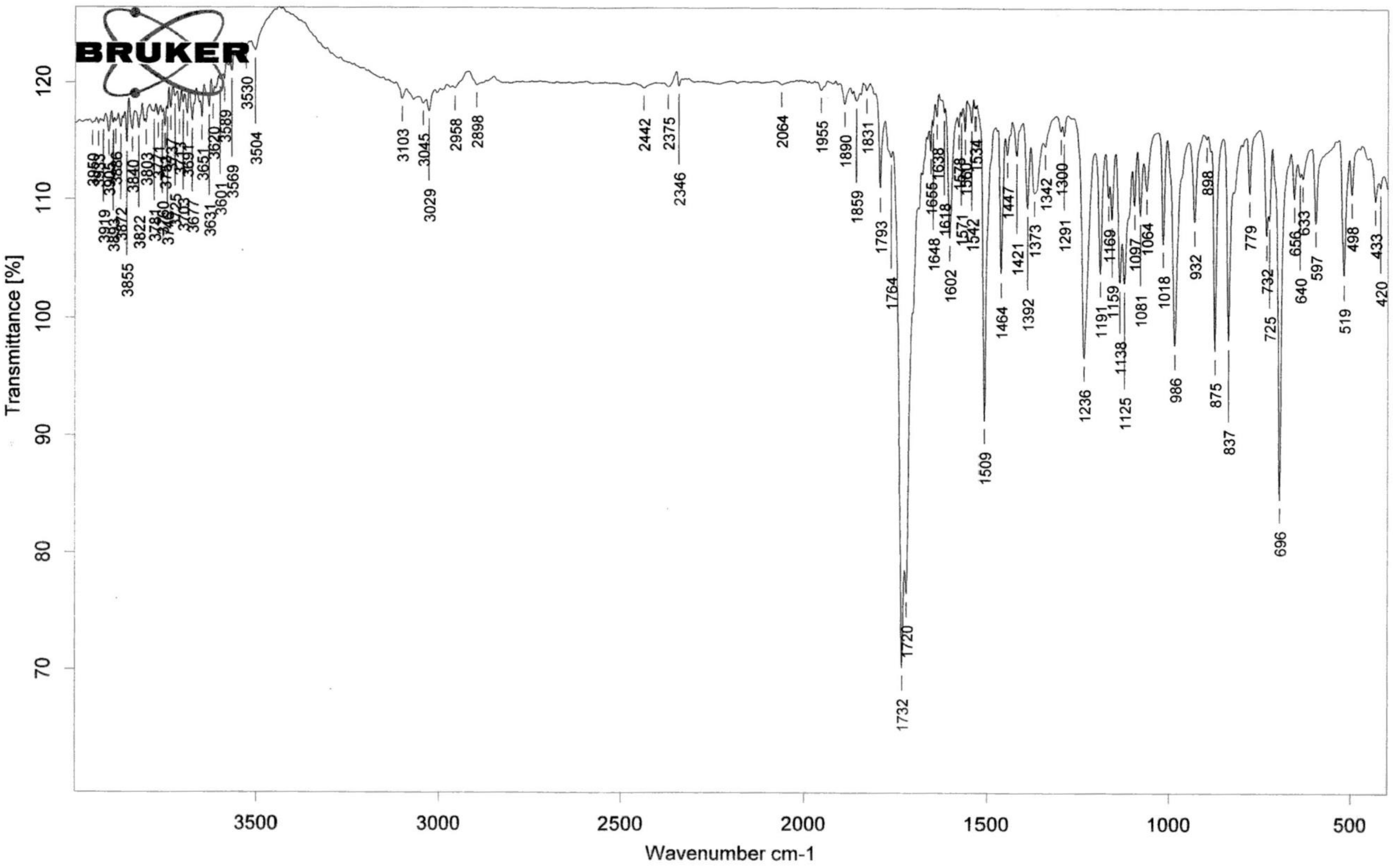

FT-IR (KBr pellet) spectrum of 2-(2-iodo-2-(*m*-tolyl)ethoxy)isoindoline-1,3-dione 3ea

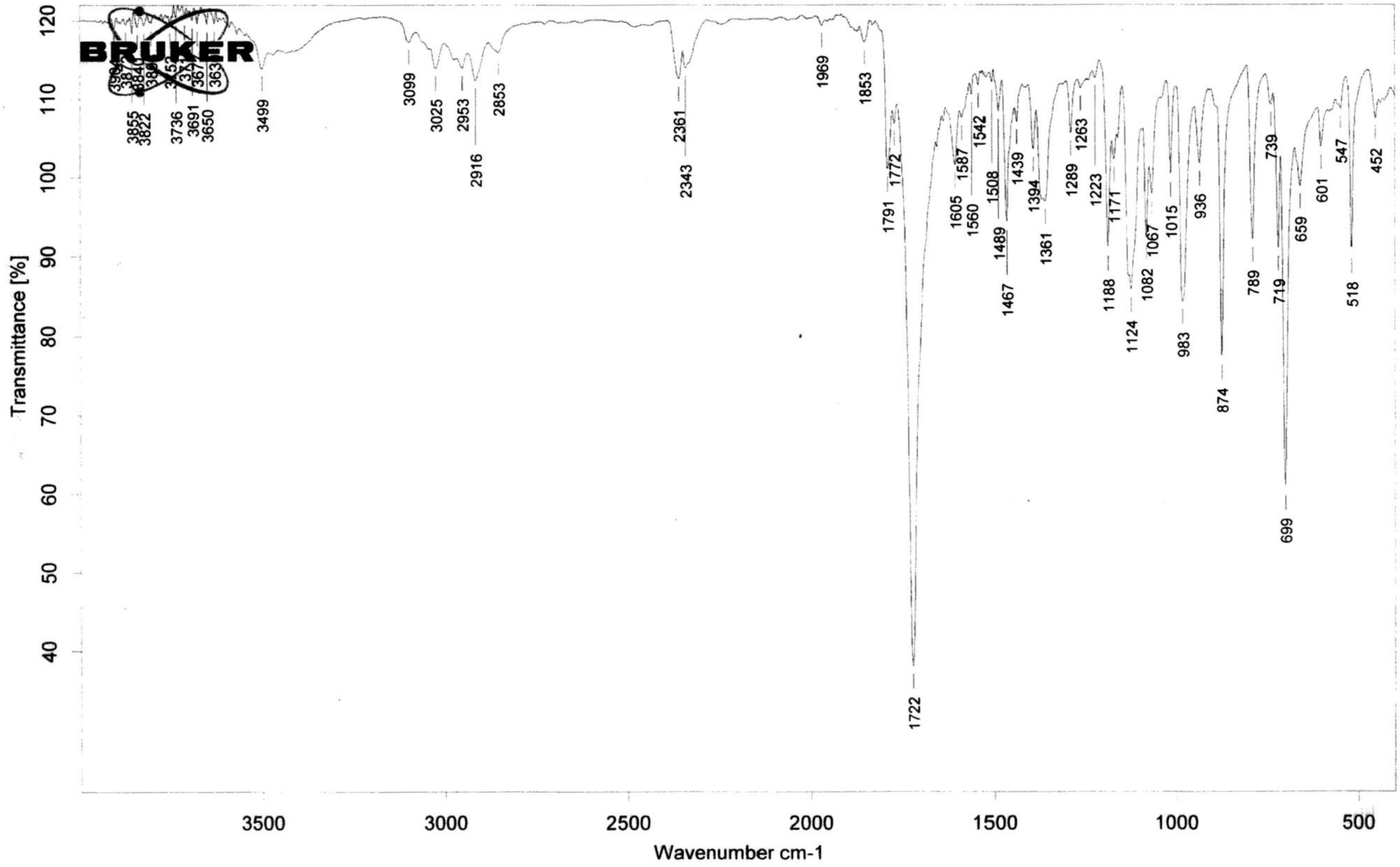

FT-IR (KBr pellet) spectrum of 2-(2-(3-bromophenyl)-2-iodoethoxy)isoindoline-1,3-dione 3fa

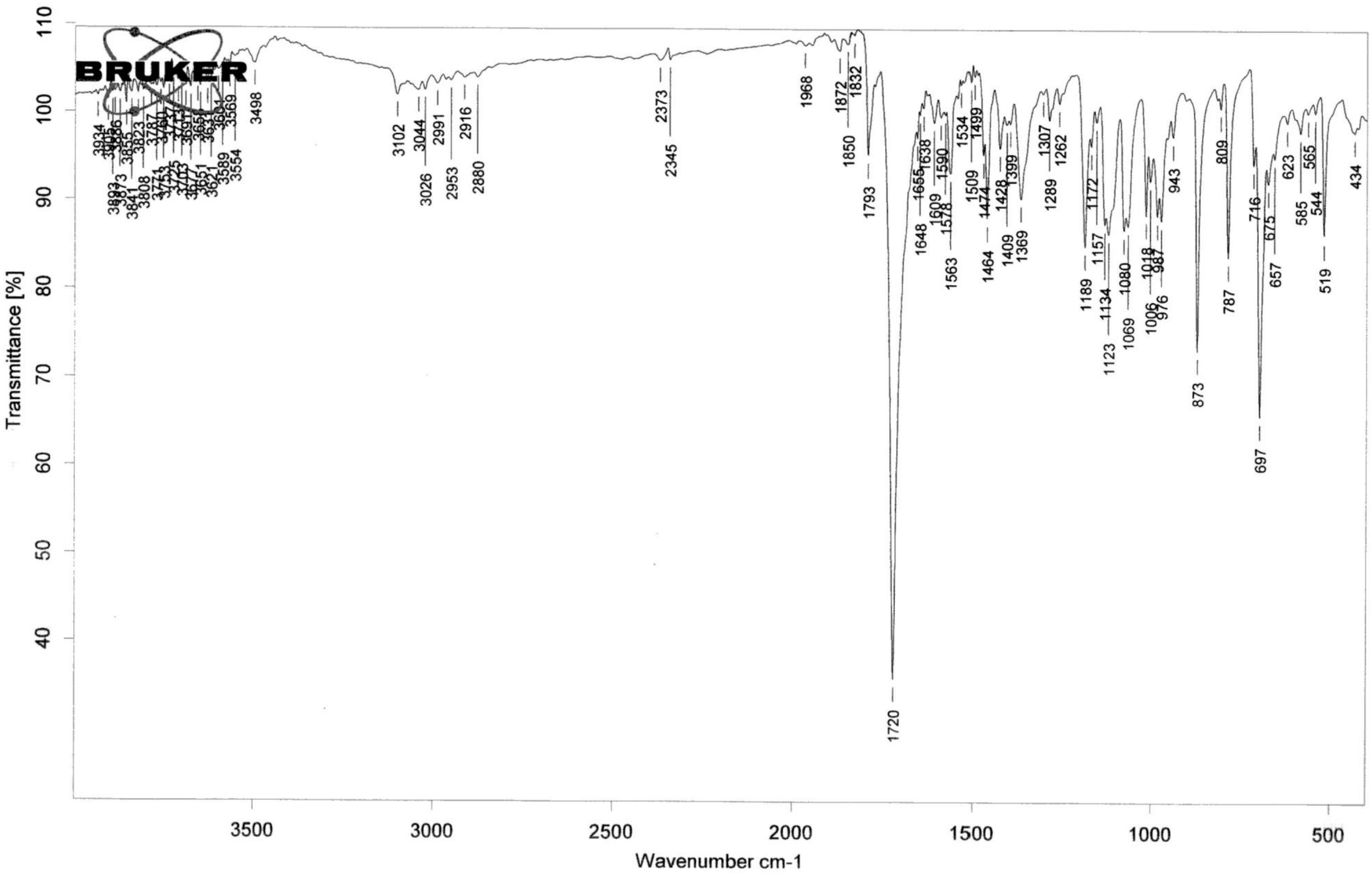

FT-IR (KBr pellet) spectrum of 2-(2-iodo-2-(*o*-tolyl)ethoxy)isoindoline-1,3-dione 3ga

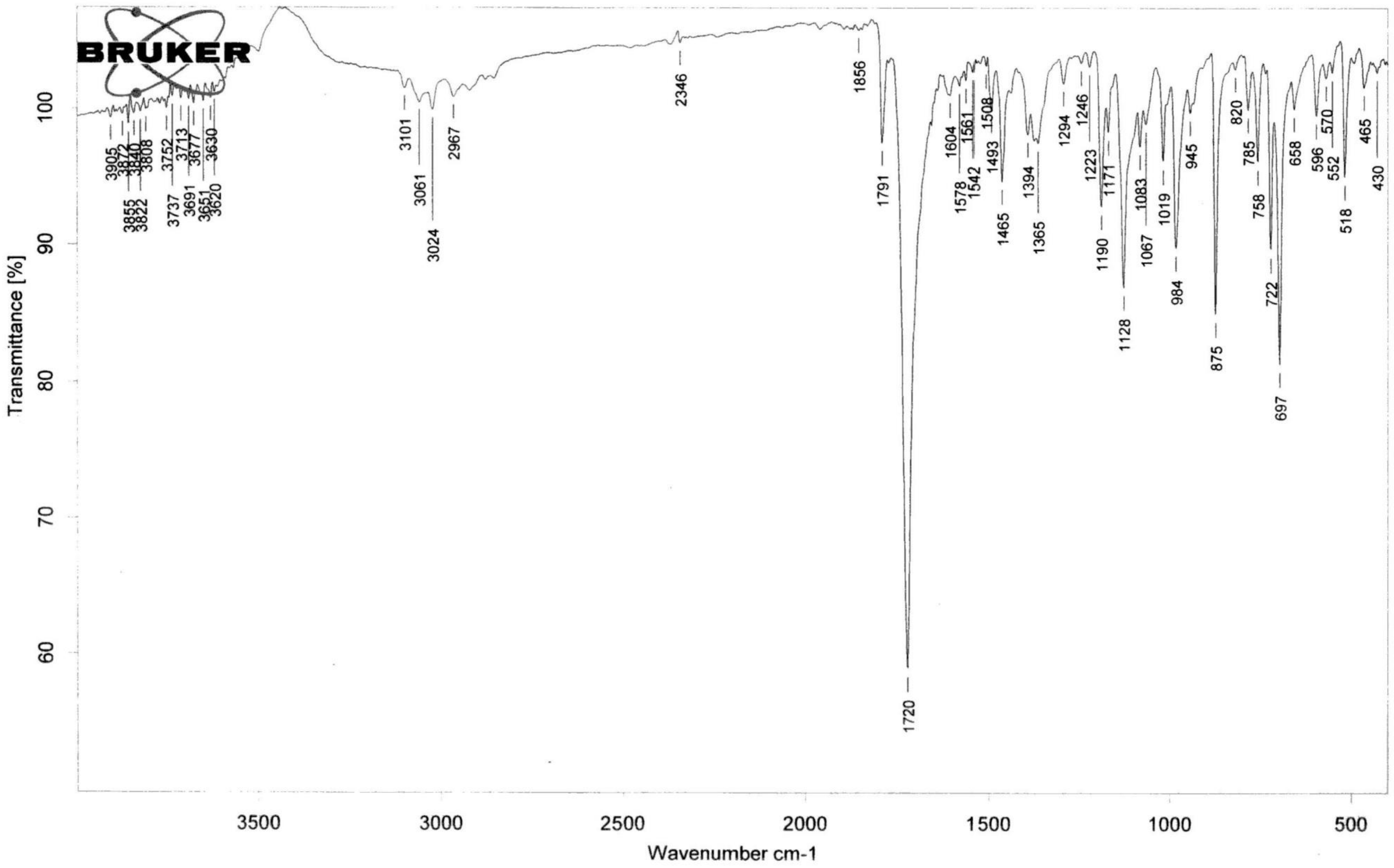

FT-IR (KBr pellet) spectrum of 2-(2-iodo-2-(perfluorophenyl)ethoxy)isoindoline-1,3-dione 3ha

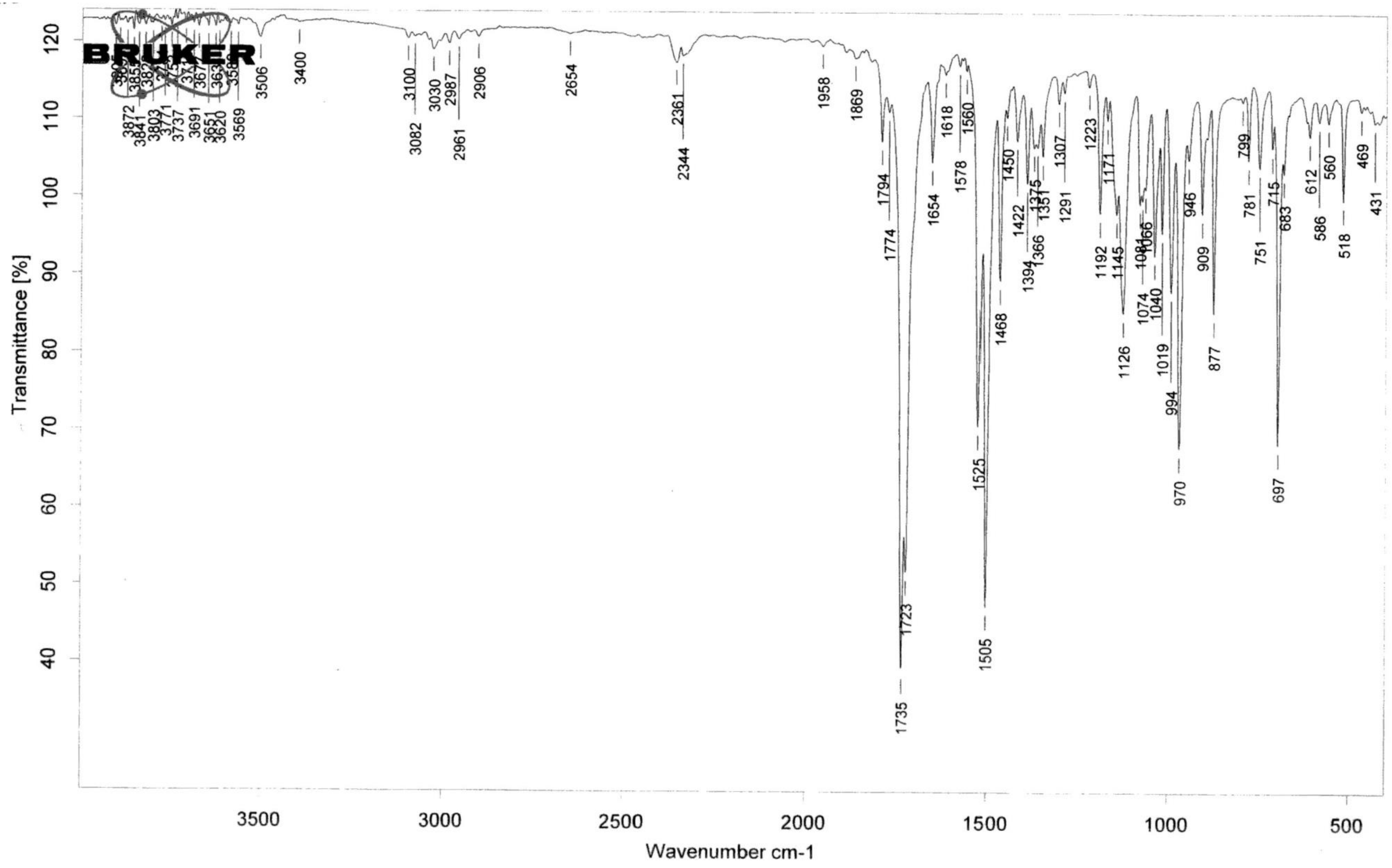

FT-IR (KBr pellet) spectrum of 2-((1-iodo-1-phenylpropan-2-yl)oxy)isoindoline-1,3-dione 3ia

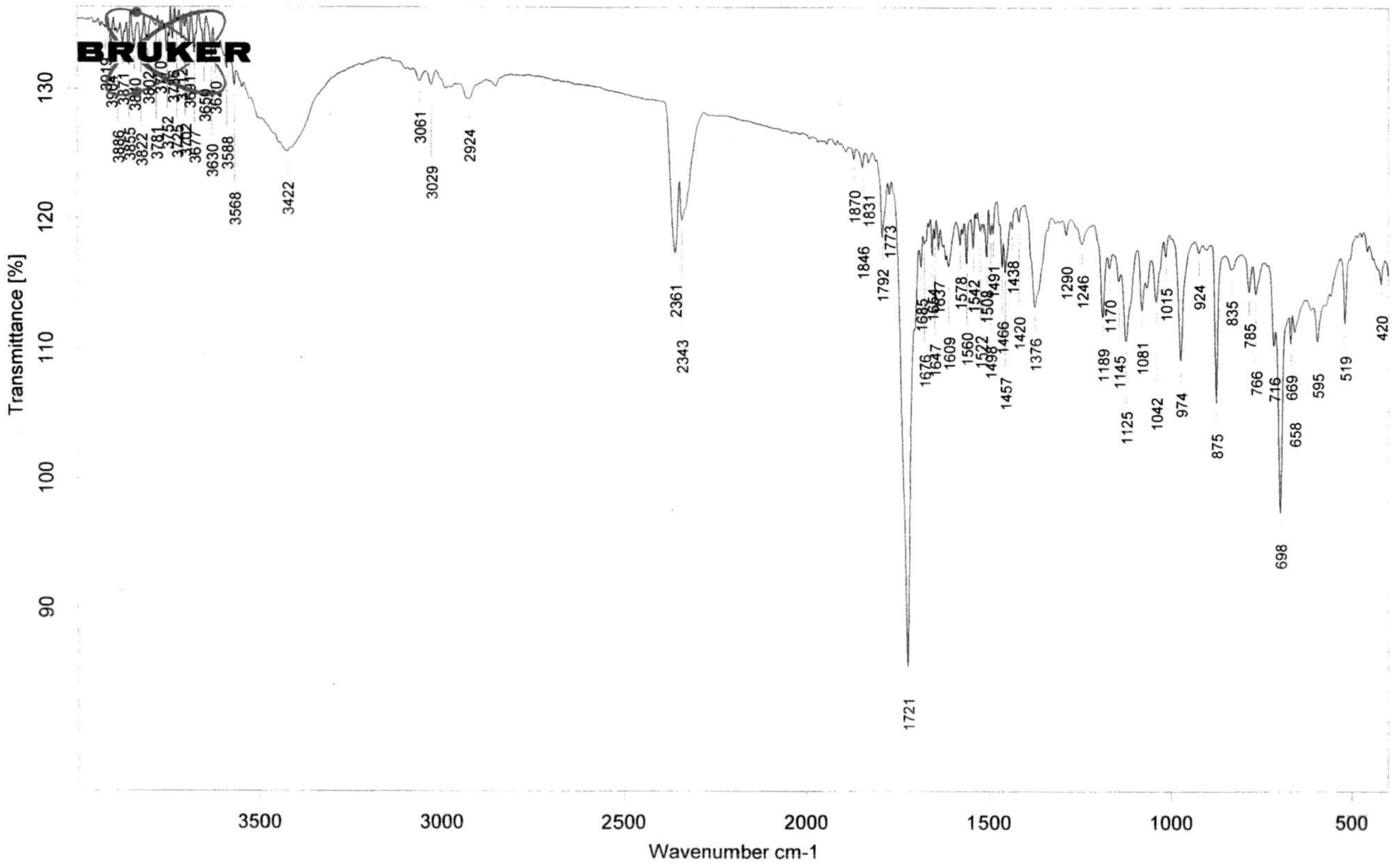

FT-IR (KBr pellet) spectrum of 2-(2-iodo-1,2-diphenylethoxy)isoindoline-1,3-dione 3ja

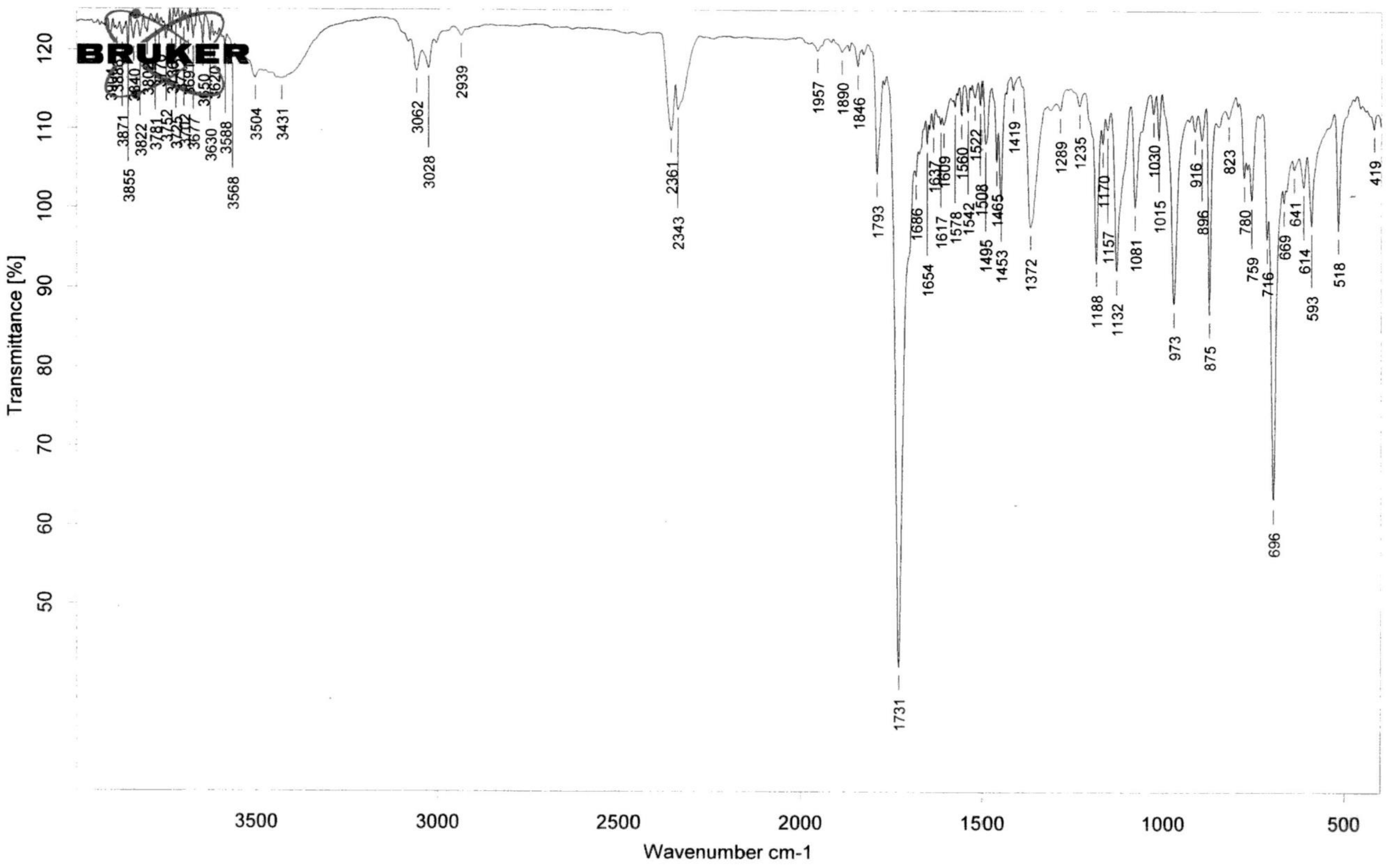

FT-IR (KBr pellet) spectrum of 2-((1-iodo-2,3-dihydro-1H-inden-2-yl)oxy)isoindoline-1,3-dione 3ka

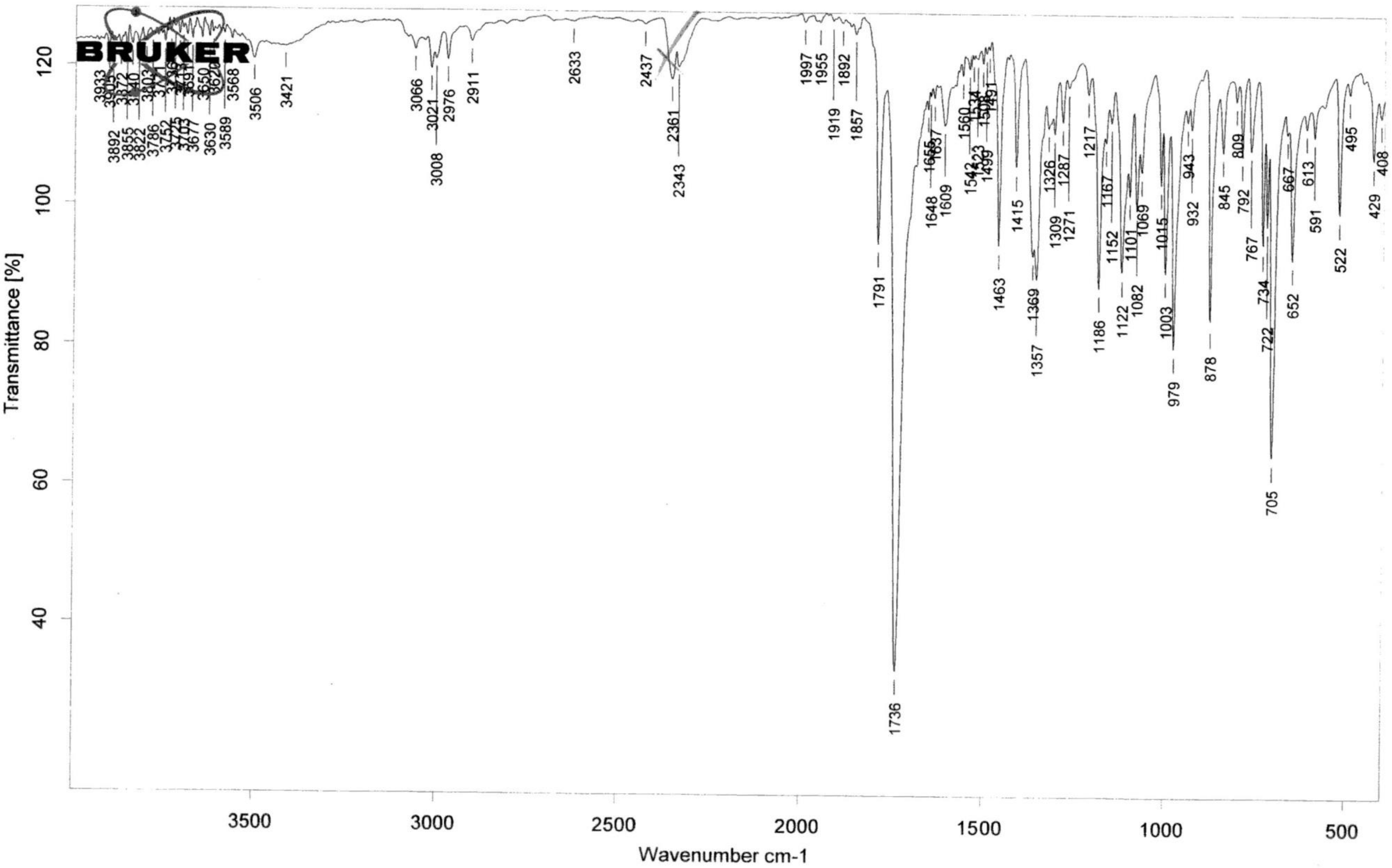

FT-IR (KBr pellet) spectrum of 1-(2-iodo-2-phenylethoxy)pyrrolidine-2,5-dione 3ab

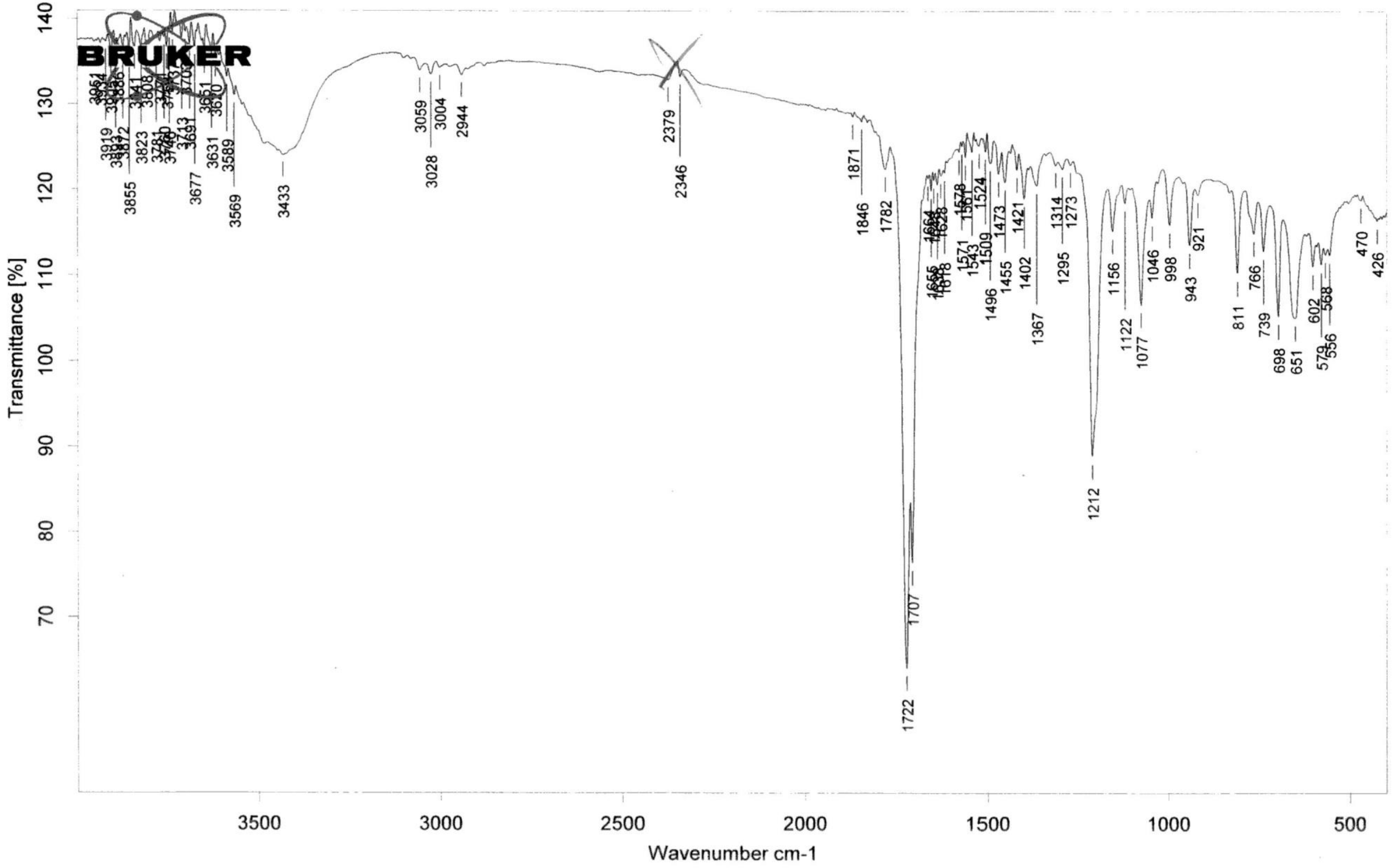

FT-IR (KBr pellet) spectrum of 1-(2-iodo-2-(*p*-tolyl)ethoxy)pyrrolidine-2,5-dione 3bb

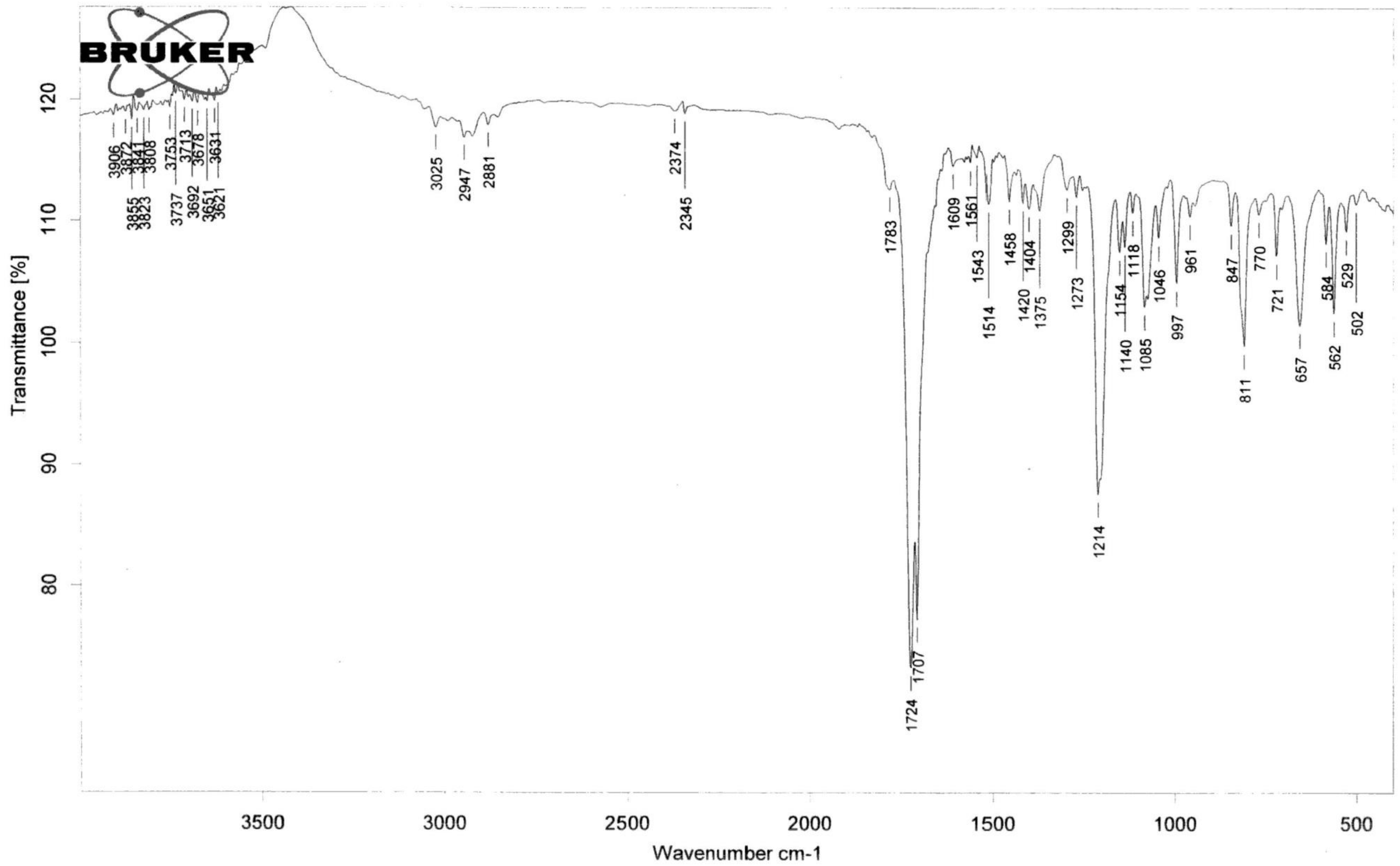

FT-IR (KBr pellet) spectrum of 1-(2-(4-chlorophenyl)-2-iodoethoxy)pyrrolidine-2,5-dione 3cb

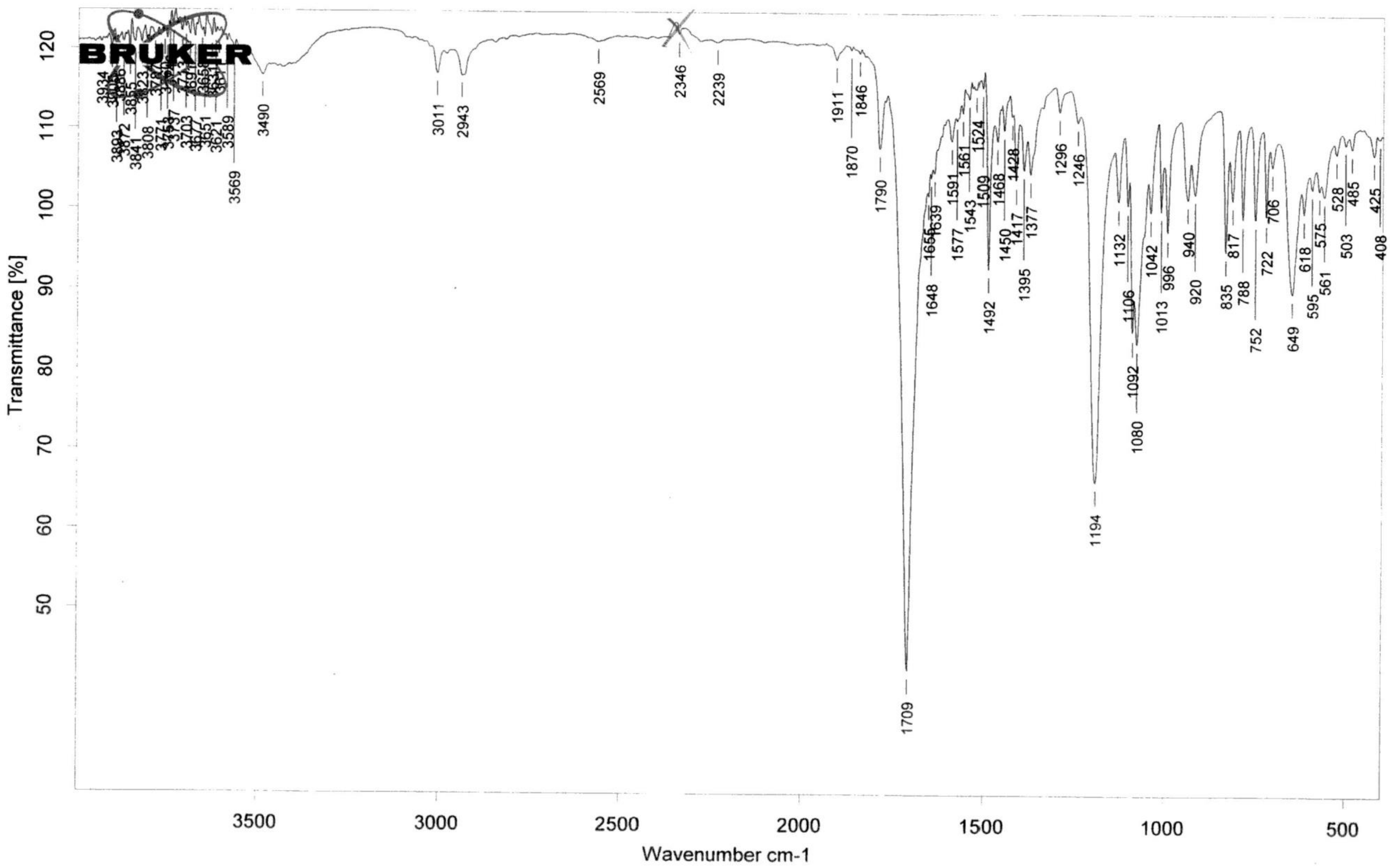

FT-IR (KBr pellet) spectrum of 1-(2-(4-fluorophenyl)-2-iodoethoxy)pyrrolidine-2,5-dione 3db

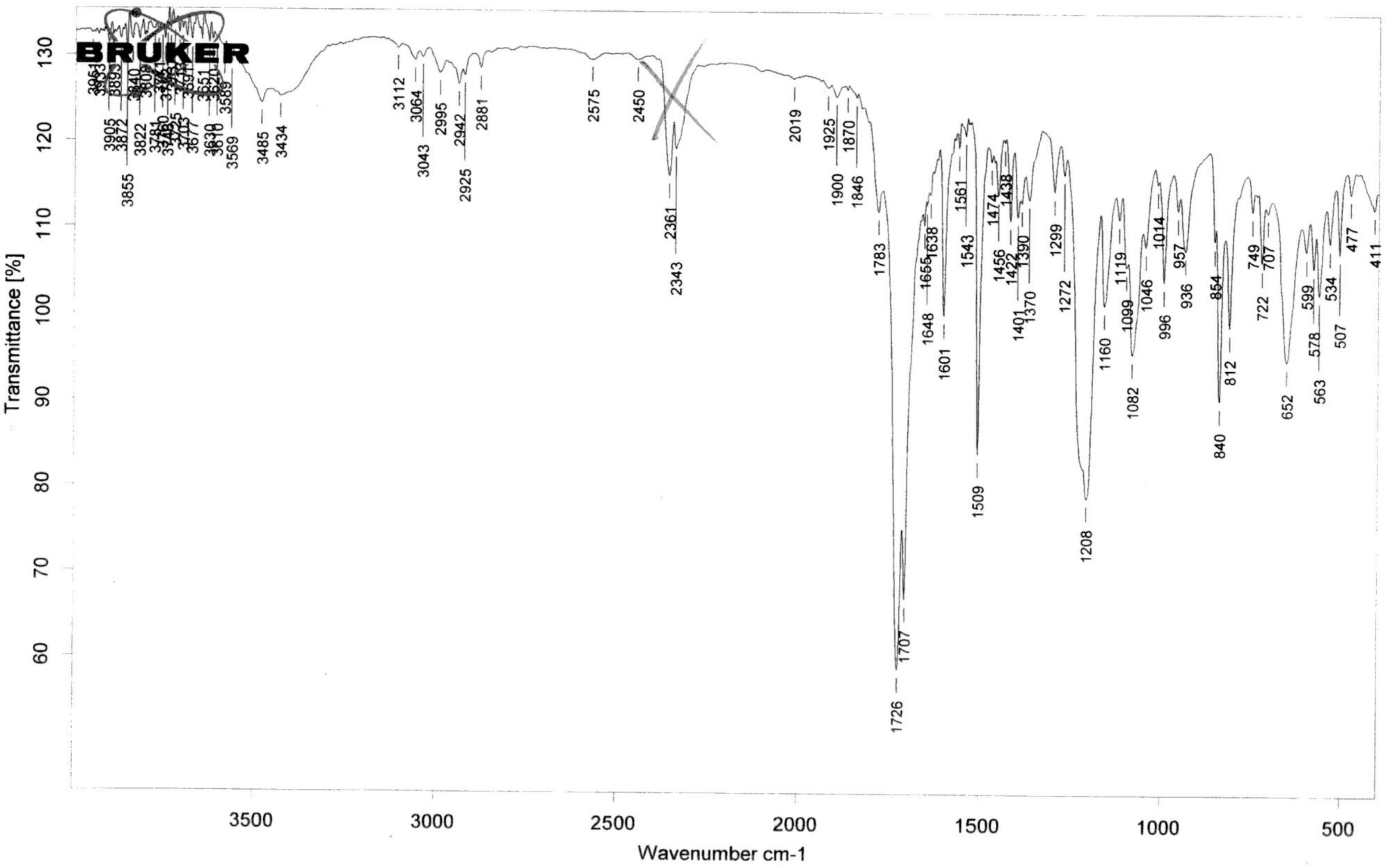

FT-IR (KBr pellet) spectrum of 1-(2-(3-bromophenyl)-2-iodoethoxy)pyrrolidine-2,5-dione 3fb

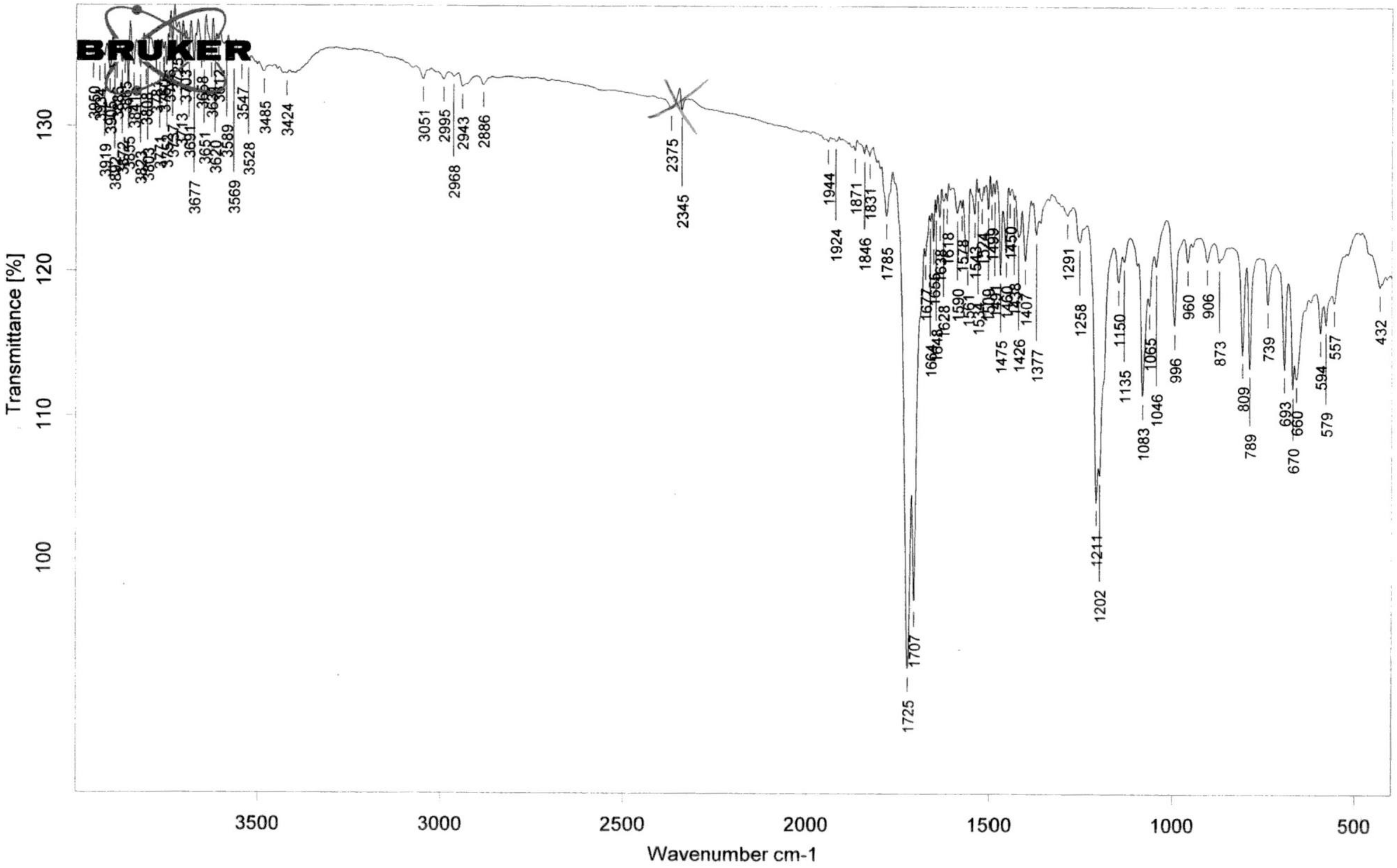

FT-IR (KBr pellet) spectrum of 1-(2-iodo-2-(perfluorophenyl)ethoxy)pyrrolidine-2,5-dione 3hb

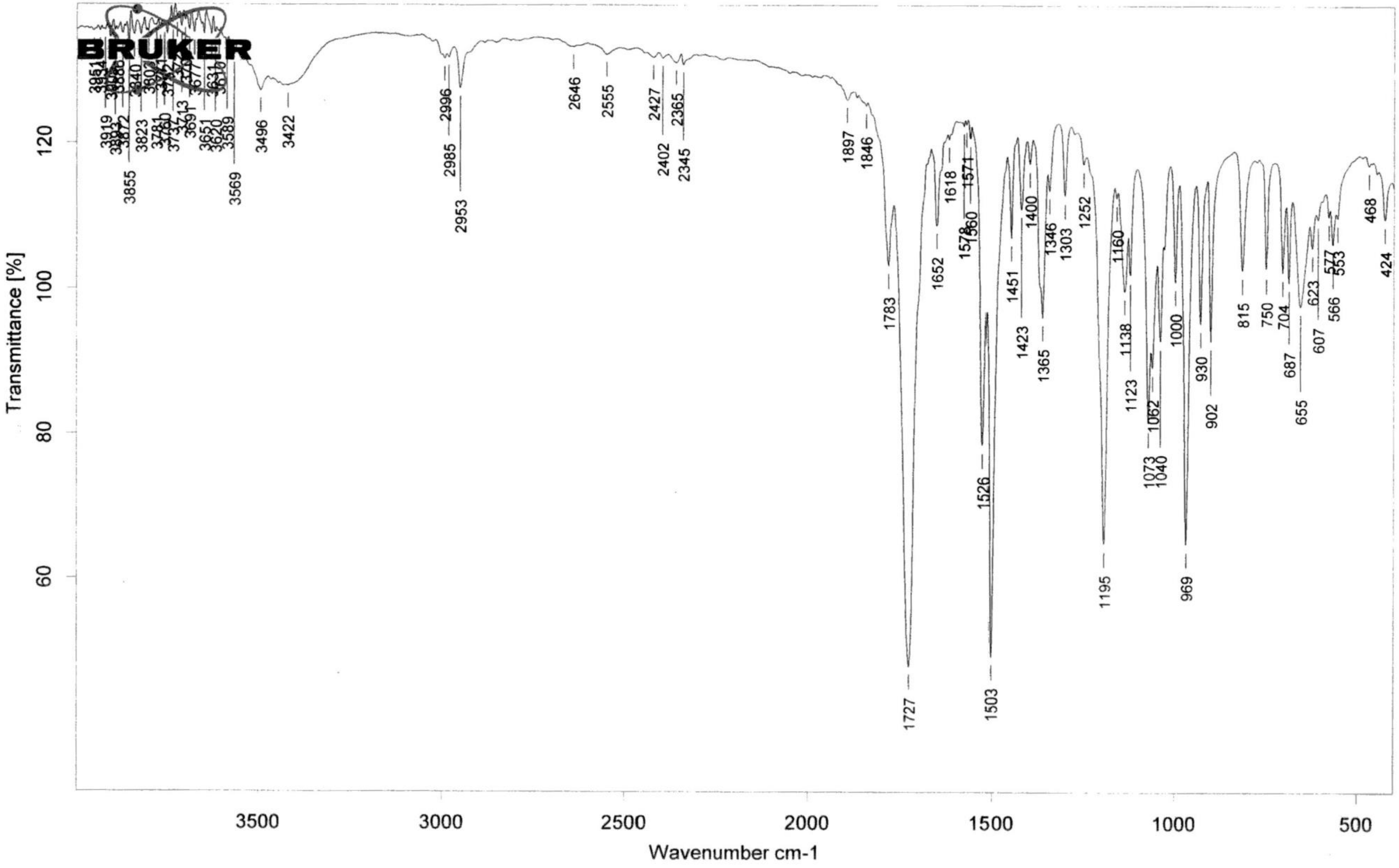

FT-IR (KBr pellet) spectrum of 1-((1-iodo-2,3-dihydro-1H-inden-2-yl)oxy)pyrrolidine-2,5-dione 3kb

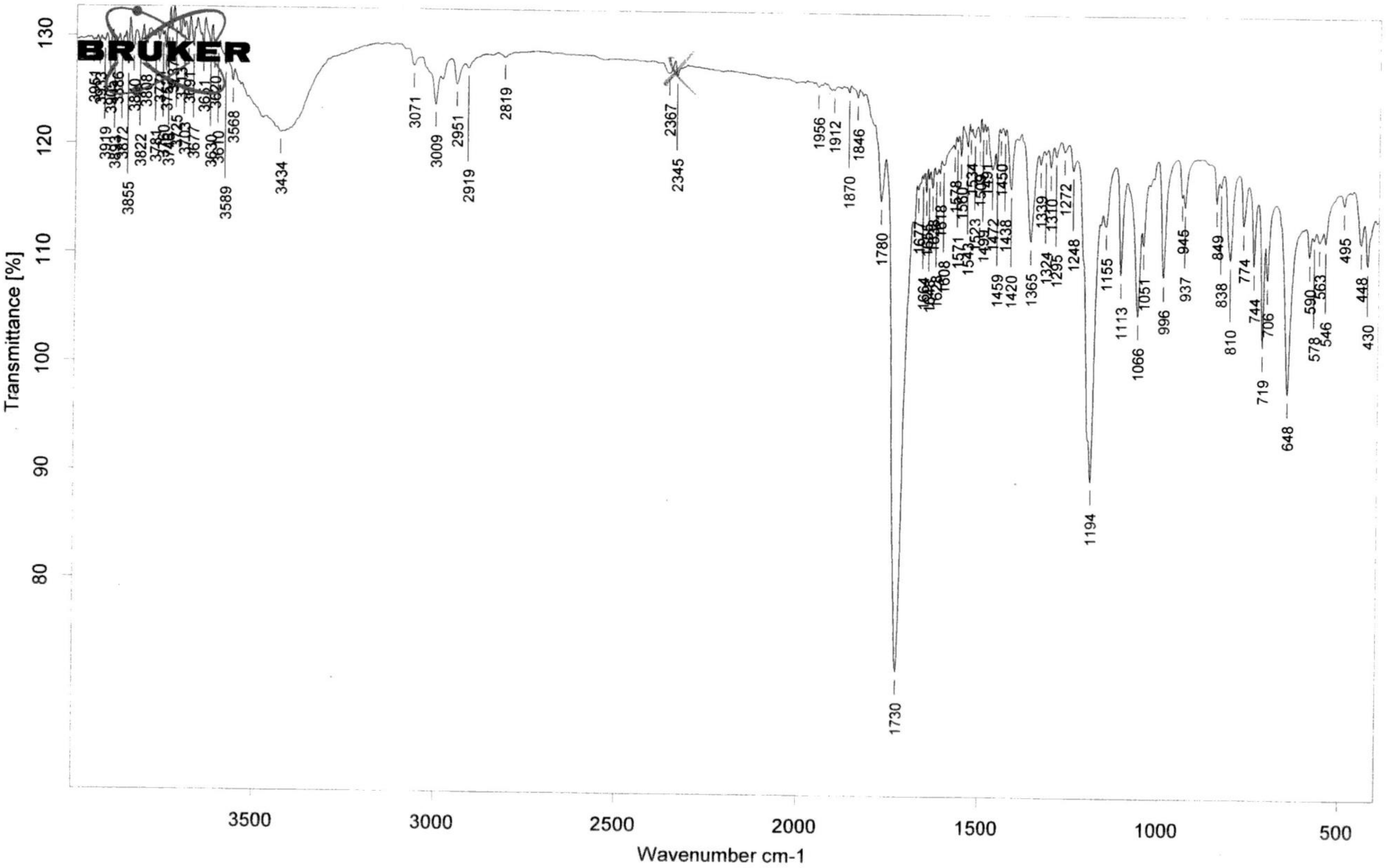

FT-IR (KBr pellet) spectrum of 2-(2-methoxy-2-phenylethoxy)isoindoline-1,3-dione 4a

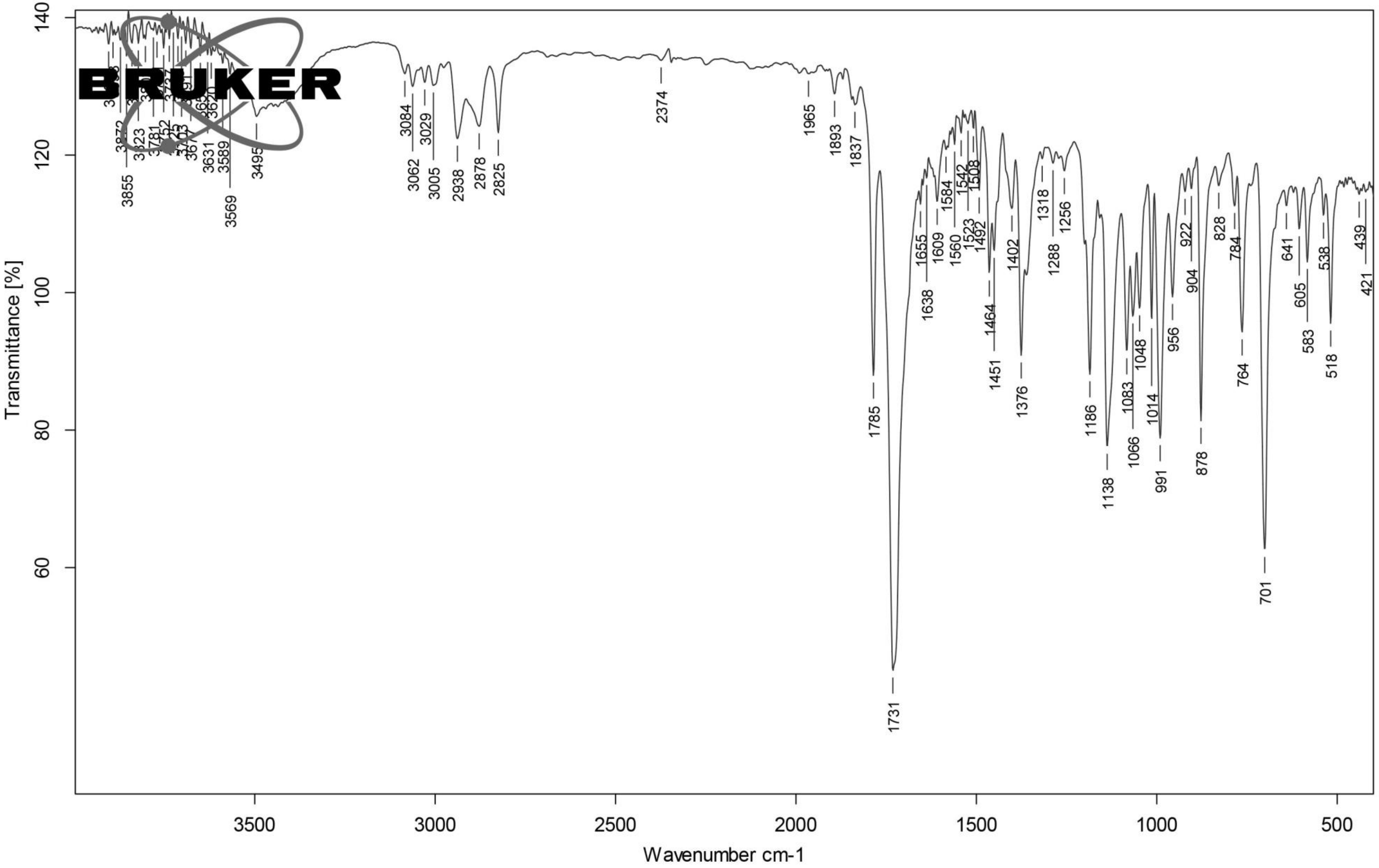

FT-IR (KBr pellet) spectrum of (1-phenylethane-1,2-diyldisulfonyl)dibenzene 4b

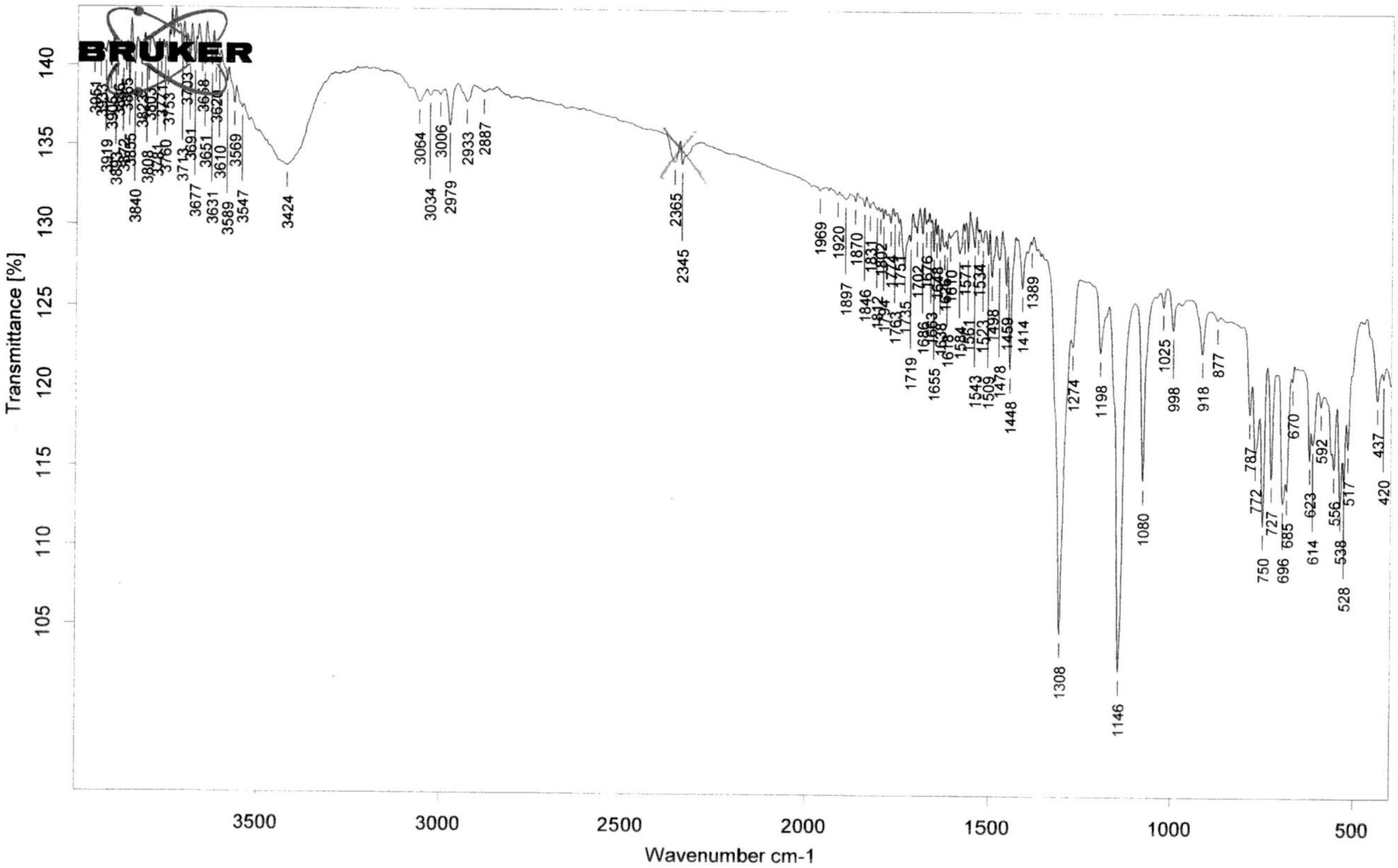

FT-IR (KBr pellet) spectrum of 1-(2-azido-2-phenylethoxy)pyrrolidine-2,5-dione 4c

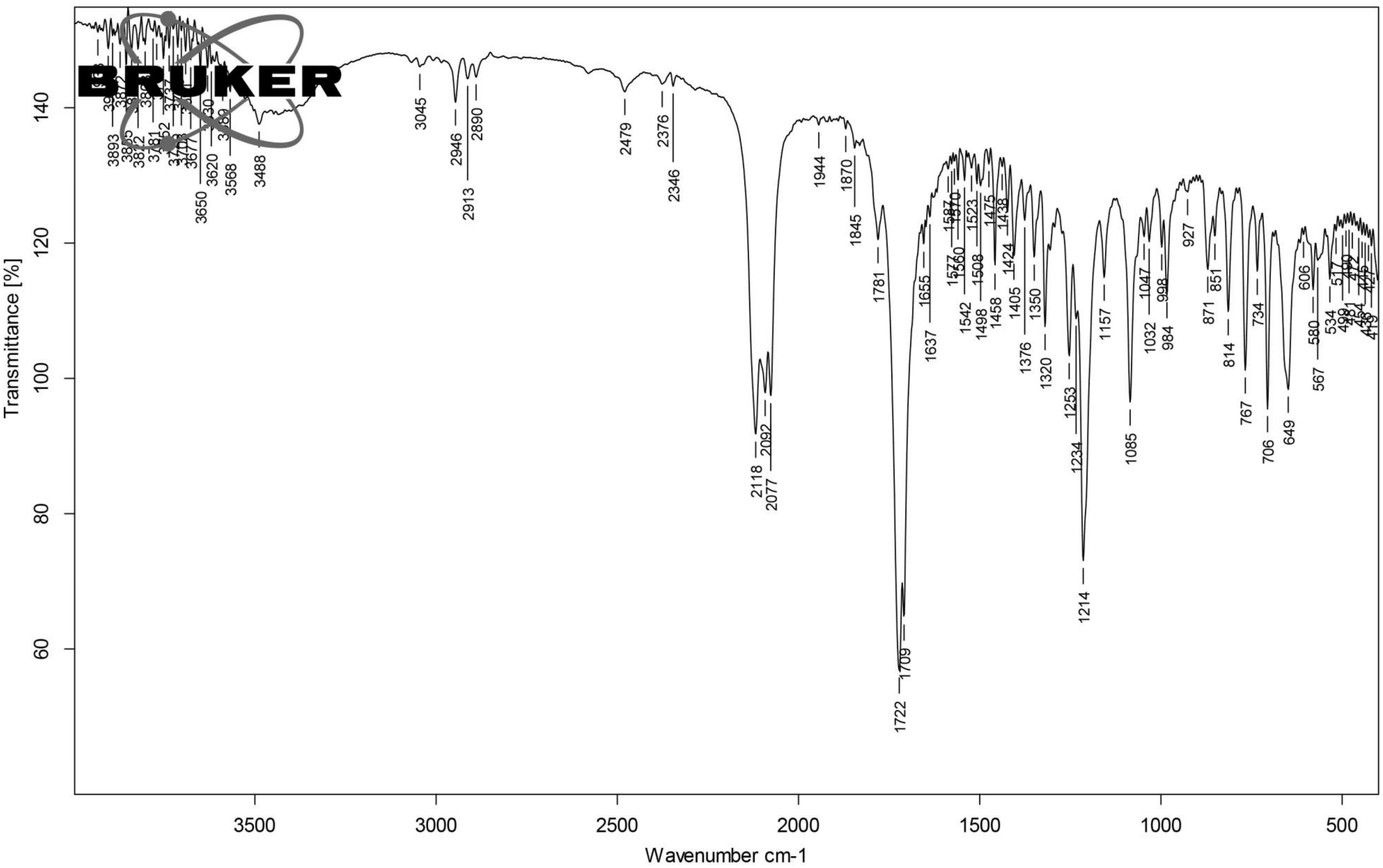

Structure Determination by X-ray Crystallography

Intensity data for **3ca** were collected at the at the K4.4 station (“Belok”) of the Kurchatov Center for Synchrotron Radiation and Nanotechnology in Moscow (Russia) at a wavelength of 0.987 Å using a MAR CCD 165 detector. Data integration was carried out using the CCP4 software [1]. A multi-scan empirical absorption correction was applied to the data using SCALA [1]. The structures were solved by direct methods and refined by the full-matrix least squares method against F^2 of all data, using SHELXL-2014 [2] and OLEX2 [3] software. Non-hydrogen atoms were found on difference Fourier maps and refined with anisotropic displacement parameters with an exception of disordered carbon and oxygen atoms. The positions of hydrogen atoms were calculated and included in refinement in isotropic approximation by the riding model with the $U_{iso}(H) = 1.5U_{eq}(C_i)$ for methyl groups and $1.2U_{eq}(C_{ii})$ for other atoms, where $U_{eq}(C)$ are equivalent thermal parameters of parent atoms. Analysis of Fourier difference maps has shown that Ph groups and ethylene bridge in **3ca** are disordered. The occupancy of corresponding atomic positions was refined as approximately 3:7. The nature of observed disorder can be related to presence of “unbalanced” racemate – a crystalline species, in which the ratio of ordered enantiomers is not unity [4]. Details of data collection and refinement are listed in Table S1. Molecular view of investigated compound in representation of atoms with thermal ellipsoids is given on Figure S1.

References

1. Winn, M. D.; Ballard, C. C.; Cowtan, K. D.; Dodson, E. J.; Emsley, P.; Evans, P. R.; Keegan, R. M.; Krissinel, E. B.; Leslie, A. G.; McCoy, A.; McNicholas, S. J.; Murshudov, G. N.; Pannu, N. S.; Potterton, E. A.; Powell, H. R.; Read, R. J.; Vagin, A.; Wilson, K. S. *Acta Crystallogr., Sect. D: Biol. Crystallogr.* **2011**, *67*, 235. doi: 10.1107/S0907444910045749
2. Sheldrick, G. M. *Acta Crystallogr., Sect. C: Struct. Chem.* **2015**, *71*, 3. doi: 10.1107/S2053229614024218
3. Dolomanov, O. V.; Bourhis, L. J.; Gildea, R. J.; Howard, J. A. K.; Puschmann, H. *J. Appl. Crystallogr.* **2009**, *42*, 339. doi: 10.1107/s0021889808042726
4. Levkin, P. A.; Torbeev, V. Yu.; Lenev, D. A.; Kostyanovsky, R. G. Homo- and Heterochirality in Crystals. In *Topics in Stereochemistry*; Denmark, S. E., Siegel, J. S., Ed.; John Wiley & Sons, Inc.: Hoboken, New Jersey, 2006; Vol. 25, pp 81-134.

Table S1. Crystallographic data for **3ca**

Brutto formula	$C_{16}H_{11}ClINO_3$	d_{calc}, g·cm^{-3}	1.684
Molecular weight	427.61	μ, cm^{-1}	48.84
T, K	100	F(000)	3328
Space group	Fdd2	$2\theta_{max}$, °	70
Z	16	Reflections collected	9382
a, Å	27.181(5)	Independent reflections	2674
b, Å	48.899(10)	Reflections with I>2σ(I)	2095
c, Å	5.0757(10)	Parameters	169
α, °	90	R1 [I>2σ(I)]	0.0715
β, °	90	wR_2 [all data]	0.1728
γ, °	90	GOF	0.987
V, Å^3	6746(2)	Residual electron density, e·Å^{-3} (ρ_{min}/ρ_{max})	1.171/-0.960

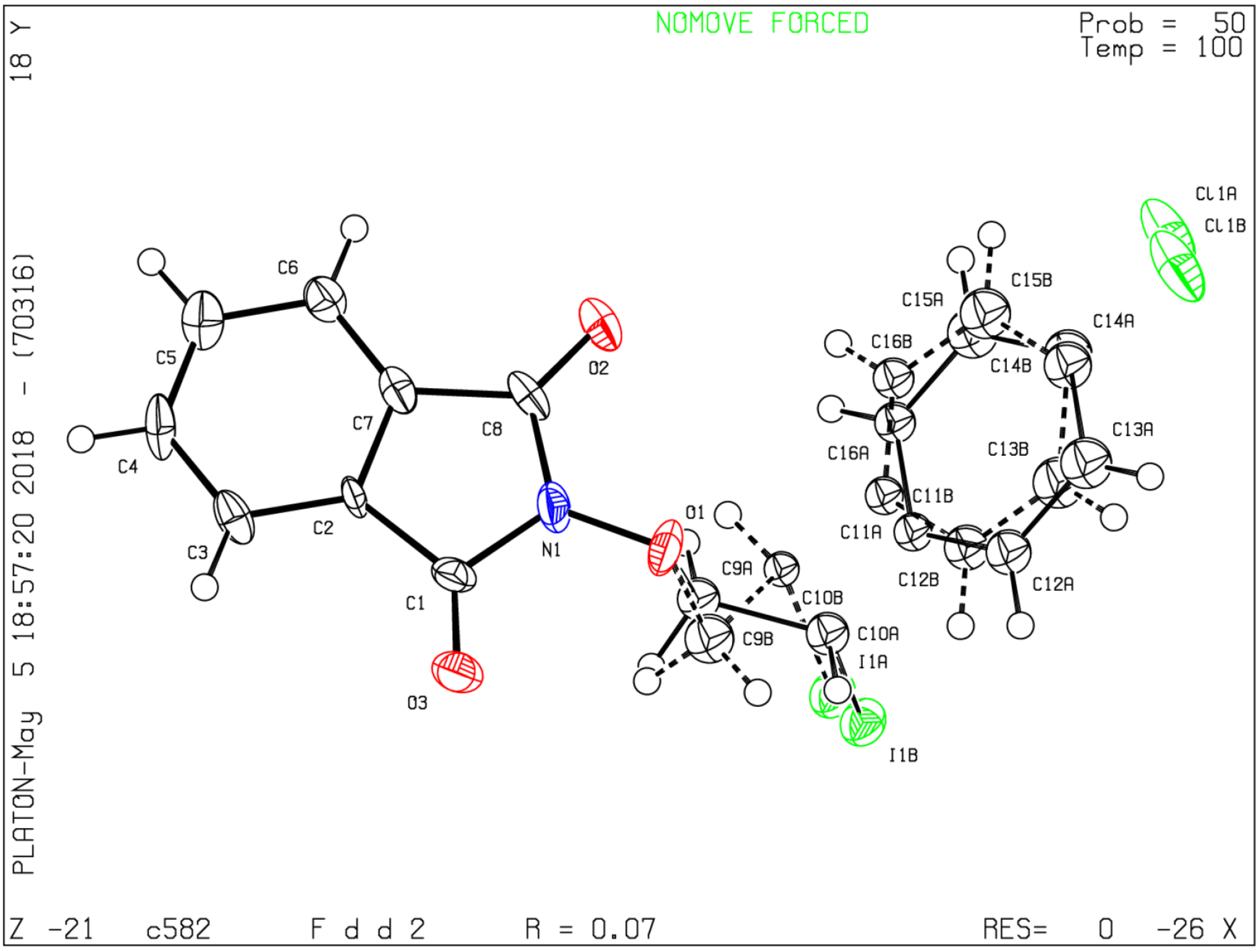

Figure S1. Molecular structure of **3ca**. Atoms are presented as thermal ellipsoids (50% probability).
